# An *in situ* self-assembly template strategy for the preparation of hierarchical-pore metal-organic frameworks

**DOI:** 10.1038/ncomms9847

**Published:** 2015-11-09

**Authors:** Hongliang Huang, Jian-Rong Li, Keke Wang, Tongtong Han, Minman Tong, Liangsha Li, Yabo Xie, Qingyuan Yang, Dahuan Liu, Chongli Zhong

**Affiliations:** 1 State Key Laboratory of Organic-Inorganic Composites, Beijing University of Chemical Technology, Beijing 100029, China; 2 Beijing Key Laboratory for Green Catalysis and Separation, Department of Chemistry and Chemical Engineering, College of Environmental and Energy Engineering, Beijing University of Technology, Pingleyuan 100, Chaoyang, Beijing 100124, China

## Abstract

Metal-organic frameworks (MOFs) have recently emerged as a new type of nanoporous materials with tailorable structures and functions. Usually, MOFs have uniform pores smaller than 2 nm in size, limiting their practical applications in some cases. Although a few approaches have been adopted to prepare MOFs with larger pores, it is still challenging to synthesize hierarchical-pore MOFs (H-MOFs) with high structural controllability and good stability. Here we demonstrate a facile and versatile method, an *in situ* self-assembly template strategy for fabricating stable H-MOFs, in which multi-scale soluble and/or acid-sensitive metal-organic assembly (MOA) fragments form during the reactions between metal ions and organic ligands (to construct MOFs), and act as removable dynamic chemical templates. This general strategy was successfully used to prepare various H-MOFs that show rich porous properties and potential applications, such as in large molecule adsorption. Notably, the mesopore sizes of the H-MOFs can be tuned by varying the amount of templates.

## Abstract (web summary)

The synthesis of porous materials with hierarchically-sized pores is an attractive target for a variety of applications. Here, the authors employ *in situ* generated metal-organic assemblies as metal-organic framework templates which, upon removal, yield single mesoporous-microporous materials.

## Graphical abstract

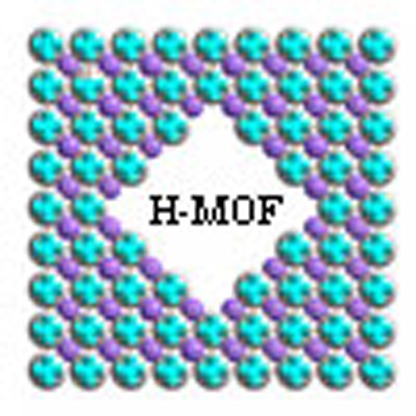

Porous materials have been attracting intense research interest due to their broad range of possible applications^1^. Tailoring structures and pore properties with rational design and controllability of porous materials are crucial for their specific applications, but challenging in practical preparation. Traditional porous solids such as zeolites, activated carbon and mesoporous silica are relatively difficult in modifying and tailoring their structures and functions, in particular at the molecular level, whereas newly developed metal-organic frameworks (MOFs), composed of organic linkers and inorganic nodes, are recognized to be easy in this respect^2^,^3^. This type of materials has shown great potential in various applications including adsorption, separation, catalysis, sensing and so on^4^,^5^,^6^. So far, the related research mainly focuses on the microporous MOFs. In particular, some stable microporous MOFs were reported in recent years^7^,^8^,^9^, which greatly promoted the practical applications of these new materials. The small pore size in microporous MOFs benefits the adsorption and separation of small molecules, but restricts their diffusion and also prevents larger molecules from accessing the MOF channels, thus greatly limiting their applications in some cases^10^. Thereby, the design and preparation of MOF materials with larger pore sizes are imperative, yet challenging to date.

Two approaches have mainly been developed to ‘enlarge' pores of MOFs: (i) construct MOFs by using large building units (metal clusters and/or organic ligands)^11^,^12^,^13^,^14^ and (ii) fabricate MOF materials with large pores as crystal ‘defect'^10^,^15^. With respect to the former, the ligand-extension strategy was widely adopted^16^,^17^,^18^,^19^. However, the pore size in the resulting periodic nanostructures of MOFs is still limited to be smaller than 10 nm (ref. 17). In particular, with the increase of pore size, the frameworks usually become unstable in most cases. On the other hand, the ligand extension normally also results in the interpenetration of the structures, which would dramatically decrease the pore size^16^,^17^. Therefore, increasing the pore size of MOFs and keeping their framework stable remain a great challenge in design and synthesis. In addition, from the viewpoint of cost, synthesis of large linkers is too expensive for practical applications of resulting MOF materials. For the latter approach, the ligands can be cheap but the fabrication methods are pivotal and difficult to follow in most cases.

Alternatively, template methods have been explored to prepare stable hierarchical-pore MOFs (H-MOFs) containing both micropores and mesopores/macropores^20^. As we know that the hard template and the soft template methods have been widely adopted in preparing mesoporous and other hierarchical-pore materials. For the hard template method, as calcinations or acid etchants are often required to remove the templates^21^, MOFs can hardly be kept stable during this process, thus limiting its application in fabricating H-MOFs. For the soft template method, surfactants or block copolymers are used as templates, being easily manipulated and feasible for some MOFs, and thus has been used in preparing some H-MOFs^20^,^22^,^23^,^24^,^25^,^26^. For example, cetyltrimethylammonium bromide has been used as the template to prepare hierarchical-pore Cu_3_(BTC)_2_ (H_3_BTC=1,3,5-benzenetricarboxylic acid) in water–ethanol or ionic liquid systems^20^,^23^,^24^,^25^. Similarly, triblock copolymers such as P123 and F127, which are widely used to prepare meso-porous silicon, have also been used to prepare H-MOFs^27^,^28^,^29^,^30^,^31^. However, as most MOFs were synthesized in polar solvents, such as *N*,*N*-dimethylformamide (DMF), *N*,*N*-dimethylacetamide and *N*,*N*-diethylformamide, traditional surfactants are not able to play the role of a template due to their amphipathy. Therefore, the soft template method also has a limitation in preparing H-MOFs. Other methods for preparing H-MOFs by crystal ‘defect' include gelation^32^, CO_2_-expanded liquids^33^, pseudomorphic replication by transformation of oxide^34^ and so on. However, these innovative approaches are complicated to operate, being comparatively difficult to extend to other MOFs.

For the template method, it is crucial to select appropriate template in the preparation of porous materials. The template should not only have a good interaction/affinity with reaction precursors but also should be able to remove easily, keeping the structure of targeted porous materials intact. In addition, for some practical applications, bad stability of a material often is viewed as extremely negative. However, the weakness of the instability in a material can also be a positive factor in some cases^35^. Motivated by the context described above, herein we propose to use metal-organic assemblies (MOAs) including MOFs as the templates to prepare H-MOFs through an *in situ* self-assembly approach, where both the targeted porous materials and the templates belong to coordination complexes, being compatible in structural nature and reaction activity. Simultaneously, we can take advantage of their relatively different stabilities to get desired H-MOFs, that is, unstable MOAs as templates and stable MOFs as parents for targeting H-MOFs.

It has been well-documented that although MOF-5 is stable in several solvents^16^, the sensitivity towards moisture and acid can lead to its structural collapse and decomposition^36^,^37^. In contrast, some MOFs are chemically, thermally and mechanically stable, such as UiO-66(Zr)^38^, which can maintain its crystal structure even in an acid solution. These differences in the stability stimulate us to try to use water- or acid-sensitive MOFs, such as MOF-5 as the potential template to synthesize stable H-UiO-66(Zr).

Here we suppose that if this hypothesis can be accomplished, a lot of labile MOAs could be used as template sources for the preparation of various stable H-MOFs. Theoretically, in a self-assembly reaction process, the reversibility of coordination bonds in these coordination complexes could keep the MOAs template forming and disappearing during the stable MOFs forming and growing. Thus, the MOAs could indeed act as a chemical dynamic template to direct the construction of H-MOFs. In this work, we demonstrate this idea, a new template-based strategy, to prepare stable H-MOFs by adopting an *in situ* self-assembly synthesis method (Fig. 1).

## Results

### Preparation of H-MOFs through two-step reactions

**Figure 2:**
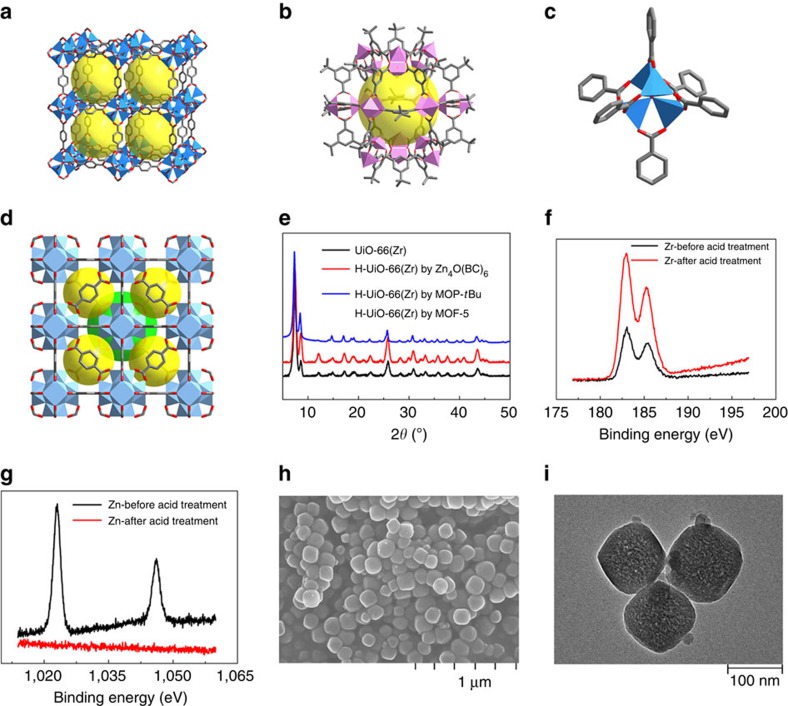
Template-based preparation and characterization of H-UiO-66(Zr). (**a**) The structure of MOF-5, (**b**) the structure of metal-organic polyhedron (MOP-*t*Bu), (**c**) the structure of Zn_4_O(BC)_6_ and (**d**) the structure of UiO-66(Zr). Colour scheme: Zn atom, light blue polyhedron; Cu atom, pink polyhedron; Zr atom, cinerous polyhedron; C atom, grey; and O atom, red. All H atoms have been omitted for clarity. The yellow and green spheres represent the void inside of MOF and MOP. (**e**) PXRD patterns of H-UiO-66(Zr) prepared using different template precursors. (**f**,**g**) The XPS spectra of template@H-UiO-66(Zr) and H-UiO-66(Zr) prepared with MOF-5 as the template precursor over the Zr 3*d* and Zn 2*p* spectral regions, respectively. (**h**) SEM image and (**i**) transmission electron microscope image of H-UiO-66(Zr) prepared with MOF-5 as the template precursor.

A series of proof-of-concept experiments were performed. First, we used MOF-5 (Fig. 2a) as a template precursor to prepare H-UiO-66(Zr) (see Fig. 2d for the structure of UiO-66(Zr)) through a two-step reaction process. Nano-sized MOF-5 particles were first synthesized and mixed with ZrCl_4_ and terephthalic acid in DMF. The mixture was then heated under solvothermal reaction condition, similar to that for synthesizing UiO-66(Zr). After the reaction, the resulting product template@H-UiO-66(Zr) was washed with acid aqueous solution to get targeted material H-UiO-66(Zr). Powder X-ray diffraction (PXRD) measurements show that the resulting H-UiO-66(Zr) has the same diffraction patterns as the parent UiO-66(Zr) (Fig. 2e and Supplementary Fig.18). The N_2_ adsorption at 77 K demonstrates the formation of the hierarchical-pore material with both micropores and mesopores (Supplementary Fig. 5). The detail of the preparation process can be seen in the Supplementary Methods section.

Considering such a fact that the mesopore size (about 11 nm) of the H-UiO-66(Zr) is much smaller than that of originally added MOF-5 particles with the size in the range of 370∼520 nm (Supplementary Figs 1 and 2, the evaluated polydispersity index is 0.1), we suspect that there exists a ‘decomposition and/or rearrangement' of MOF-5 particles in the reaction system. This is indeed coincident with the characteristic of MOF-5 as a typical coordination complex, where MOF-5 particles were in a self-assembly stage of dynamic equilibrium due to the reversibility of the involved coordination bonds^39^,^40^. The general elemental analysis (EA) and inductive-coupled plasma (ICP) emission spectra analysis for the template@H-UiO-66(Zr) gave the contents of Zr 27.14, Zn 11.05, C 35.65% and H 1.57%; however, for H-UiO-66(Zr) they are Zr 31.24, Zn 0.24, C 33.49% and H 2.27%, respectively. The mole ratio of C/Zn in the ‘template' was estimated (by assuming that all the Zr comes from H-UiO-66(Zr) and H-UiO-66(Zr) has the same element contents as that parent UiO-66(Zr)) to be 3.5, being smaller than that in MOF-5 (theoretical value is 6) (Supplementary Table 2). This result indicates that in the formation of H-UiO-66(Zr), the actual template is not MOF-5 particles but is some newly *in situ*-regenerated MOAs. This judgment can also be supported by the fact that no diffraction peaks of the MOF-5 were observed in template@H-UiO-66(Zr) (Supplementary Fig. 19). Besides, it was also found that the MOF-5 particles decompose within 1 h in the ZrCl_4_ DMF solution even at room temperature, which further reveals the ‘decomposition and/or rearrangement' of MOF-5 in the reaction system. On the basis of these observations, the fact of a complicated self-assembly process could be thus justified. These generated MOAs act thus as the ‘templates' to direct the formation of template@UiO-66(Zr). During acid treatment, the ‘template' was removed. Therefore, the acid treatment indeed acts as an activation process to clear the pores of the H-MOF.

As shown in Fig. 2f,g, X-ray photoelectron spectroscopy (XPS) spectra demonstrate that the template@H-UiO-66(Zr) sample contains both Zn and Zr, but after acid treatment almost no Zn was detected (on the sample surface), while Zr remained. Based on the ICP analysis, a little bit Zn was identified in the H-UiO-66(Zr), which might be attributed to the Zn ions anchoring (through coordinating with some groups, such as carboxylate) in mesopore surfaces of H-UiO-66(Zr). These coordination groups were generated from the ‘defect' of the UiO-66(Zr) framework. In addition, as given above, the C and H contents in H-UiO-66(Zr) were close to the theoretical values (C 34.6% and H 1.68%) of parent UiO-66(Zr). Moreover, the Fourier transform infrared (FT-IR) spectrum indicates no free terephthalic acid remained in the pores of H-UiO-66(Zr) (Supplementary Fig. 31).

Furthermore, high-angle annular dark-field scanning transmission electron microscopy (HAADF-STEM) analysis combined with energy dispersive X-ray spectroscopy (EDX) mapping reveals that Zr, O and C elements were homogeneously distributed in the whole template@H-UiO-66(Zr) sample but Zn element was heterogeneous (Supplementary Fig. 40). In addition, scanning electron microscope (SEM) and transmission electron microscope images show that the H-UiO-66(Zr) sample is of small nanoparticles (∼100 nm) and has a sponge-like morphology, which implies the existence of crystal defect (Fig. 2h,i). These results suggest the existence of irregularly distributed mesopores in H-UiO-66(Zr). All these results confirm the existence of the Zn-based templates in template@H-UiO-66(Zr) and these templates can be almost completely removed by the acid treatment to give the H-MOF material.

In addition, there exist hysteresis loops in the N_2_ adsorption isotherms of template@H-UiO-66(Zr) and H-UiO-66(Zr), being indicative of mesoporous structures of both materials (Supplementary Fig. 6). Before the acid treatment, the mesoporous structure may be created by removal of part of soluble template fragments in the sample treatment process through the DMF washing. However, the surface area and pore volume are low in template@H-UiO-66(Zr). After acid treatment, the material became clearly more porous and the mesopore was much larger than that before treatment. This result also implies that the acid treatment indeed is just an activation process to clear the pores as discussed above. It should also be pointed out that although UiO-66(Zr) is not stable in phosphoric acid solution^41^, it is stable in diluted HCl solution^38^. As shown in Supplementary Fig. 8, N_2_ adsorption isotherms indicate no mesopore in UiO-66(Zr) after soaking in diluted HCl solution (pH 1) for 12 h.

To further confirm the associated self-assembly process in the preparation of H-MOFs using this new method and its universal accessibility, another two types of MOAs were also chosen as the template precursors to carry out the synthesis experiments, including simple complex molecules and metal-organic polyhedra (MOPs) molecules. These molecule-based MOAs can be used as the supramolecular building units in constructing MOFs^16^,^42^,^43^. For the former, we explored Zn_4_O(BC)_6_ (BC, benzenecarboxylate; Fig. 2c) to again prepare H-UiO-66(Zr). Zn_4_O(BC)_6_ is a primary coordination complex about 2 nm in size, which is the secondary building unit of MOF-5 structure. Specifically, the Zn_4_O(BC)_6_ was first prepared by the reaction of Zn(NO_3_)_2_·6H_2_O and benzoic acid in DMF. After the reaction, the resulting solution was cooled down to room temperature. Then, ZrCl_4_ and terephthalic acid were added and the mixture was heated under reaction conditions similar to those for the synthesis of parent UiO-66(Zr). Figure 2e and Supplementary Fig. 16 show that the resulting material also has the same PXRD pattern as that of the parent UiO-66(Zr). After washing with acid, the resulting H-UiO-66(Zr) was harvested as confirmed by the PXRD, XPS spectra, ICP, EA, thermal gravimetric analysis (TGA) and N_2_ adsorption/desorption isotherms (Supplementary Figs 3,16,24 and 36). XPS spectrum indicates that almost no Zn exists in the H-UiO-66(Zr) sample surface after acid treatment. The EA and ICP analyses gave the contents of Zr 27.95, Zn 12.75, C 45.32 and H 3.15% in the template@H-UiO-66(Zr); however, for H-UiO-66(Zr) they are Zr 31.08, Zn 0.25, C 33.12 and H 2.05%. The evaluated mole ratio of C/Zn of the ‘template' in template@H-UiO-66(Zr) is 6.8, which is again much smaller than that in Zn_4_O(BC)_6_ molecule (theoretically, the mole ratio of C/Zn is 10.5). This result indicates that there is a ‘decomposition and/or rearrangement' of the Zn_4_O(BC)_6_ molecule in the *in situ* self-assembly reaction, the Zn_4_O(BC)_6_ is not the true template. In addition, as given above the C and H contents in H-UiO-66(Zr) close to the theoretical value (C 34.6 and H 1.68%) of parent UiO-66(Zr), suggesting the removal of most templates during the acid treatment. Moreover, again there is a little Zn in generated H-UiO-66(Zr), probably due to the same reason as proposed above. On the other hand, HAADF-STEM image and EDX mapping also show that Zr, C and O elements were homogeneously distributed in the whole template@H-UiO-66(Zr) but Zn element was heterogeneous (Supplementary Fig. 41).

To further explore the components of the molecule-based MOA templates in fabricating H-UiO-66(Zr) and the template removability, we prepared the H-UiO-66(Zr) by using Zn_4_O(BC-CH_3_)_6_ (BC-CH_3_, 4-methylbenzenecarboxylate) template precursor. As shown in Supplementary Fig. 38, based on the chemical shift at 20 p.p.m. the ^13^C-NMR spectrum indicates the existence of the BC-CH_3_ in template@H-UiO-66(Zr). After the acid treatment, this peak disappeared, indicating the removal of the template (Supplementary Fig. 39). Similarly, we also prepared the H-UiO-66(Zr) by using Zn_4_O(BC-NO_2_)_6_ (BC-NO_2_, 4-nitrobenzoic acid). As shown in Supplementary Fig. 32, based on the wavenumber at 1,522 and 1,437 cm^–1^, the FT-IR spectrum indicates the existence of the BC-NO_2_ in template@H-UiO-66(Zr). However, after the acid treatment these peaks disappeared, indicating the removal of the template. In terms of the above analysis, it was demonstrated that both metal ions and organic portions in template can be efficiently removed by the acid treatment, making sure the purity of resulting H-UiO-66(Zr).

In the case of MOP-*t*Bu (Fig. 2b)^44^, experimentally, as-synthesized MOP-*t*Bu, ZrCl_4_ and terephthalic acid were mixed and dispersed in DMF. The mixture solution was heated to address the reaction under the conditions similar to those for preparing parent UiO-66(Zr). Again, the resulting material has the same PXRD patterns as the UiO-66(Zr) and acid-treated sample exhibits the characteristic of the H-UiO-66(Zr) (Fig. 2e, Supplementary Figs 4,17,25 and 42). These results indicate that the MOPs can also act as the template precursors to construct H-MOFs.

Above proof-of-concept experiments reveal that MOAs with different original structural complexity and size, including primary complex molecule, supramolecular MOPs and structurally extended MOFs all can act as the template precursors for fabricating H-MOFs. Interestingly, the pore size of resulting H-MOFs is independent on the size of these initially used MOAs or their particles, suggesting that there exists a ‘decomposition and/or rearrangement' of them to form new MOAs, which act as the *in situ* self-assembly templates in the construction of these H-MOFs in given reaction systems.

### Preparation of H-MOFs through one-pot reaction

On the base of above interesting findings, we also tried to prepare H-MOFs by a one-pot reaction approach, so as to further confirm the *in situ* self-assembly template mechanism, again on H-UiO-66(Zr). One-pot reaction of Zn(NO_3_)_2_·6H_2_O, excess terephthalic acid and ZrCl_4_ in DMF was conducted under solvothermal conditions similar to those for the synthesis of parent UiO-66(Zr). After acid washing, PXRD and N_2_ adsorption confirm that the resulting material is H-UiO-66(Zr) (Supplementary Figs 12a and 22a). With the same route, we also checked the applicability of this strategy in using Zn_4_O(BC)_6_ and MOP-*t*Bu precursors as template sources in one-pot reaction. The product based on Zn_4_O(BC)_6_ precursors was obtained from the reaction of Zn(NO_3_)_2_·6H_2_O, benzoic acid, ZrCl_4_ and terephthalic acid in DMF. After washing with acid, H-UiO-66(Zr) was obtained as confirmed by the PXRD and N_2_ adsorption (Supplementary Figs 10a and 20a). It should be pointed out that the ligand exchange between formed MOFs and free ligands could exist in the solution of this system such as observed in UiO-66, Materials of Institute Lavoisier (MILs) and zeolitic imidazolate frameworks (ZIFs) systems^45^,^46^. Indeed, the UiO-66(Zr) with ligand missing-linker defects can also be prepared by adding given amount of modulators such as acetic acid, benzoic acid and trifluoroacetic acid in the reaction system^47^,^48^,^49^. However, the resulting new pores in these ‘defect' UiO-66(Zr) was small and no obvious hysteresis loop was observed in their N_2_ adsorption–desorption isotherms. In addition, we also tried to prepare H-UiO-66(Zr) with only adding HBC in the reaction system through the one-pot reaction between terephthalic acid and ZrCl_4_. N_2_ adsorption indicates that no mesopore was generated in resulting material (Supplementary Fig. 7). Similarly, H-UiO-66(Zr) can also be prepared through the reaction of Cu(NO_3_)_2_·3H_2_O, 5-*t*-butyl-1,3-benzenedicarboxylic acid, ZrCl_4_ and terephthalic acid in DMF, followed by acid washing (Supplementary Figs 11a and 21a). All these results demonstrate that a one-pot reaction of MOA template precursors and targeted H-MOF precursors is also feasible in the preparation of H-MOFs. As we expected, the ‘formation and degradation/rearrangement' of the MOAs in the *in situ* one-pot reaction enable them to play a template role in the formation of mesoporous structures of resulting H-MOFs.

To examine the universality of this *in situ* self-assembly method, we performed additional synthesis experiments on a couple of other typical MOFs with good physicochemical stabilities, including ZIF-8, MIL-101(Cr), DUT-5 and several functionalized UiO-66(Zr) by one-pot reaction. As expected, 19 H-MOFs were successfully prepared and characterized by EA, ICP, PXRD, FT-IR, TGA and N_2_ adsorption (see Supplementary Information). Some structural features of eight representative H-MOFs are listed in Table 1 and the complete data are provided in Supplementary Table 1.

As a whole, it was found that in the preparation, actual templates can not be identified in all cases. Combining element analysis and infrared characterizations, we found that almost no organic residues from the template or free ligands were left in pores of resulting H-MOFs after the acid treatment. It was also demonstrated that the crystallinity of all H-MOFs become to be bad compared with their parent MOFs, probably due to generating numerous defects in the structures of H-MOFs. The lost of crystallinity of H-MOFs in some cases has also been confirmed in literatures^28^,^32^. Furthermore, the crystallinity and the porosity are directly related to the template amounts as discussed in detail below. These H-MOFs have also a better purity, even if incomparable with their parent MOFs in some cases. For TGA results, some as-synthesized samples present several weight-loss steps, which could be basically ascribed to (1) the loss of a small quantity of free guest solvent molecules in pores of H-MOFs at lower temperature range, (2) the lose of high boiling point solvent molecules and coordinated water molecules in the H-MOFs structures at higher temperature range, and (3) the decomposition of the H-MOFs frameworks. The second step usually was combined by the third, representing a sequential weight loss in a broad temperature range. It was also found that TGA curves of some functional H-MOFs, for example, H-UiO-66-NH_2_(Zr), exhibited a continuous weight loss similar to its parent UiO-66-NH_2_(Zr) at the whole temperature range, which might be ascribed to the introduction of polar group in these MOFs (Supplementary Fig. 30)^38^.

In addition, we also noticed that the template not only can create mesopore for H-MOFs but also can affect the microporosity of the MOFs (Table 1). For example, the micropore surface area of H-UiO-66(Zr) (prepared by using Zn_4_O(BC)_6_ precursors as the template source) is 300 m^2^ g^−1^, which is much smaller than that of parent UiO-66(Zr) (1,024  m^2^ g^−1^), whereas the micropore size is 11.8 and 14.1 Å, being larger than those in UiO-66(Zr) (8 Å and 11 Å, corresponding to the tetrahedral and octahedral cages, respectively). For other H-MOFs, the similar results are observed. That is, the introduction of templates can decrease the micropore surface area and increase the micropore diameter at different degrees of final H-MOFs. In particular, for the H-MIL-101(Cr), there should have been various mesopores with the sizes across the two mesoporous cages (29 and 34 Å) in parent MIL-101(Cr). As a result, no step adsorption similar to that in MIL-101(Cr) was observed in H-MIL-101(Cr) (Supplementary Fig. 12j).

**Figure 3:**
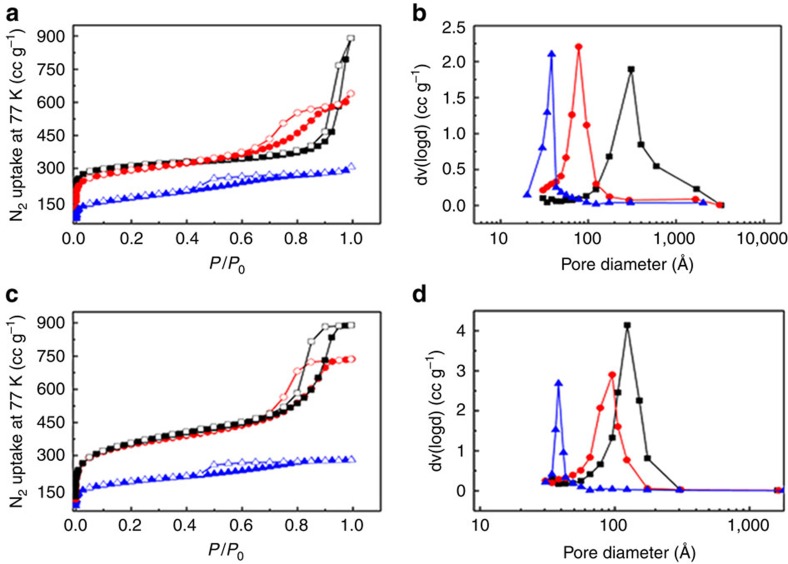
Tuning the mesopore size through changing the amount of template. (**a**,**c**) N_2_ adsorption–desorption isotherms at 77 K and (**b**,**d**) pore size distributions of H-UiO-66(Zr) prepared with different amounts of template (preparation conditions: (**a**,**b**) 1 ml (black curve), 2 ml (red curve) and 3 ml (blue curve) of nanosized MOF-5 suspension solution in DMF as the template precursor (the MOF-5 concentration in the suspension solution is about 12 mg ml^–1^); (**c**,**d**) 0.25 equiv. (black curve), 0.375 equiv. (red curve) and 0.5 equiv. (blue curve) of Zn_4_O(BC)_6_ template precursor (equiv. means the equivalent of Zn_4_O(BC)_6_ with respect to ZrCl_4_)).

It should be pointed out that the development of reliable methods to rationally prepare H-MOFs with controllable/tailorable structures and properties is much more challenged. In this work, we have definitely confirmed that the mesopore sizes of the resulting H-MOFs are tunable by varying the amount of the MOAs templates. Taking H-UiO-66(Zr) as an example, Fig. 3 shows that the mesopore diameters of prepared materials can vary from 40 to 300 Å, depending on the amount of Zn_4_O(BC)_6_ or MOF-5 template precursors used in synthesis. It must be pointed out here that based on our experimental results, only when the amounts of Zn_4_O(BC)_6_ template precursors were controlled in the range of 0.25∼0.75 equiv., the decrease of template precursor amounts can increase the mesopore sizes in the resulting H-UiO-66(Zr). As shown in Supplementary Figs 9 and 23, the H-UiO-66(Zr) became of poor crystallinity and even amorphous if the template precursors were more than 0.75 equiv., while no mesopore structure was created when less than 0.25 equiv. This observed relationship between mesopore size and the amount of template precursor may be related to the size of the formed template fragments under different precursor concentration conditions. In general, in crystallization process, a lower concentration of the precursor will lead to a lower nucleation rate, which results in a lower concentration of nuclei in the reaction system. A limited number of nuclei grow slowly in the reaction system, consequently resulting in bigger crystal particles. Otherwise, a higher concentration of the precursor can lead to a higher nucleation rate and much more nuclei, thus smaller crystal particles can be expected at a higher concentration of the precursor. As a consequence, an increase of the concentration (amount) of precursor in the reaction mixture usually resulted in a decrease of the generated template particle size in a given concentration range^50^. To further verify the tunability of mesoporosity of H-MOFs prepared by this approach, the mesopore sizes of H-ZIF-8, H-MIL-101(Cr) and H-DUT-5 synthesized with different amounts of template precursor were also tested (Supplementary Figs 13–15). It can be seen clearly from these results that the mesopore sizes of these H-MOFs are dependent on the amount of template precursors used in each case.

As stated above, the ligand-extension strategy for expanding pore size of MOFs often induces the interpenetration and instability of the MOF frameworks. Nevertheless, using our *in situ* self-assembly template method that takes the advantage of relative stability of different MOAs, stable mesoporous structures of H-MOFs can be easily created. Furthermore, the resulting H-MOFs with tunable mesopore sizes are stable just similar to their parents, as confirmed in the case of H-UiO-66(Zr) and UiO-66(Zr) (Supplementary Fig. 43).

### Large-molecule adsorption in H-MOFs

**Figure 4:**
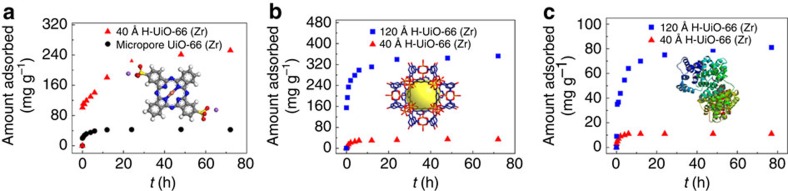
Large-molecule adsorption in H-UiO-66(Zr). (**a**) Adsorption kinetics of dye DB 86 in microporous UiO-66(Zr) and 40 Å (means mesopore size in the H-MOF) H-UiO-66(Zr), (**b**) adsorption kinetics of MOP-OH in 40 and 120 Å H-UiO-66(Zr), and (**c**) adsorption kinetics of BSA in 40 and 120 Å H-UiO-66(Zr).

We know that adsorbents with large pores could be used in the capture of large molecules. Here, taking H-UiO-66(Zr) as a representative of prepared H-MOFs in this work, we performed liquid-phase adsorption experiments to explore the accessibility of their mesopores towards large molecules with different sizes. For this purpose, organic dye molecule (Direct Blue 86, DB 86, about 4 × 12 × 14 Å in size), MOPs (MOP-OH, about 40 × 40 × 40 Å in size) and biological protein molecule (bovine serum albumin (BSA), about 140 × 40 × 40 Å in size) were selected as the probe molecules. As shown in Fig. 4, microporous UiO-66(Zr) almost can not adsorb DB 86 molecules from aqueous solution, but H-UiO-66(Zr) with about 40 Å mesopores can efficiently capture this large molecule. With regard to recognizing different mesopore sizes of H-HiO-66(Zr), larger MOP-OH and BSA were further checked. It was found that the H-UiO-66(Zr) with 40 Å mesopores can hardly accommodate the two types of molecules, but the one with 120 Å mesopores can. Although the length of BAS is a little bit longer than the mesopore size of the H-UiO-66(Zr), this material can still accommodate the protein, which can be attributed to the slender geometry of BAS and the irregular shape of mesopore in it. In addition, white H-UiO-66(Zr) powder exhibited a colour change after DB 86 or MOP-OH adsorption as shown in Supplementary Fig. 44. Clearly, these stable H-MOFs with easily tuned mesopores can find potential applications in large-molecule adsorption/separation.

## Discussion

Foregoing results demonstrate that using an *in situ* self-assembly template method, by taking advantage of relative stability of different metal-organic coordination assemblies, stable H-MOFs with tailorable pore sizes can be easily prepared based on their stable parent microporous MOFs. Although the exact nature of the template can not be identified at this stage, this approach is quite feasible and seems to be versatile in preparing H-MOFs. Such types of hierarchical-pore materials may provide promising applications in large-molecule adsorption/separation, heterogeneous catalysis and drug release due to their several distinct merits, such as having co-existed micropores and mesopores, large pore size, good stability and improved mass transfer in their pores.

## Methods

### Synthesis

Experimental procedures for preparing all the H-MOFs achieved in this work are provided in the Supplementary Information. Here we take H-UiO-66(Zr) preparation with MOF-5 precursor as the template source, as an example to describe the preparation method. The synthesis started by mixing parent MOF precursors and template precursors, including 0.120 g of ZrCl_4_, 0.160 g of H_2_BDC, 0.297 g of Zn(NO_3_)_2_·6H_2_O, with 20 ml of DMF in a 100-ml Teflon liner. After sonication for 10 min the Teflon liner vessel was sealed and placed in a preheated oven at 120 °C for 24 h. After cooling to room temperature, the resulted powder was separated by centrifugation. The supernatant was discarded and the solid was washed several times with DMF to remove the unreacted precursors, to give as-prepared template@H-UiO-66(Zr). To remove the acid-sensitive MOA templates, the solid was dispersed in 10 ml of diluted HCl solution (pH 1) and stirred for about 10 min. After discarding the supernatant, the obtained solid was re-dispersed in DMF and then centrifuged. This process was repeated three times so that all decomposed template fragments were removed. Next, a similar process was performed by using acetone as the solvent, instead of DMF, afterwhich the solid was heated at 150 °C for 12 h at vacuum, to get activated sample for N_2_ adsorption measurement.

### Characterization

The characterizations of MOF-5 particle size, EA, ICP, SEM, transmission electron microscope, N_2_ adsorption–desorption isotherms at 77 K, PXRD, TGA, FT-IR, XPS and NMR spectra are given in Supplementary Information. Particle size distributions and SEM micrograph of MOF-5 particles are shown in Supplementary Figs 1 and 2. N_2_ adsorption–desorption isotherms and pore size distributions of H-MOFs are included in Supplementary Figs 3–15. PXRD patterns of H-MOFs are given in Supplementary Figs 16–23. TGA curves of H-MOFs are given in Supplementary Figs 24–30. FT-IR spectra of H-MOFs are given in Supplementary Figs 31–35. XPS spectra of H-MOFs are included in Supplementary Figs 36 and 37. ^13^C-NMR spectra of H-UiO-66(Zr) are given in Supplementary Figs 38 and 39. HAADF-STEM image and corresponding elemental maps in template@H-UiO-66(Zr) are shown in Supplementary Figs 40–42. PXRD patterns of H-UiO-66(Zr) before and after soaking in water for 24 h are given in Supplementary Fig. 43. Photographs of H-UiO-66(Zr) before and after large molecular adsorption are shown in Supplementary Fig. 44. Porosity properties of various H-MOFs and their parent MOFs are included in Supplementary Table 1. Element mole ratios in template@H-MOFs and H-MOFs are given in Supplementary Table 2.

## Additional information

**How to cite this article:** Huang, H. *et al*. An *in situ* self-assembly template strategy for the preparation of hierarchical-pore MOFs. *Nat. Commun.* 6:8847 doi: 10.1038/ncomms9847 (2015).

## Supplementary Material

Supplementary Information Supplementary Figures 1-44, Supplementary Tables 1-2, Supplementary Methods and Supplementary References

## Figures and Tables

**Figure 1:**
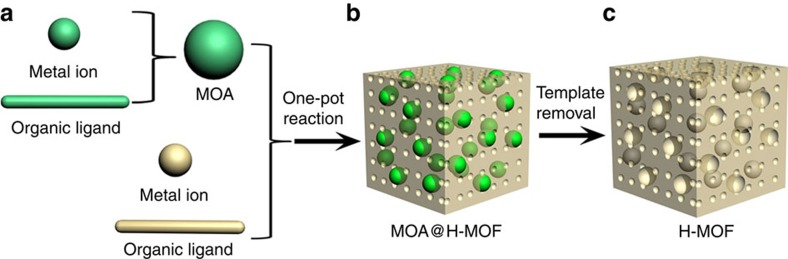
Schematic representation for the preparation of H-MOF. (**a**) *In situ* self-assembly of MOA through the reaction between metal ion and organic ligand. (**b**) MOA@H-MOF composite formed by one-pot self-assembly reaction. (**c**) H-MOF formed through removing MOA template.

**Table 1:** Pore features of eight representative H-MOFs (prepared by one-pot reaction) and their parent MOFs.

H-MOF and MOF	MOA template precursor type	*S*_BET_* (m^2^ g^−1^)	*S*_micro_† (m^2^ g^−1^)	*S*_micro_/*S*_meso_‡	*V*_t_§ (m^2^ g^−1^)	*V*_micro_|| (m^2^ g^−1^)	*V*_micro_/*V*_meso_¶	Micro pore size (Å)#	Meso pore size** (Å)
H-ZIF-8	MOF-5	1,611	1,117	2.26	0.82	0.29	0.55	12.7	38.5
ZIF-8		1,737	1,737		0.7			11	
H-MIL-101(Cr)	ZIF-8	441	157	0.55	0.58	0.07	0.13	51	123
MIL-101(Cr)		2,927	2,357		1.49			29/34	
H-DUT-5	In-BPDC	1,183	737	1.65	0.91	0.68	2.96	11.0	39.4
DUT-5		1,652	1,424		0.82			11.1	
H-UiO-66(Zr)	Zn_4_O(BC)_6_	917	300	0.49	0.87	0.13	0.17	11.8	38
UiO-66(Zr)		1,204	1,024		0.59			8/11	
H-UiO-66-NH_2_(Zr)	IRMOF-3	600	327	1.19	0.49	0.14	0.38	11.8/14.7	56.2
UiO-66-NH_2_(Zr)		1,070	1,052		0.42			7.4/9.5	
H-UiO-66-Cl(Zr)	Zn_4_O(BC)_6_	758	334	0.79	0.81	0.15	0.23	12	56.2
UiO-66-Cl(Zr)		794	750		0.33			5.8/7.5	
H-UiO-66-Br(Zr)	MOP-*t*Bu	558	221	0.66	0.50	0.11	0.29	11	55.5
UiO-66-Br(Zr)		806	615		0.43			5.6/7.3	
H-UiO-66(Hf)	MOP-*t*Bu	505	171	0.51	0.77	0.09	0.12	11.2	52.5
UiO-66(Hf)		890	739		0.43			8/11	

H-MOF, hierarchical-pore MOF; MOA, metal-organic assembly; MOF, metal-organic framework.

^*^S_BET_ is the Brunauer-Emmett-Teller (BET) specific surface area.

^†^*S*_micro_ is the *t*-plot-specific micropore surface area calculated from the N_2_ adsorption–desorption isotherm.

^‡^*S*_meso_ is the specific mesopore surface area estimated by subtracting *S*_micro_ from *S*_BET_.

^§^*V*_t_ is the total specific pore volume determined by using the adsorption branch of the N_2_ isotherm at *P/P*_0_=0.99.

^||^*V*_meso_ is the specific mesopore volume obtained from the Barrett-Joyner-Halenda (BJH) cumulative specific adsorption volume of pores of 1.70–300.00 nm in diameter.

^¶^*V*_micro_ is the specific micropore volume calculated by subtracting *V*_meso_ from *V*_t_.

^#^The micropore diameter is determined by the density functional theory (DFT) method.

^**^The mesopore diameter is determined from the local maximum of the BJH distribution of pore diameters obtained in the adsorption branch of the N_2_ isotherm at 77 K.
