# Supplementary material for: An in situ self-assembly template strategy for the preparation of hierarchical-pore metal-organic frameworks
Source: Nat Commun. 2015 Nov 9;6:8847. doi: 10.1038/ncomms9847 (PMC4667608; doi:10.1038/ncomms9847)
Supplement: Supplementary Information — Supplementary Figures 1-44, Supplementary Tables 1-2, Supplementary Methods and Supplementary References [file ncomms9847-s1.pdf]

Supplementary Figures

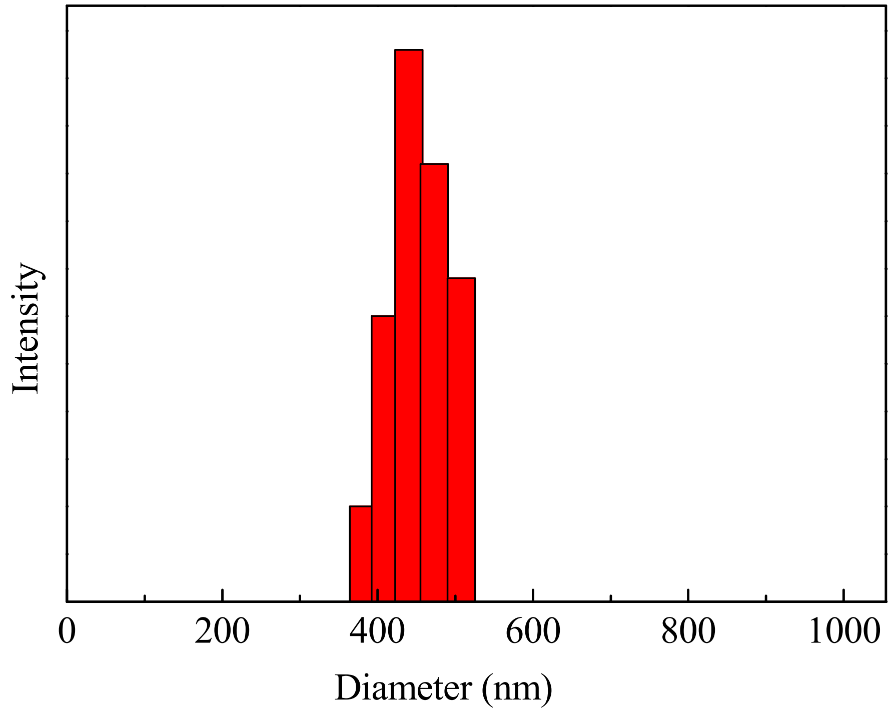

Supplementary Figure 1. Particle size distribution of used MOF-5 particles.

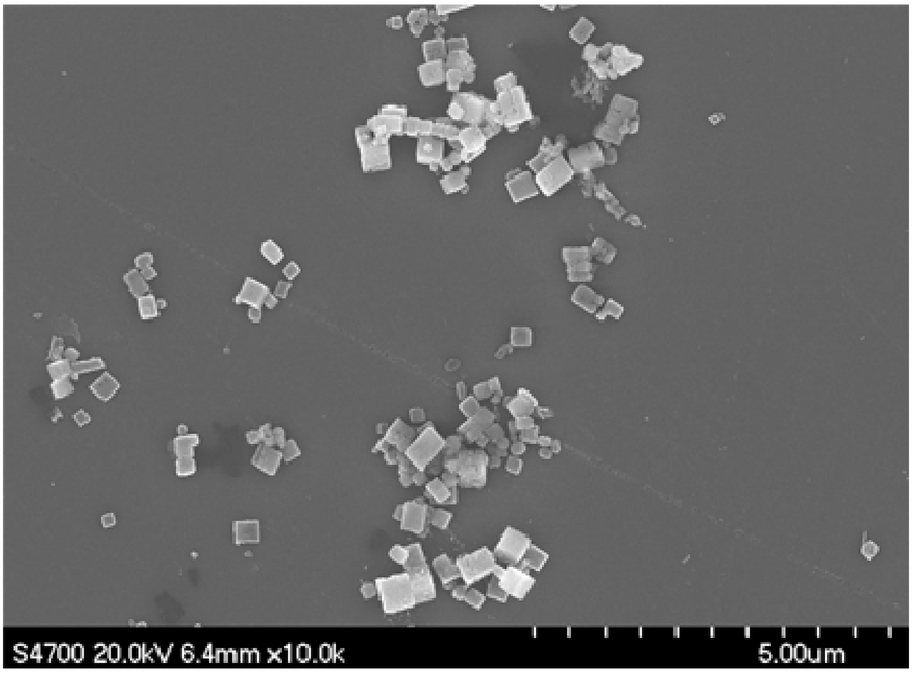

Supplementary Figure 2. SEM micrograph of MOF-5 crystal particles.

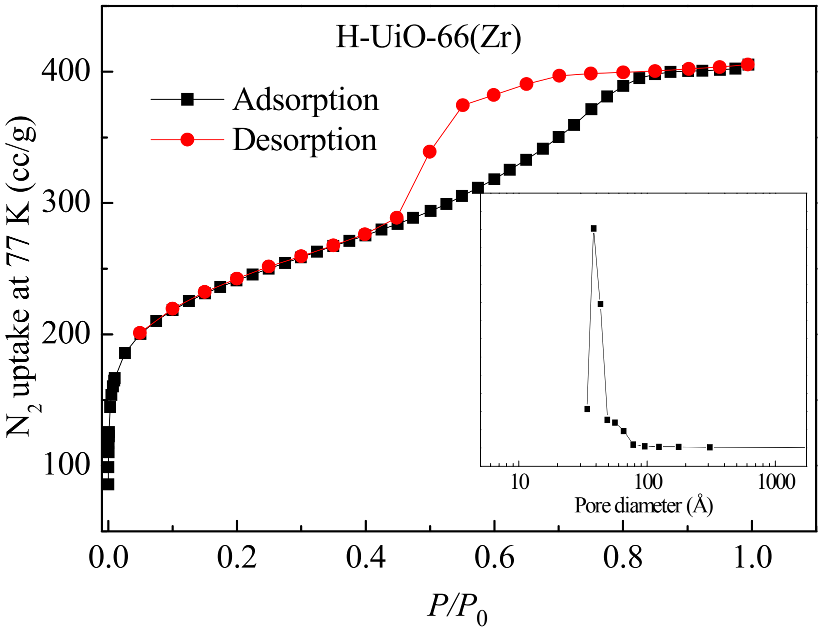

Supplementary Figure 3. N_2 adsorption-desorption isotherms at 77 K and pore size distributions of H- H-UiO-66(Zr) synthesized with $Zn_4O(BC)_6$ as template precursor by a two-step process.

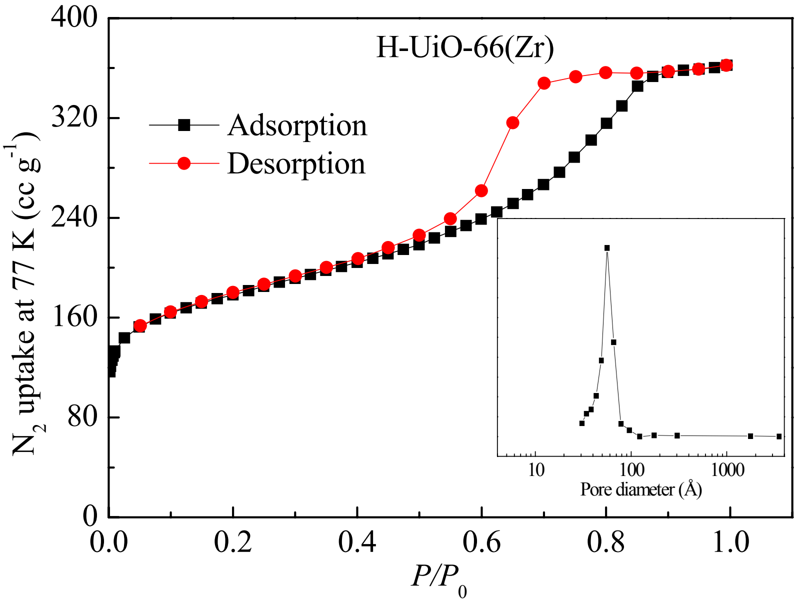

Supplementary Figure 4. N_2 adsorption-desorption isotherms at 77 K and pore size distributions of H-UiO-66(Zr) synthesized with MOP-*t*Bu as template precursor by a two-step process.

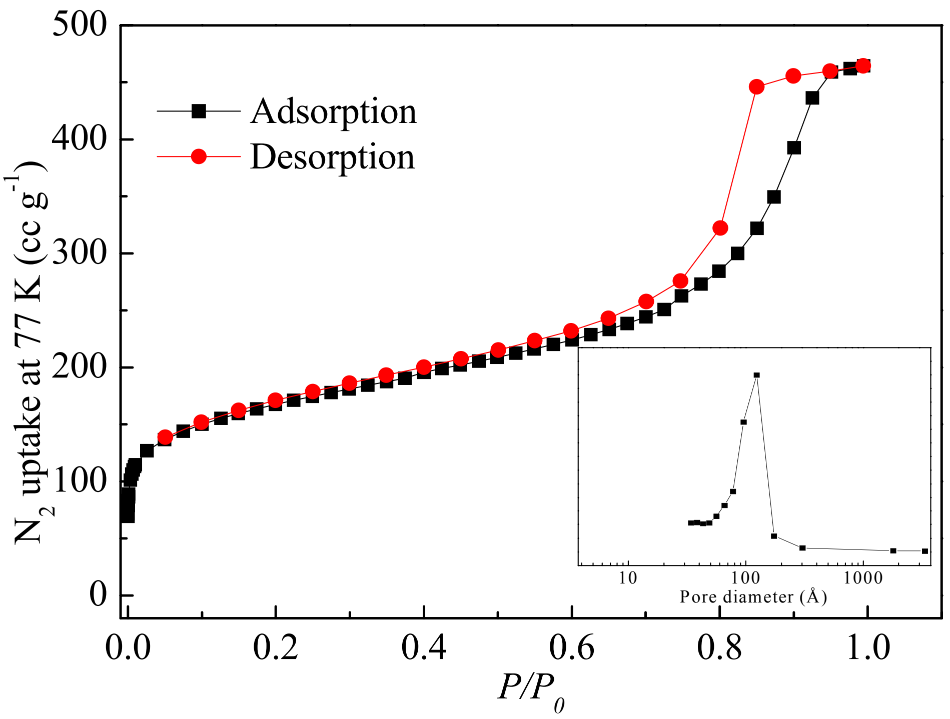

Supplementary Figure 5. N_2 adsorption-desorption isotherms at 77 K and pore size distributions of H-UiO-66(Zr) synthesized with MOF-5 as template precursor by a two-step process.

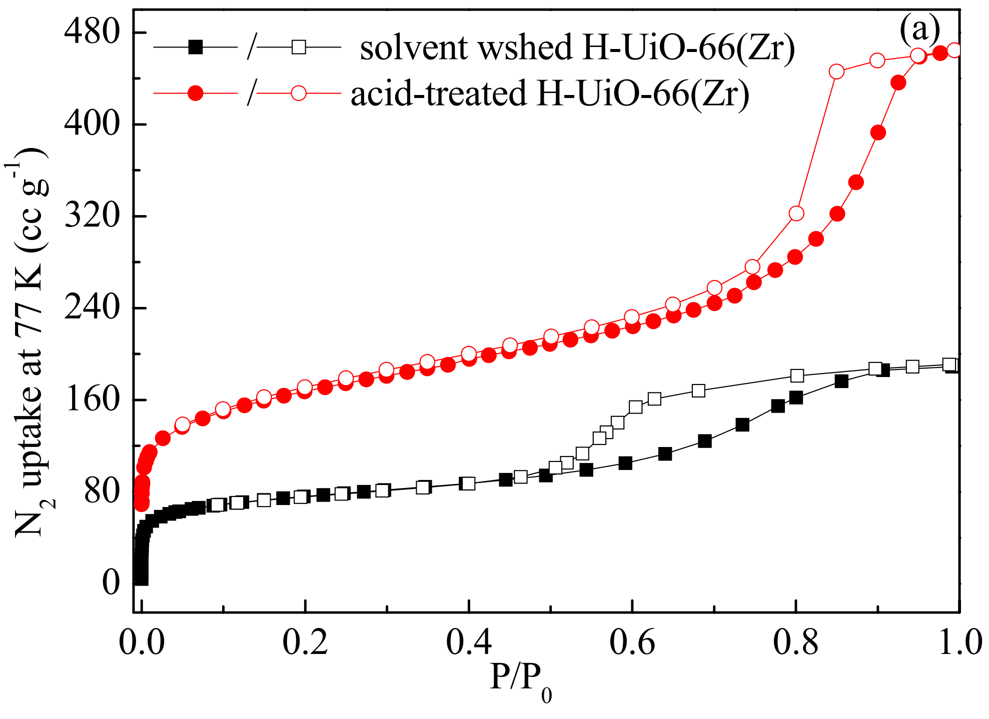

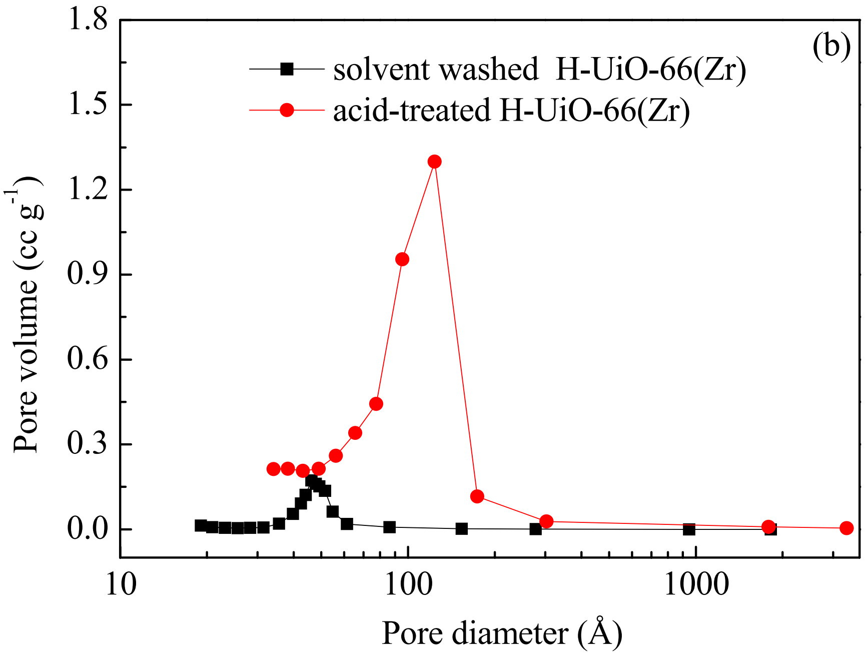

Supplementary Figure 6. N_2 adsorption-desorption isotherms at 77 K (**a**) and pore size distributions (**b**) of H-UiO-66(Zr) synthesized with MOF-5 as template precursor before and after acid treatment.

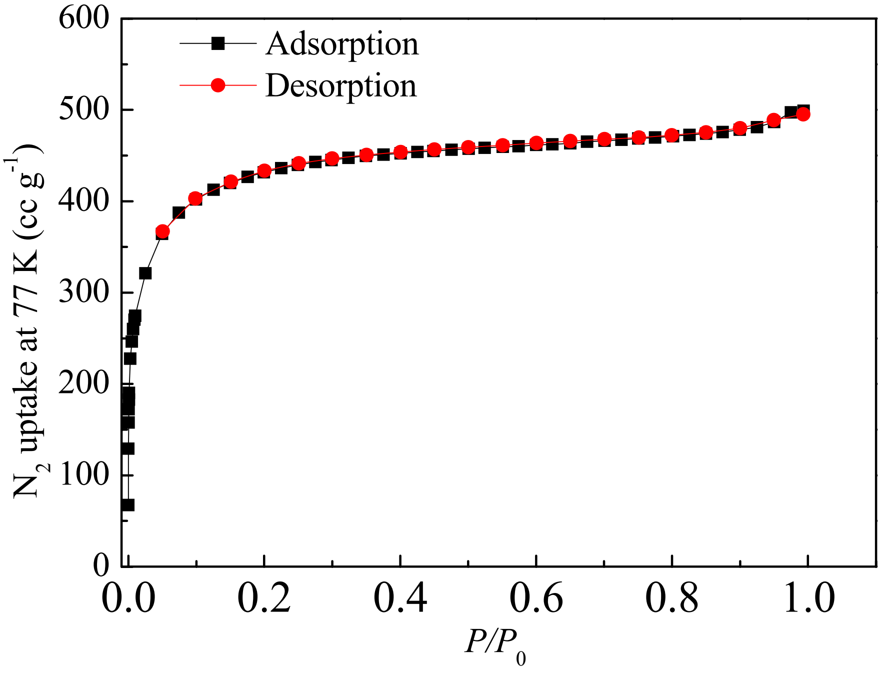

Supplementary Figure 7. N_2 adsorption-desorption isotherms at 77 K for the UiO-66(Zr) sample prepared through only adding HBC ligand in the reaction system of terephthalic acid with $ZrCl_4$.

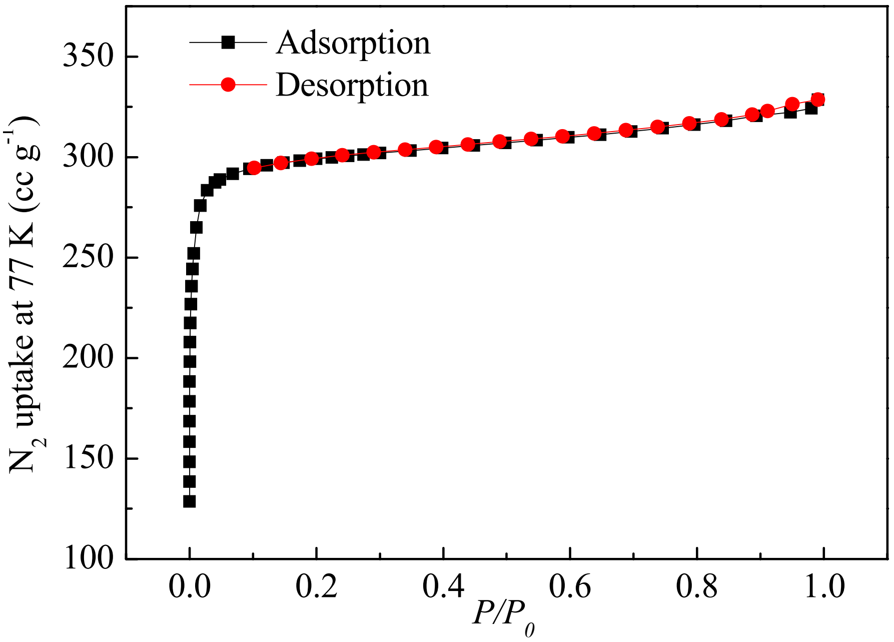

Supplementary Figure 8. N_2 adsorption-desorption isotherms at 77 K of UiO-66(Zr) after soaked in HCl solution (pH = 1) for 12 hours.

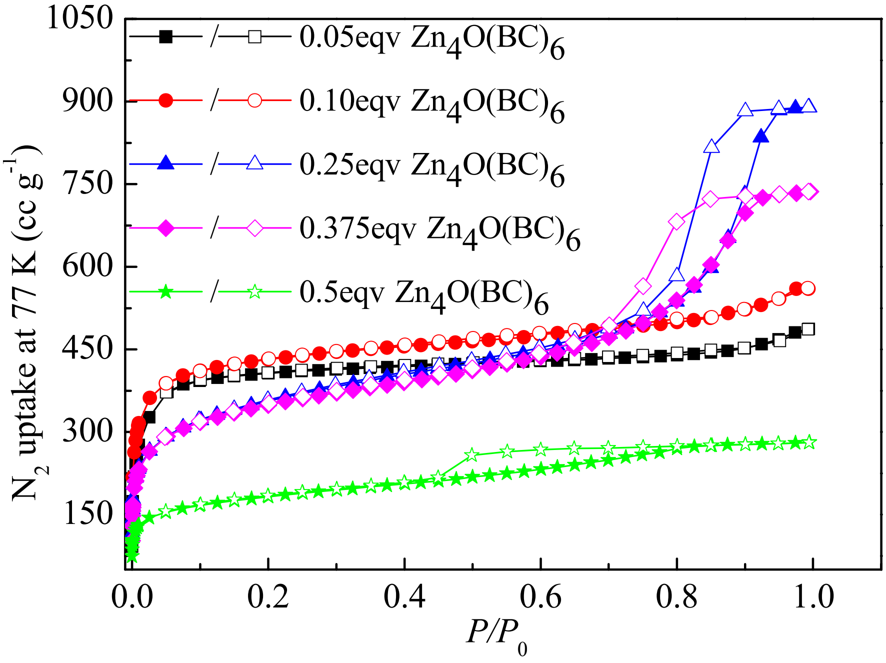

Supplementary Figure 9. N_2 adsorption-desorption isotherms at 77 K of UiO-66(Zr) prepared with different amounts of $Zn_4O(BC)_6$ precursors (eqv means the equivalent of $Zn_4O(BC)_6$ with respect to $ZrCl_4$).

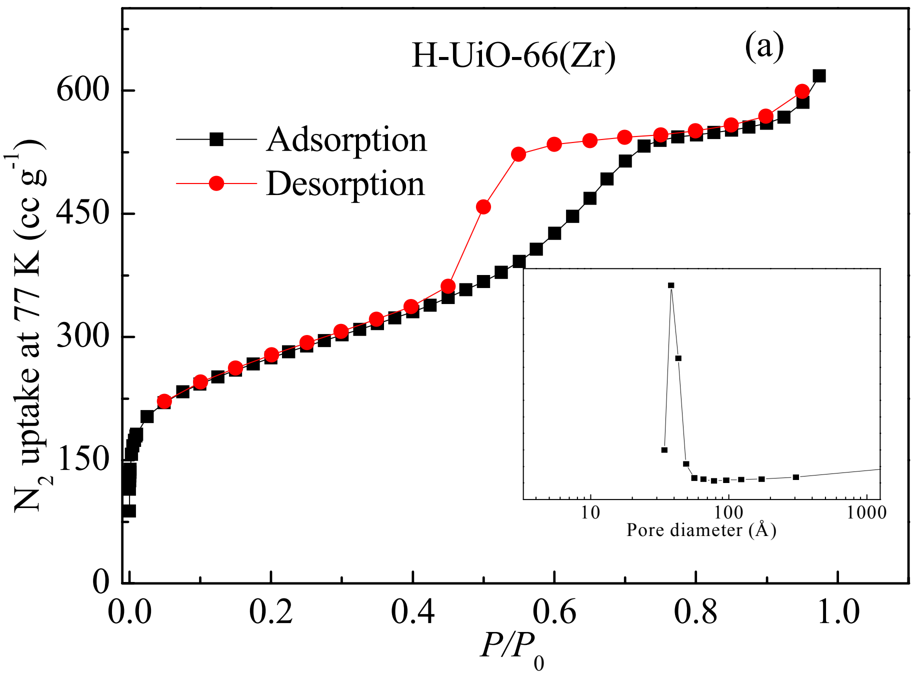
H-UiO-66(Zr)
(a)
Adsorption
Desorption
N2 uptake at 77 K (cc g-1)
P/P0
0
150
300
450
600
0.0
0.2
0.4
0.6
0.8
1.0
10
100
1000
Pore diameter (Å)

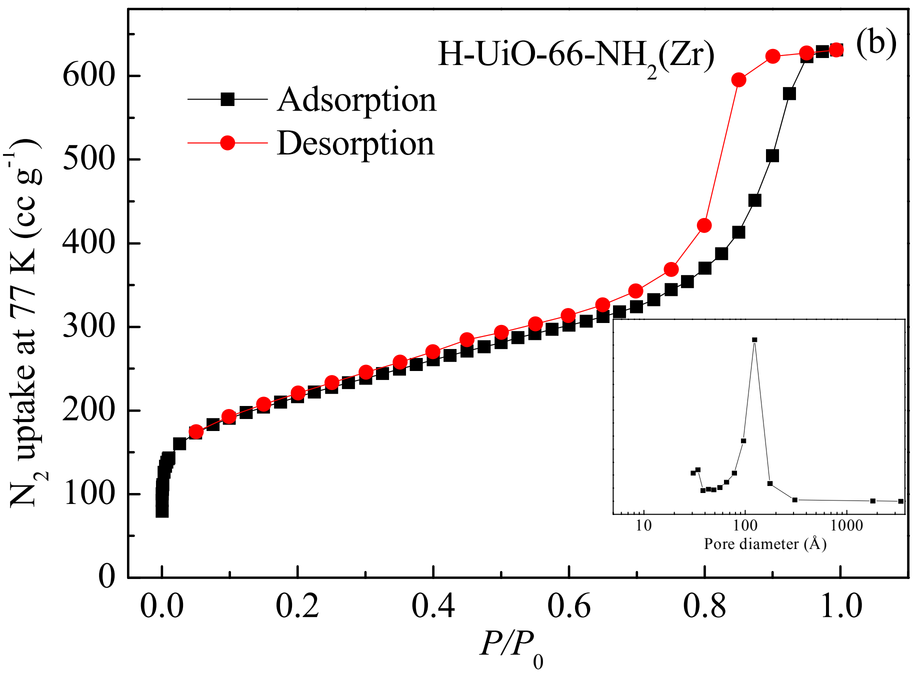
H-UiO-66-NH2(Zr)
(b)
Adsorption
Desorption
N2 uptake at 77 K (cc g-1)
P/P0
0
100
200
300
400
500
600
0.0
0.2
0.4
0.6
0.8
1.0
10
100
1000
Pore diameter (Å)

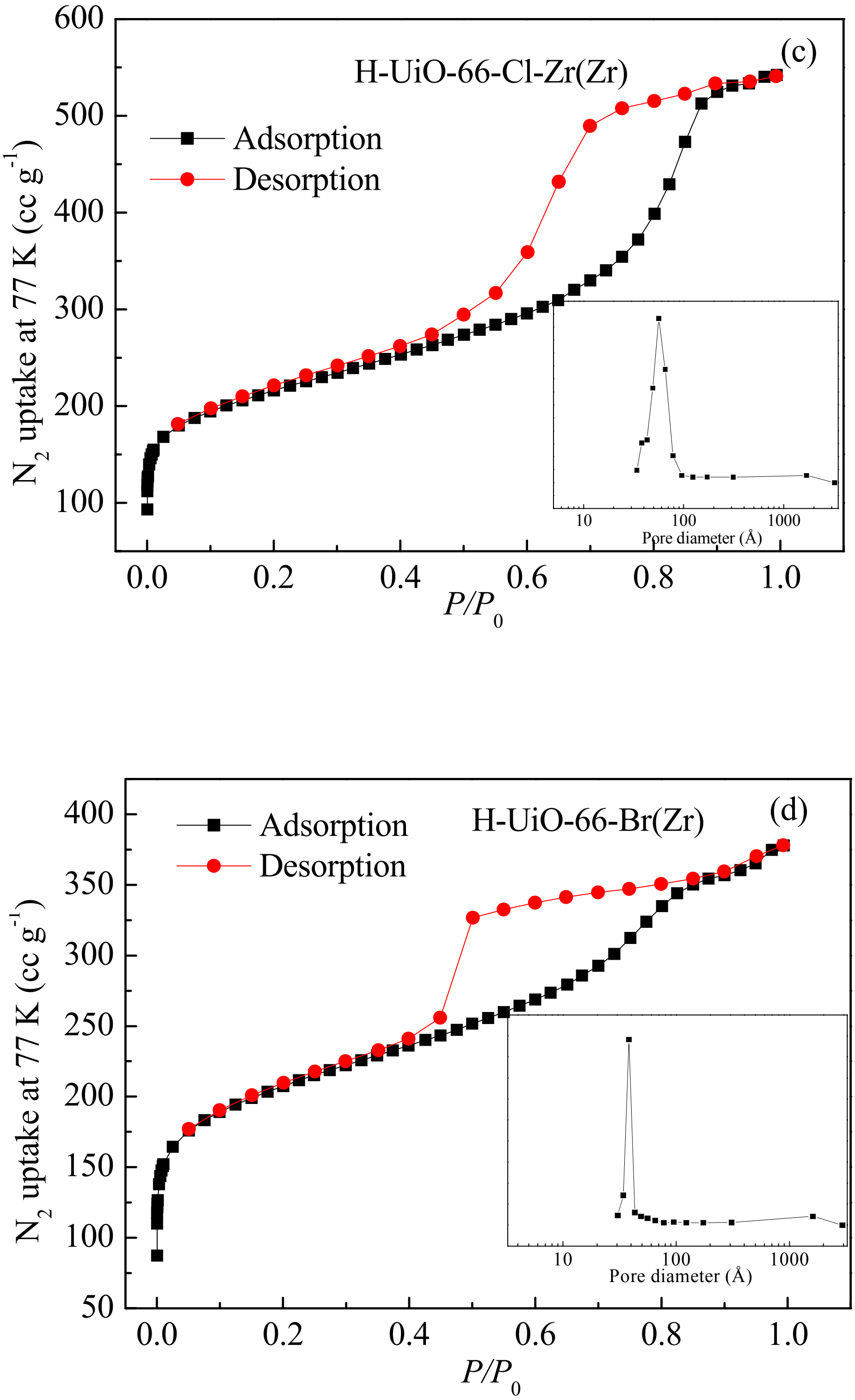

Supplementary Figure 10. N_2 adsorption-desorption isotherms at 77 K and pore size distributions of H-UiO-66-X(Zr) synthesized with $Zn_4O(BC)_6$ precursors template by one-pot reaction method.

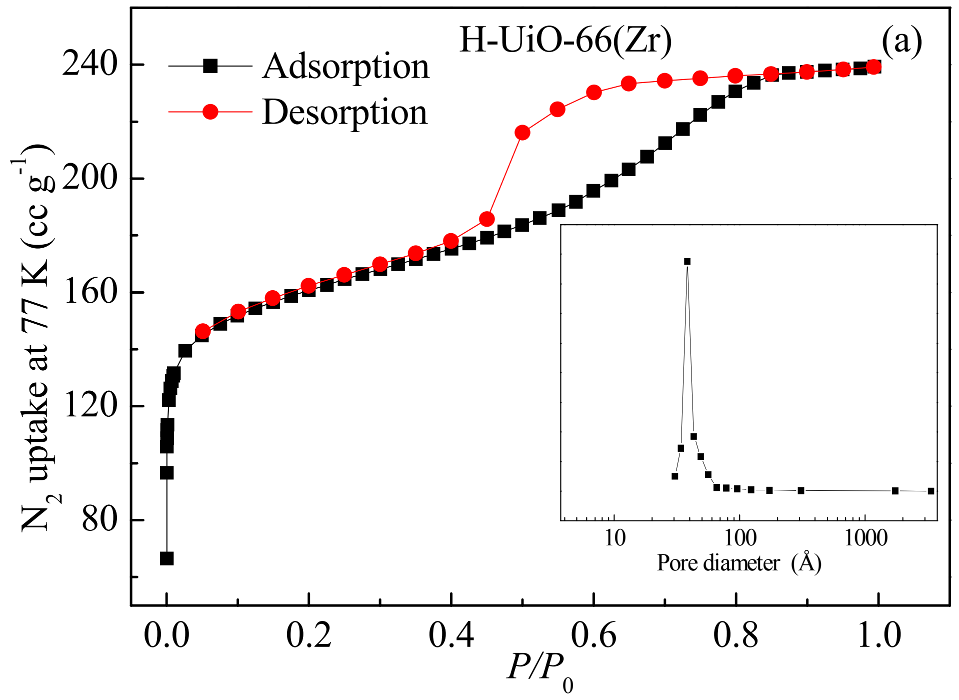
H-UiO-66(Zr)
(a)
Adsorption
Desorption
N2 uptake at 77 K (cc g-1)
P/P0
Pore diameter (Å)

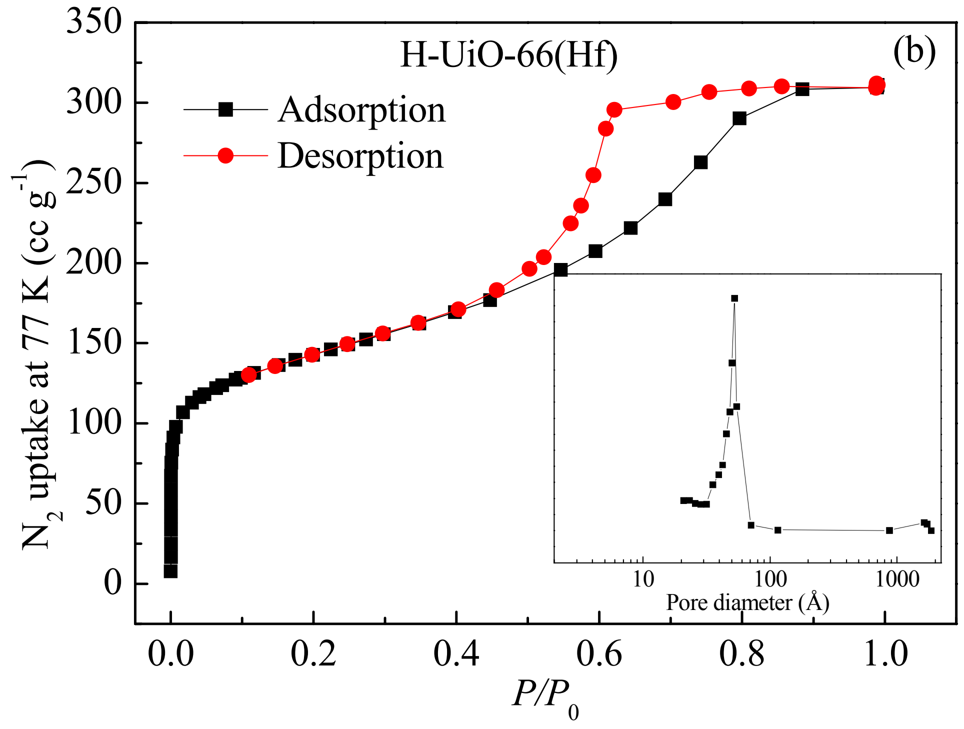
H-UiO-66(Hf)
(b)
Adsorption
Desorption
N2 uptake at 77 K (cc g-1)
P/P0
Pore diameter (Å)

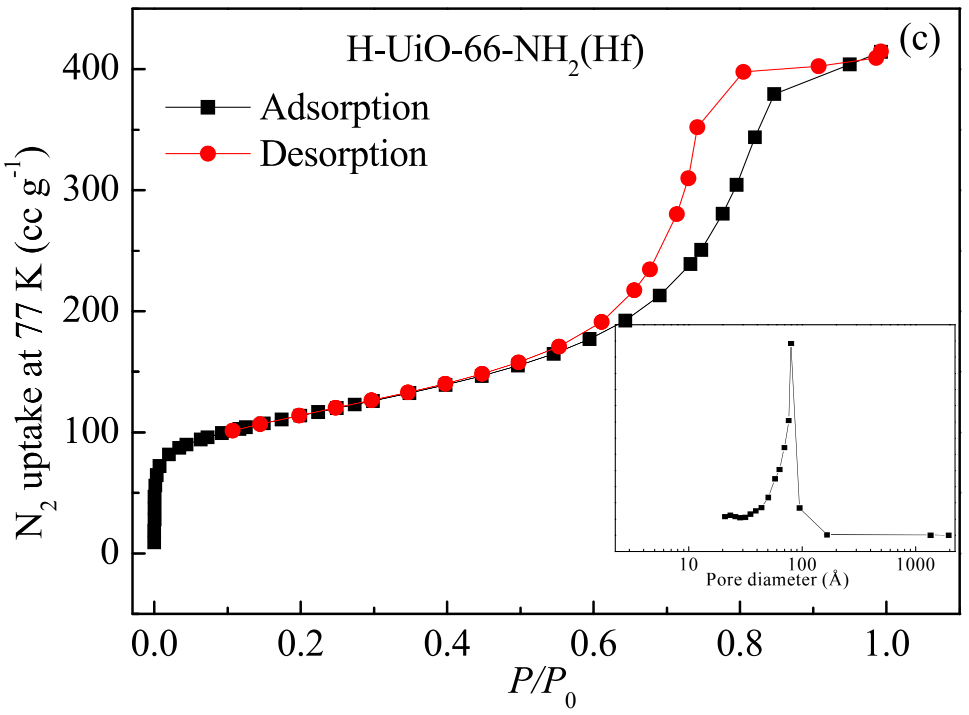

H-UiO-66-NH2(Hf)
(c)
Adsorption
Desorption
N2 uptake at 77 K (cc g-1)
P/P0
Pore diameter (Å)

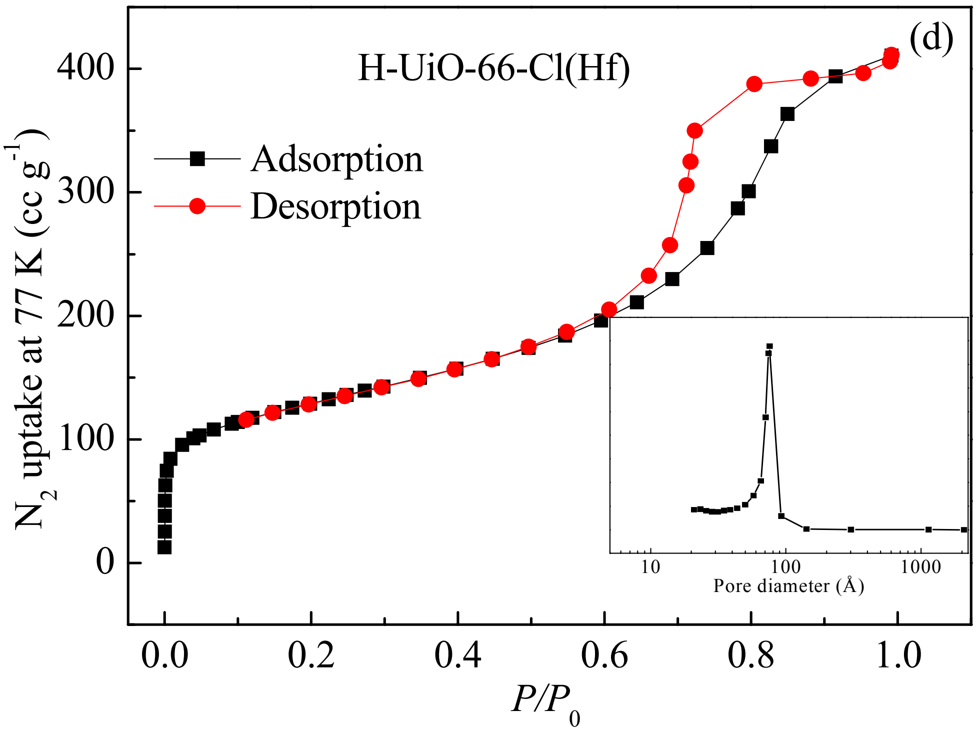

H-UiO-66-Cl(Hf)
(d)
Adsorption
Desorption
N2 uptake at 77 K (cc g-1)
P/P0
Pore diameter (Å)

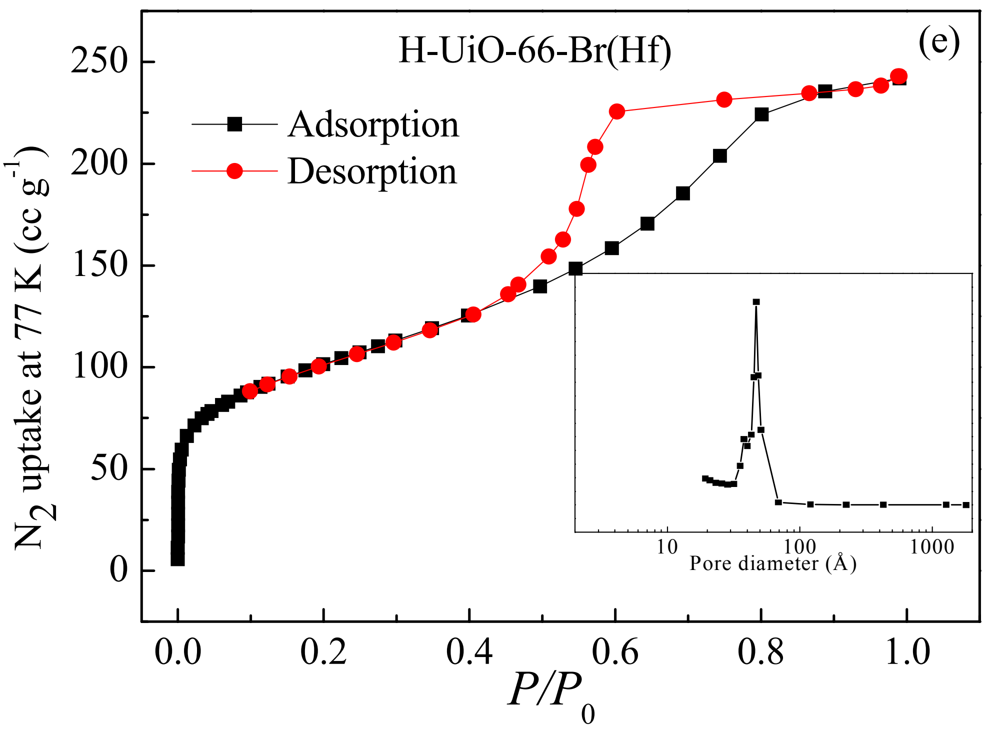

Supplementary Figure 11. N_2 adsorption-desorption isotherms at 77 K and pore size distributions of H-MOFs synthesized with MOP-*t*Bu precursors as template by one-pot reaction method.

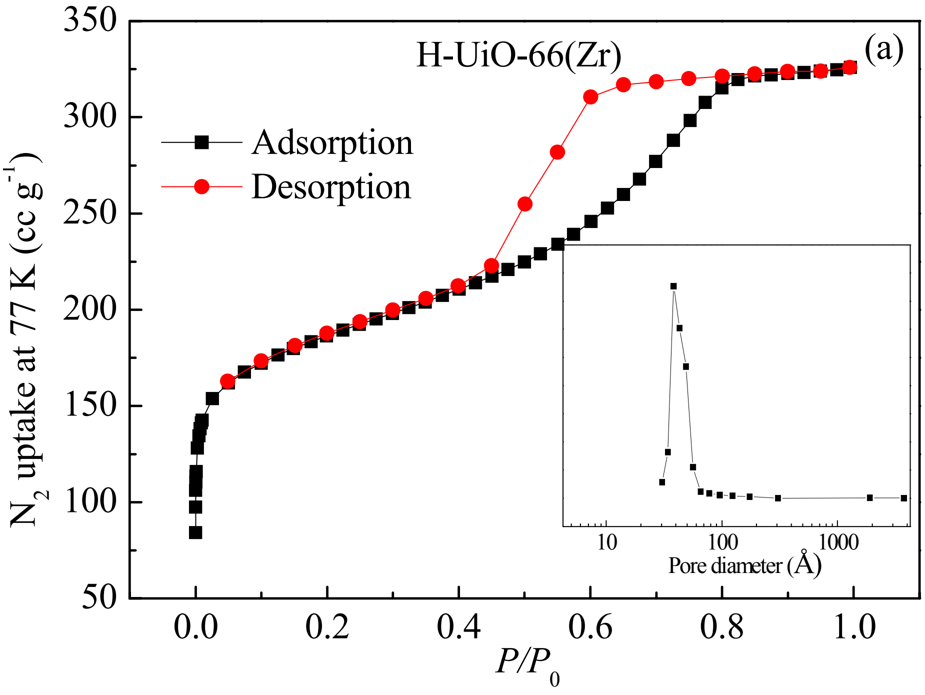

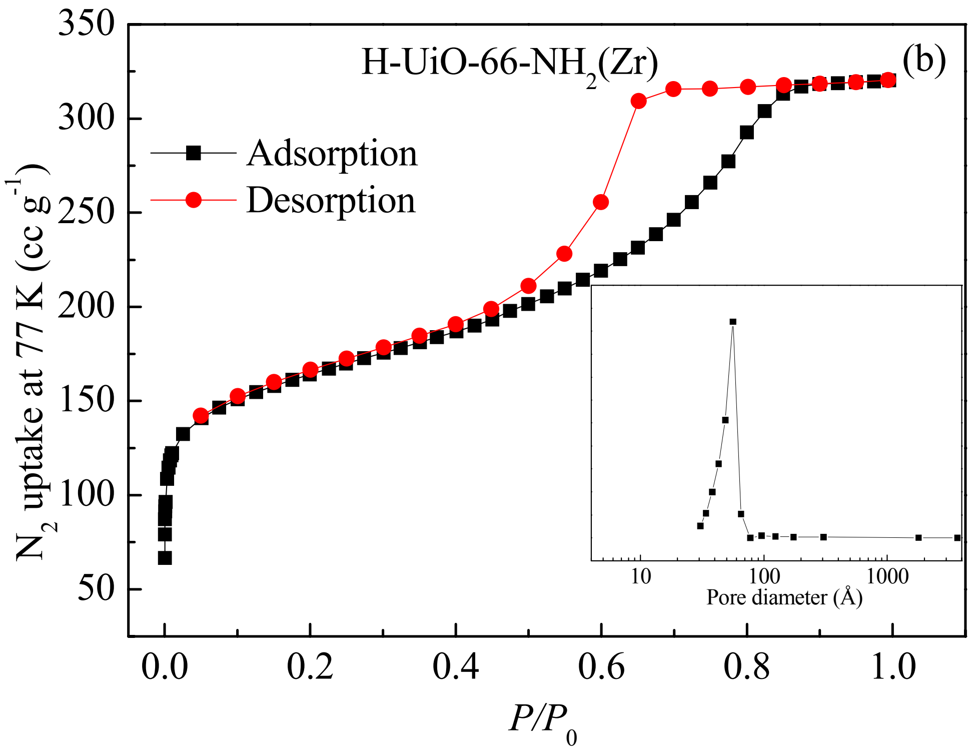
H-UiO-66-NH$_2$(Zr)
(b)
Adsorption
Desorption
N$_2$ uptake at 77 K (cc g^{-1})
P/P$_0$
Pore diameter (Å)

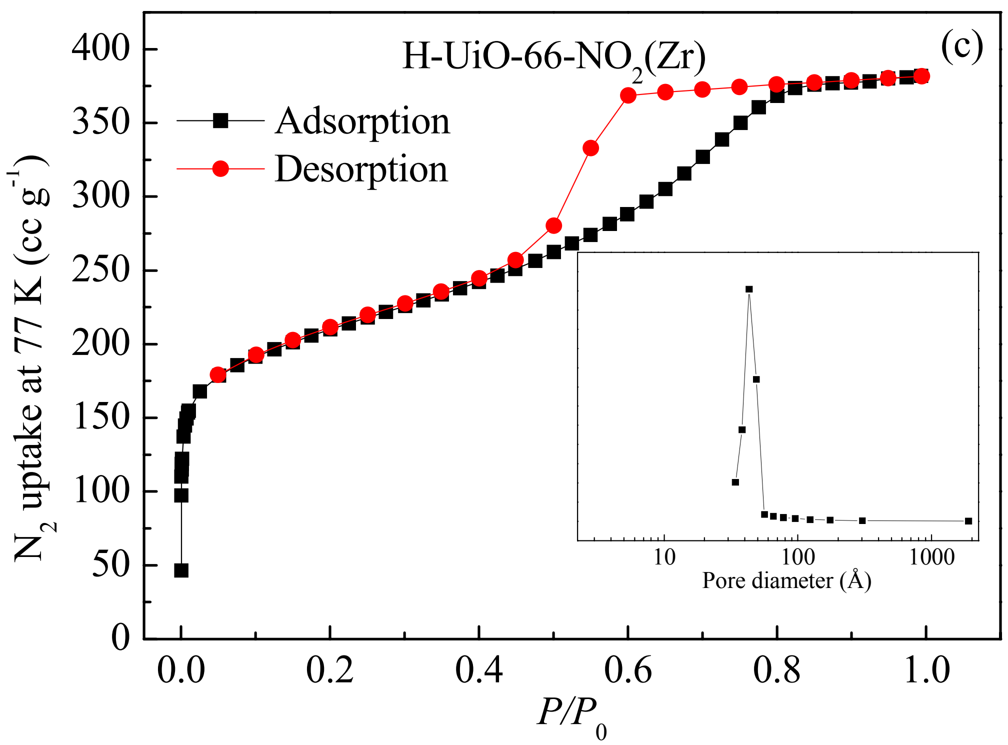
H-UiO-66-NO$_2$(Zr)
(c)
Adsorption
Desorption
N$_2$ uptake at 77 K (cc g^{-1})
P/P$_0$
Pore diameter (Å)

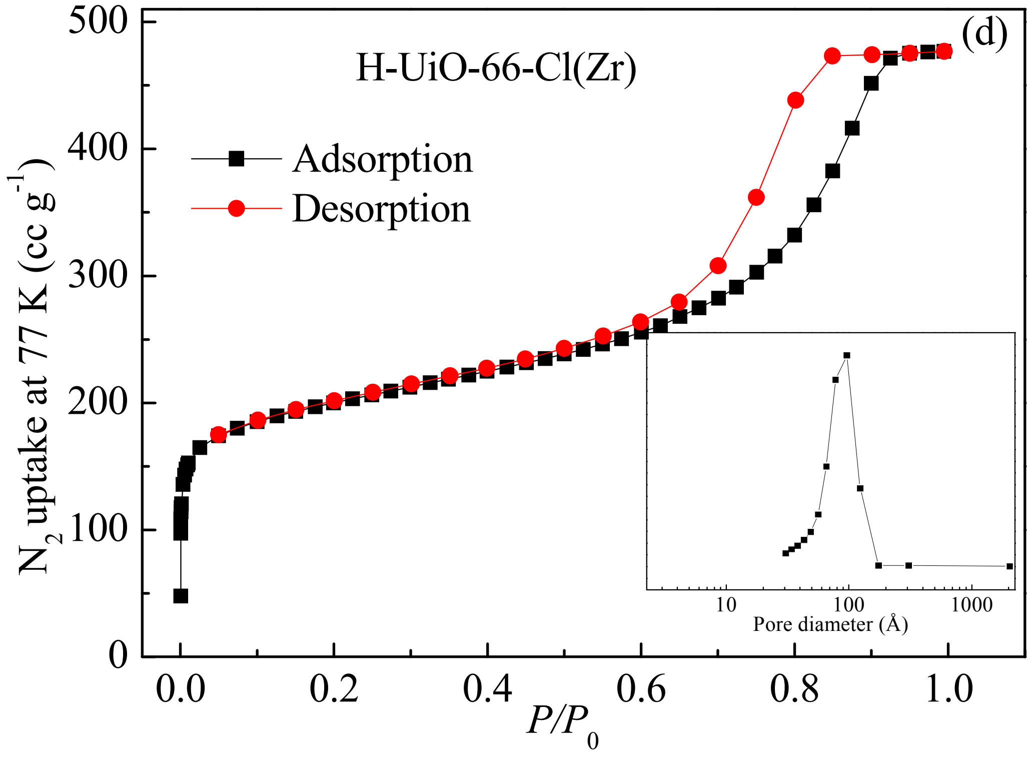
H-UiO-66-Cl(Zr)
(d)
Adsorption
Desorption
N2 uptake at 77 K (cc g-1)
P/P0
Pore diameter (Å)

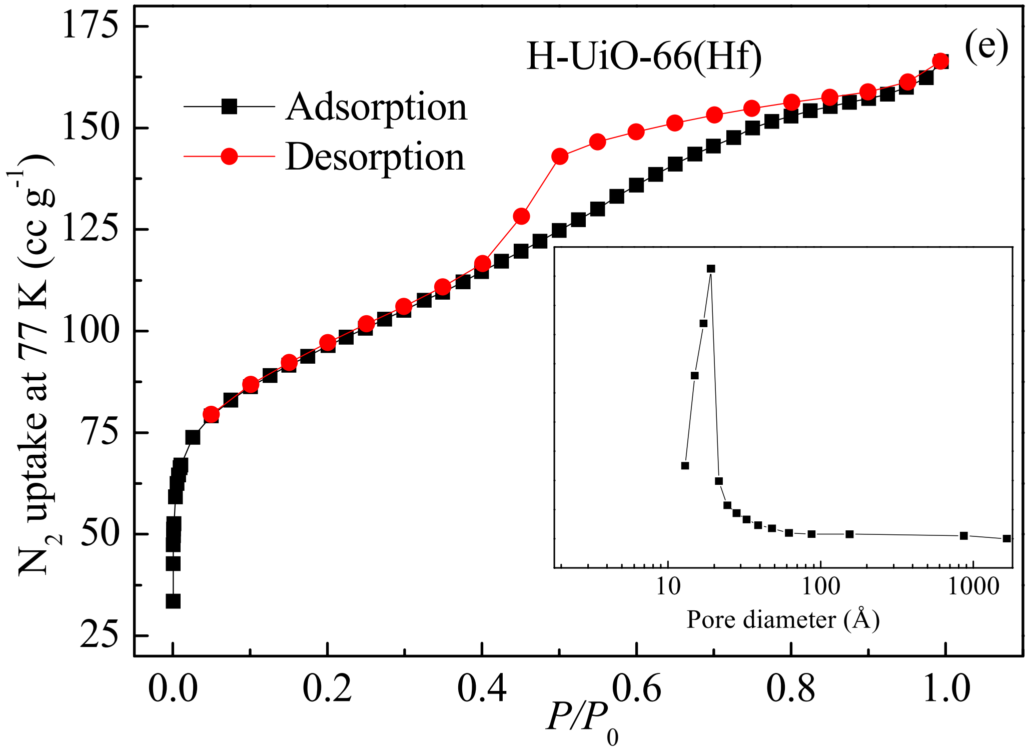
H-UiO-66(Hf)
(e)
Adsorption
Desorption
N2 uptake at 77 K (cc g-1)
P/P0
Pore diameter (Å)

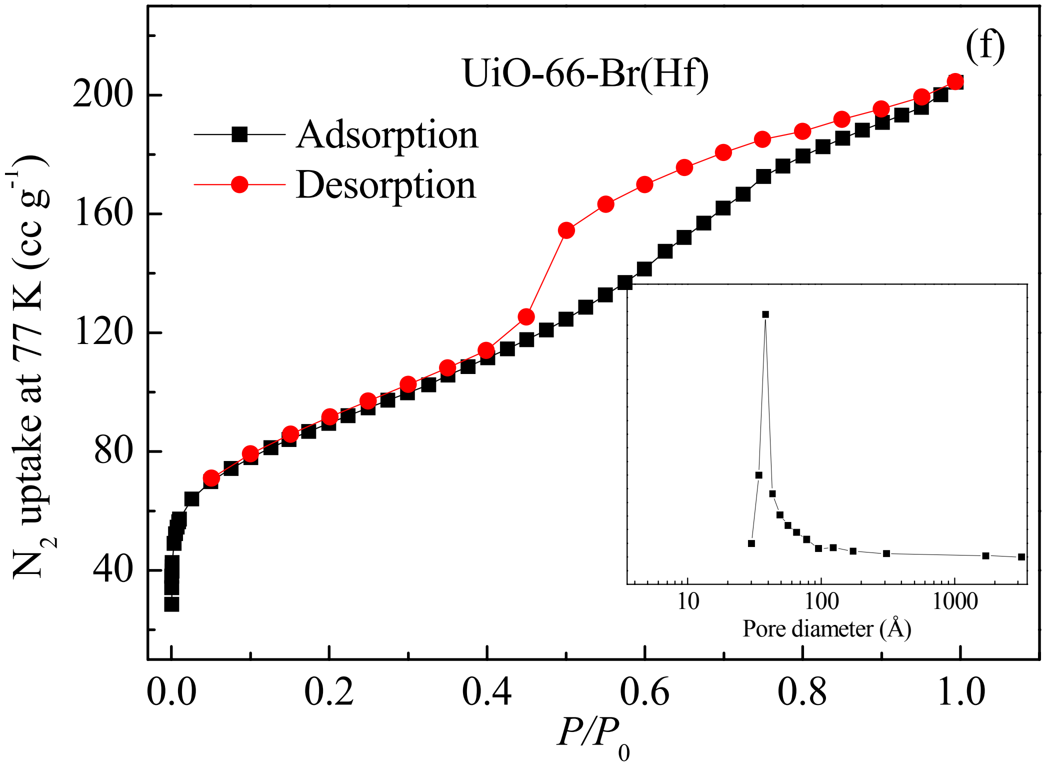

UiO-66-Br(Hf)
(f)
Adsorption
Desorption
N2 uptake at 77 K (cc g-1)
P/P0
Pore diameter (Å)

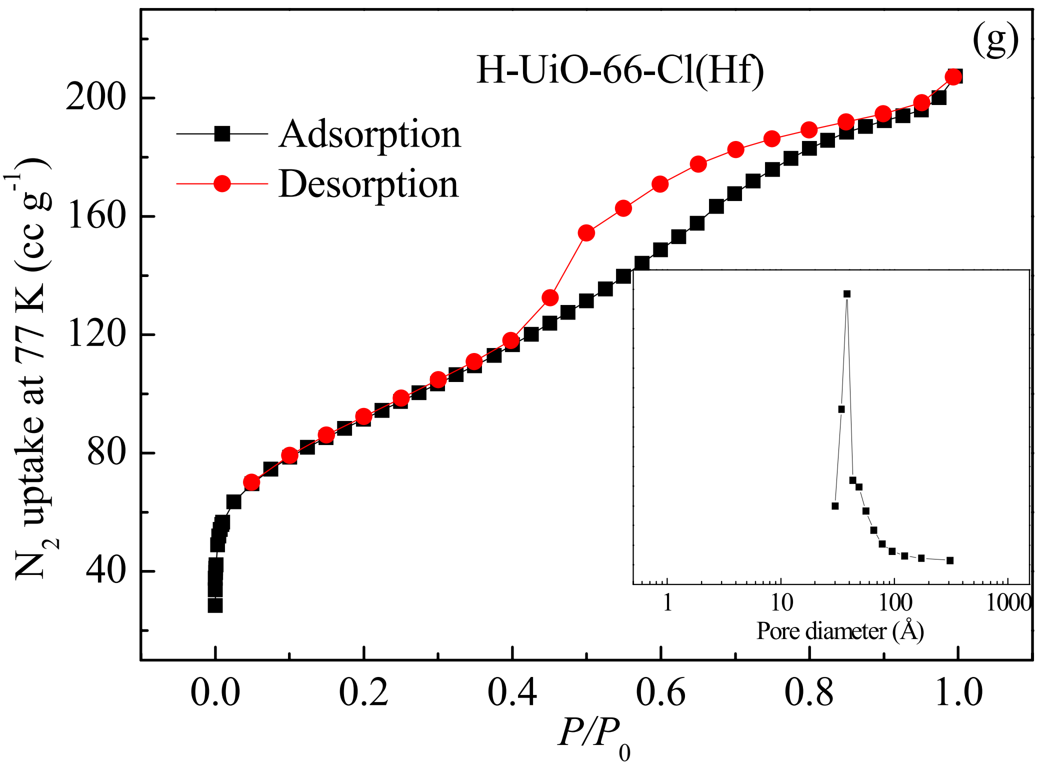

H-UiO-66-Cl(Hf)
(g)
Adsorption
Desorption
N2 uptake at 77 K (cc g-1)
P/P0
Pore diameter (Å)

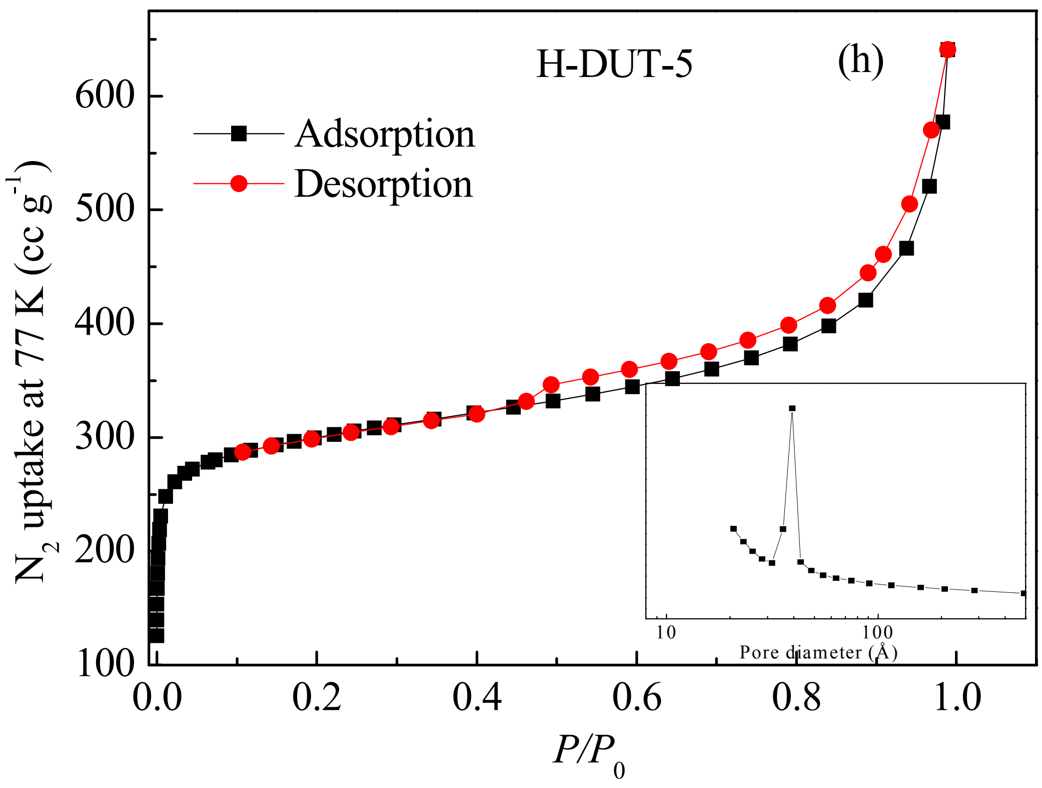

H-DUT-5
(h)
Adsorption
Desorption
N_2 uptake at 77 K (cc g^{-1})
100
200
300
400
500
600
0.0
0.2
0.4
0.6
0.8
1.0
P/P_0
10
100
Pore diameter (Å)

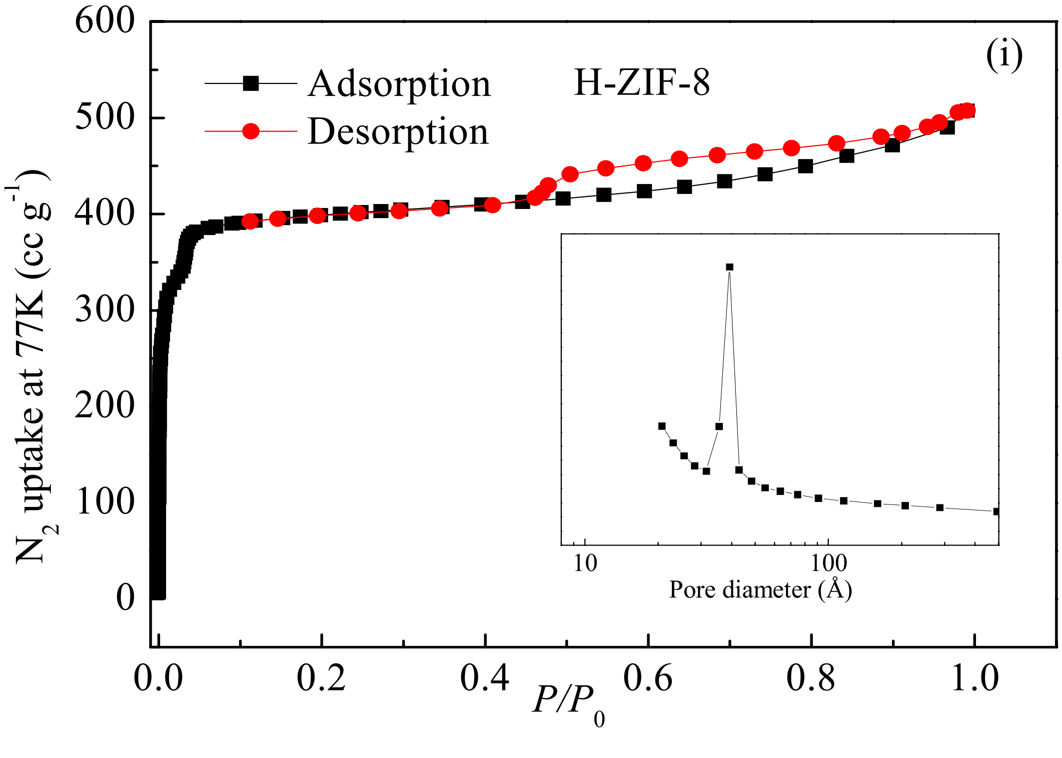

Adsorption
Desorption
H-ZIF-8
(i)
N_2 uptake at 77K (cc g^{-1})
0
100
200
300
400
500
600
0.0
0.2
0.4
0.6
0.8
1.0
P/P_0
10
100
Pore diameter (Å)

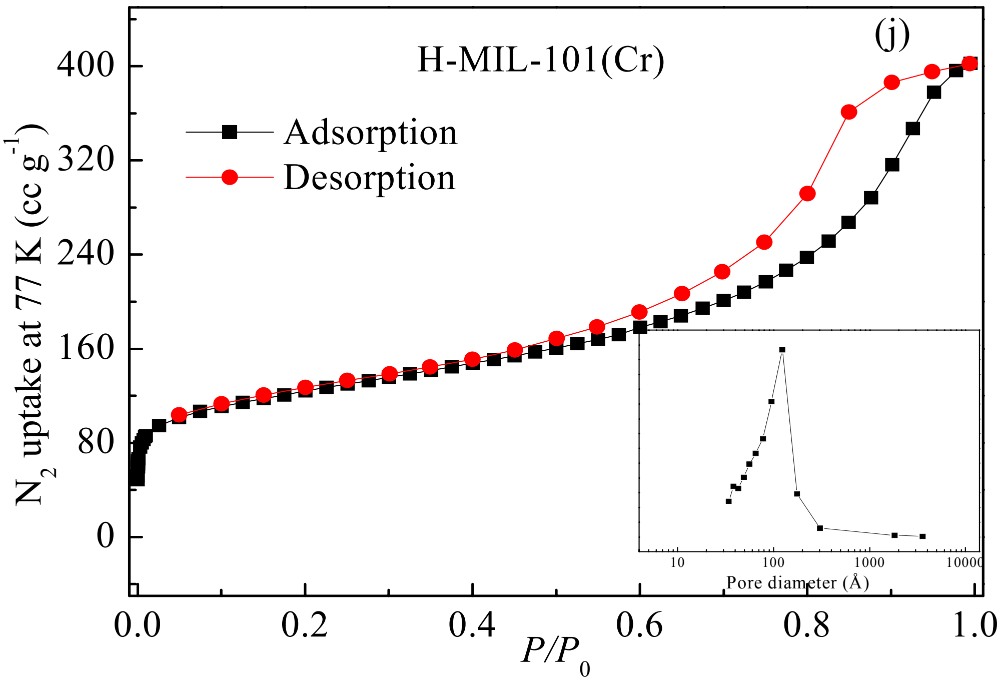

Supplementary Figure 12. N_2 adsorption-desorption isotherms at 77 K and pore size distributions of H-MOFs synthesized with different MOF precursor templates by one-pot reaction method. (**a**, **e**, **i**), MOF-5 precursors, (**b**) IRMOF-3 precursors, (**c**) MOF-5-NO_2 precursors, (**d**, **g**) MOF-5-Cl precursors, (**f**) MOF-5-Br precursors, (**h**) In-BPDC precursors, and (**j**) ZIF-8 precursors.

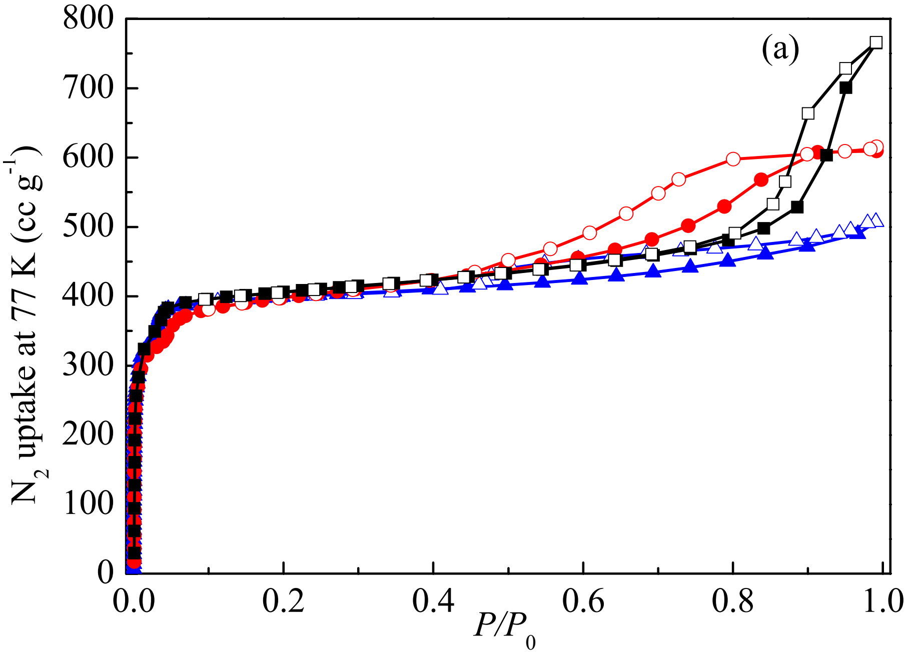

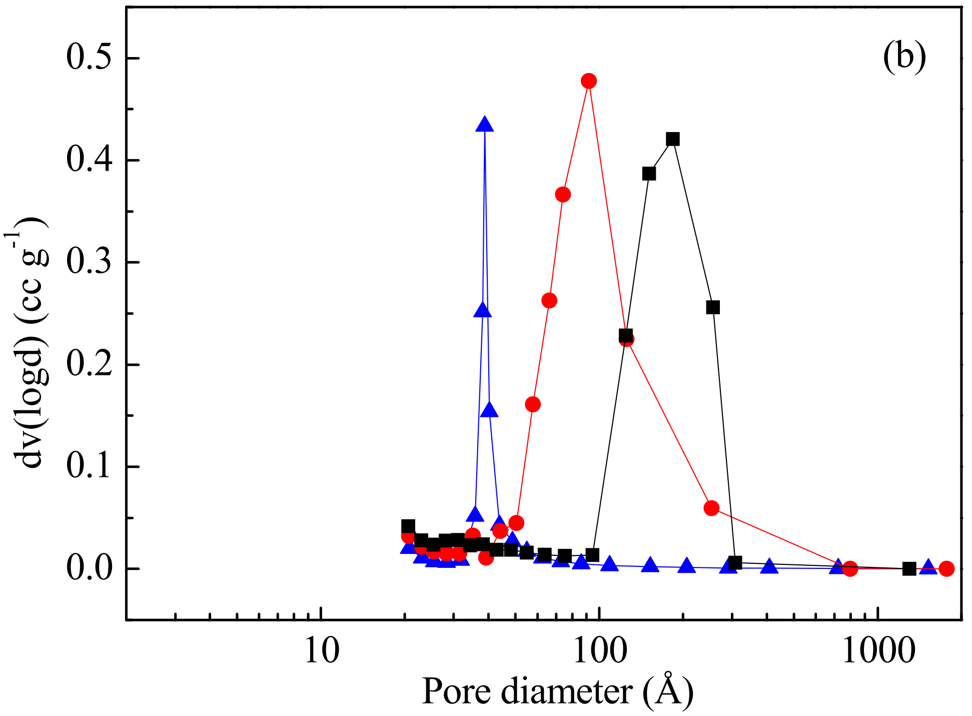

Supplementary Figure 13. N_2 adsorption-desorption isotherms at 77 K (**a**) and pore size distributions (**b**) of H-ZIF-8 prepared with different amounts of template. [preparation conditions: 0.1 g (black curve), 0.15 g (red curve), and 0.2 g (blue curve) of H_2BDC as ligand of MOF-5 template precursor].

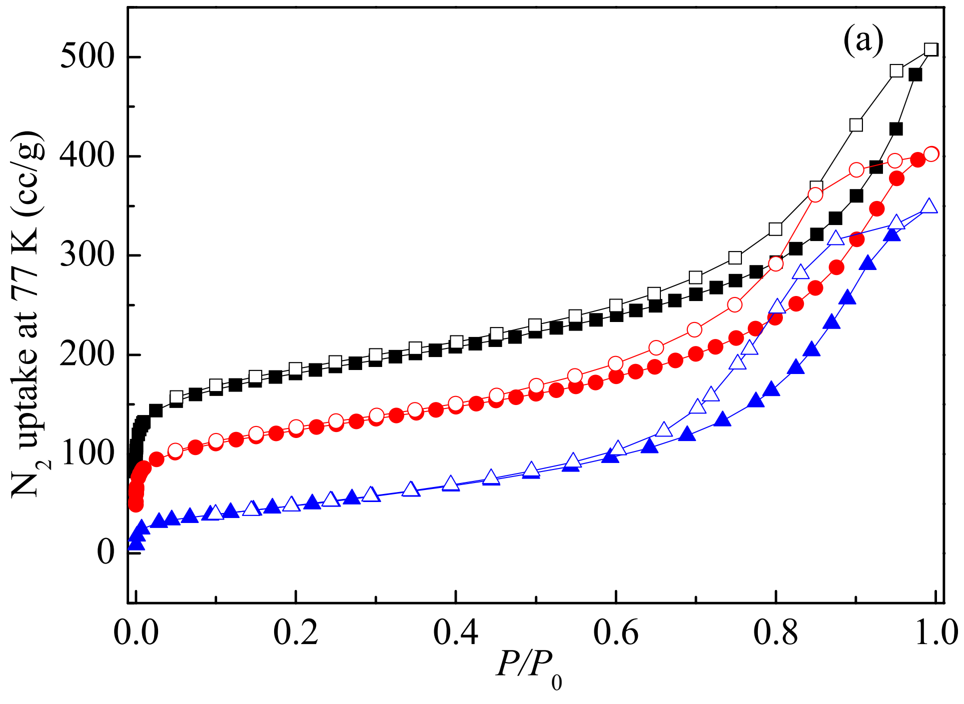

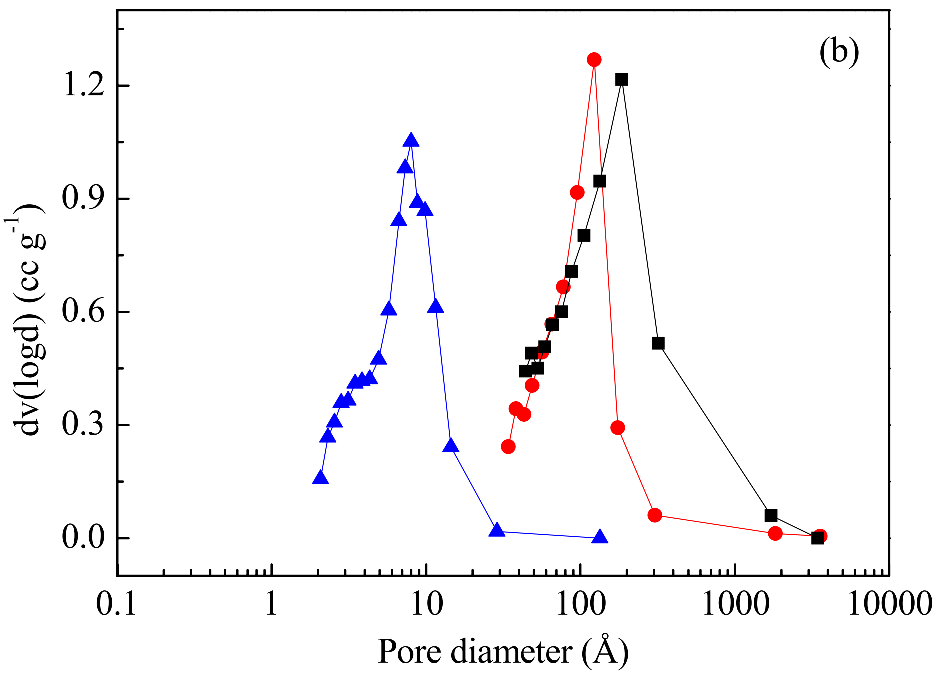

Supplementary Figure 14. N_2 adsorption-desorption isotherms at 77 K (**a**) and pore size distributions (**b**) of H-MIL-101(Cr) prepared with different amounts of template. [preparation conditions: 1.22 eqv (black curve), 1.83 eqv (red curve), and 2.44 eqv (blue curve) of ZIF-8 template precursor (eqv means the equivalent of ZIF-8 with respect to $Cr(NO_3)_3{\cdot}9H_2O$)].

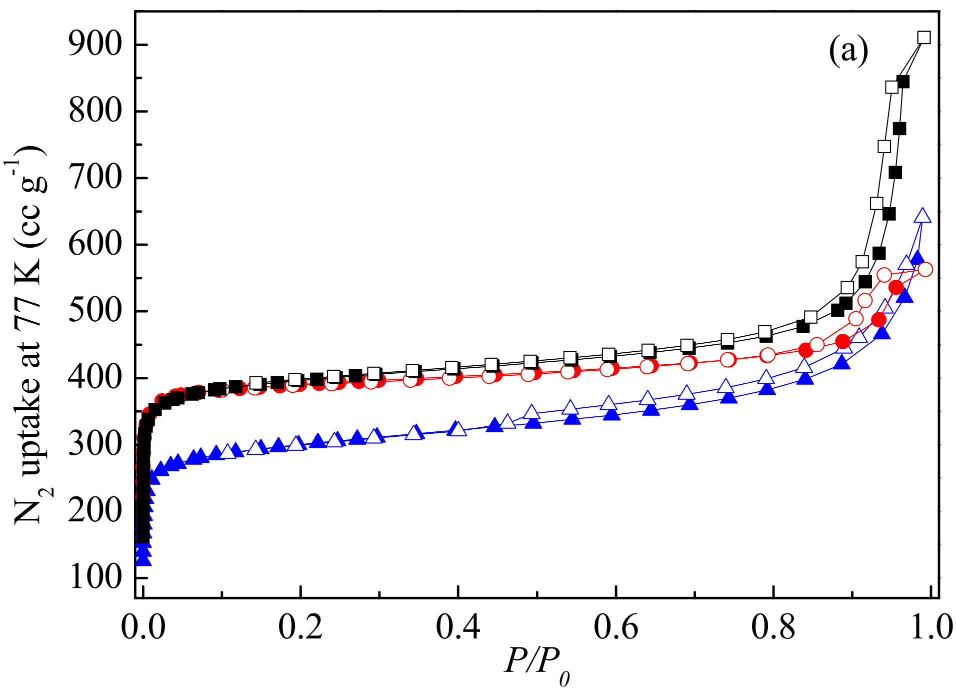

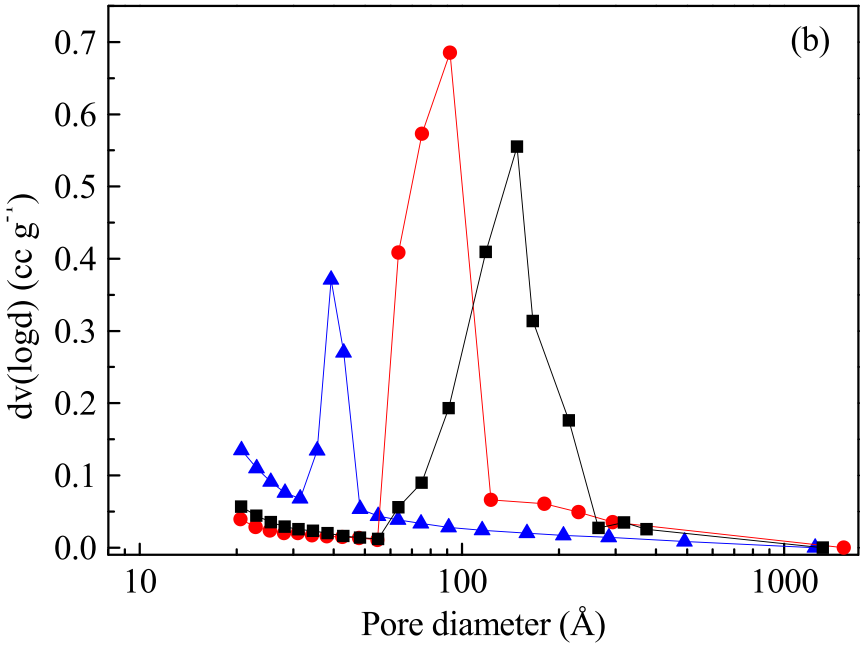

Supplementary Figure 15. N_2 adsorption-desorption isotherms at 77 K (**a**) and pore size distributions (**b**) of H-DUT-5 prepared with different amounts of template. [preparation conditions: 0.4 g (black curve), 0.5 g (red curve), and 0.7 g (blue curve) of $In(NO_3)_2{\cdot}xH_2O$ as metal source of In-BPDC template precursor].

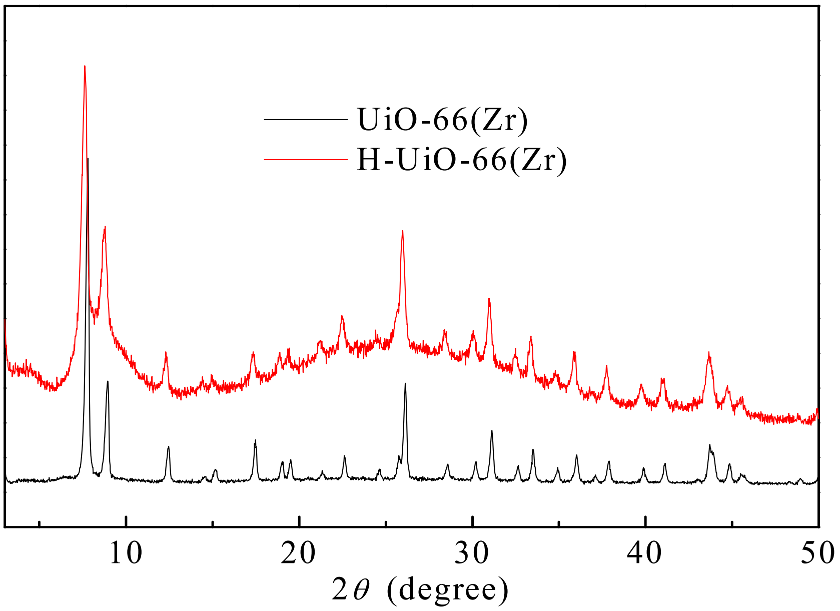

Supplementary Figure 16. PXRD pattern of H-UiO-66(Zr) synthesized using $Zn_4O(BC)_6$ as template precursors by a two-step process, as well as that of the UiO-66 parent.

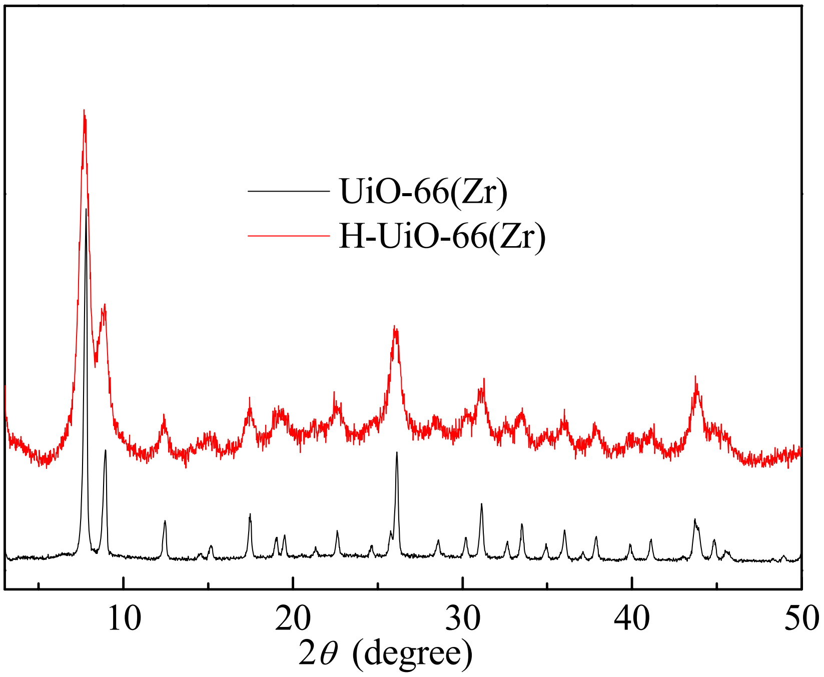

Supplementary Figure 17. PXRD pattern of H-UiO-66(Zr) synthesized using MOP-*t*Bu as template precursors by a two-step process, as well as that of the UiO-66 parent.

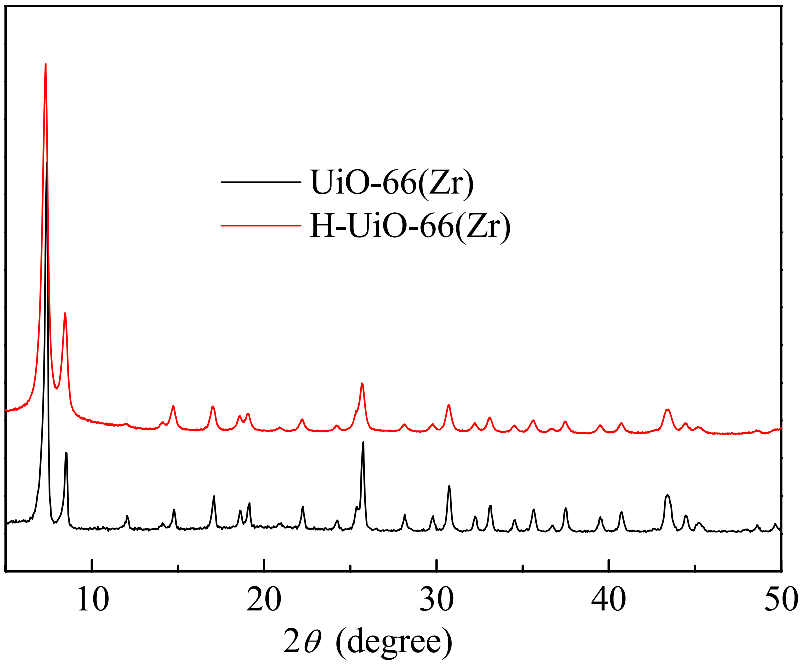

Supplementary Figure 18. PXRD pattern of H-UiO-66(Zr) synthesized using MOF-5 as template precursors by a two-step process, as well as that of the UiO-66 parent.

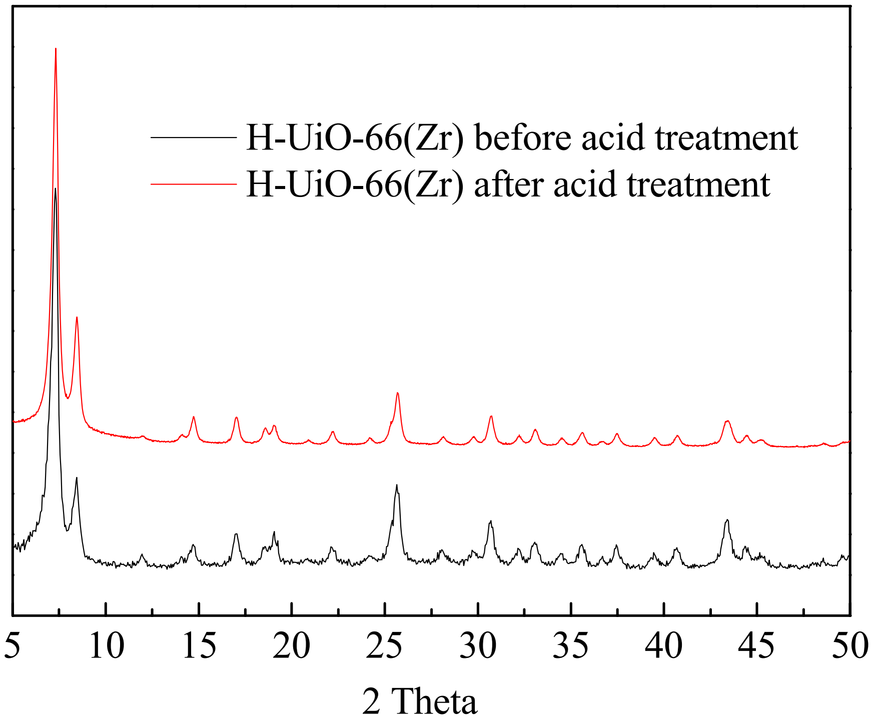

Supplementary Figure 19. PXRD patterns of H-UiO-66(Zr) prepared with MOF-5 as template precursors by a two-step process before and after acid treatment.

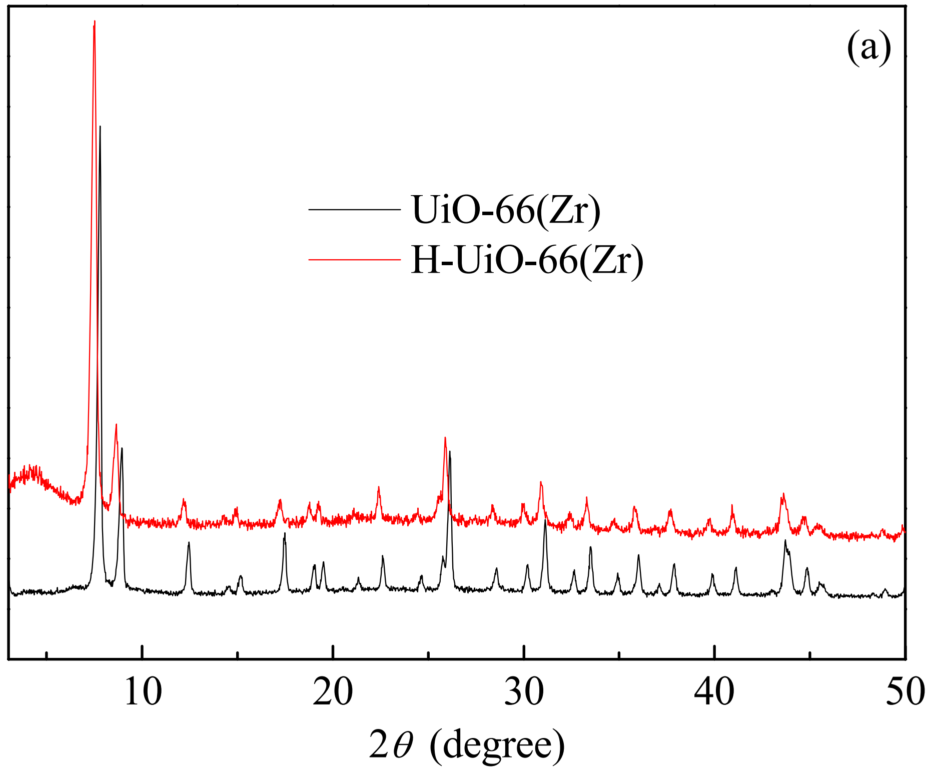

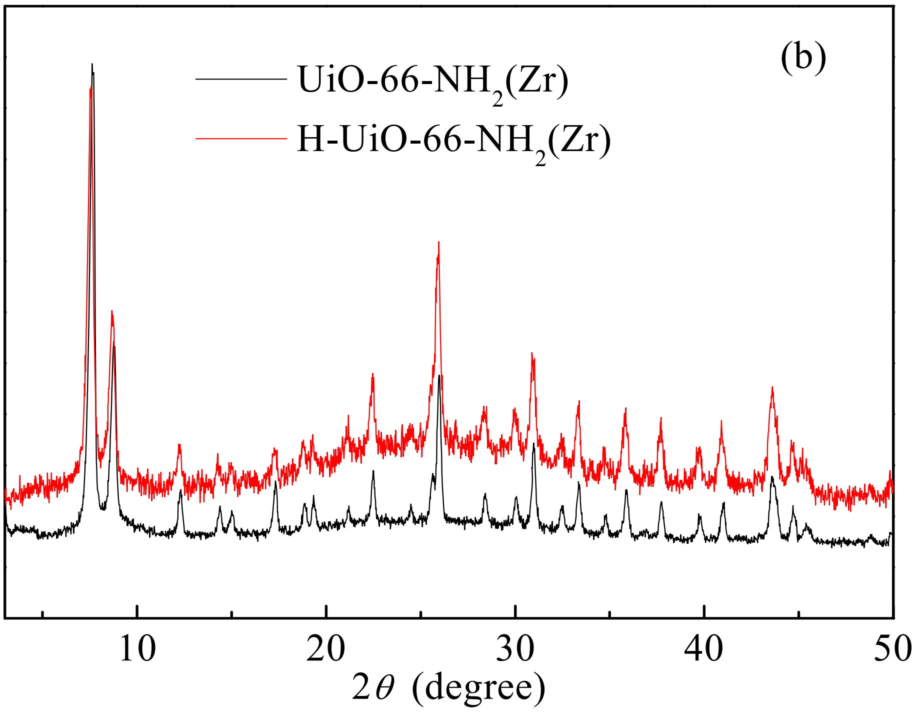

UiO-66-NH2(Zr)
H-UiO-66-NH2(Zr)
(b)
10
20
30
40
50
2θ (degree)

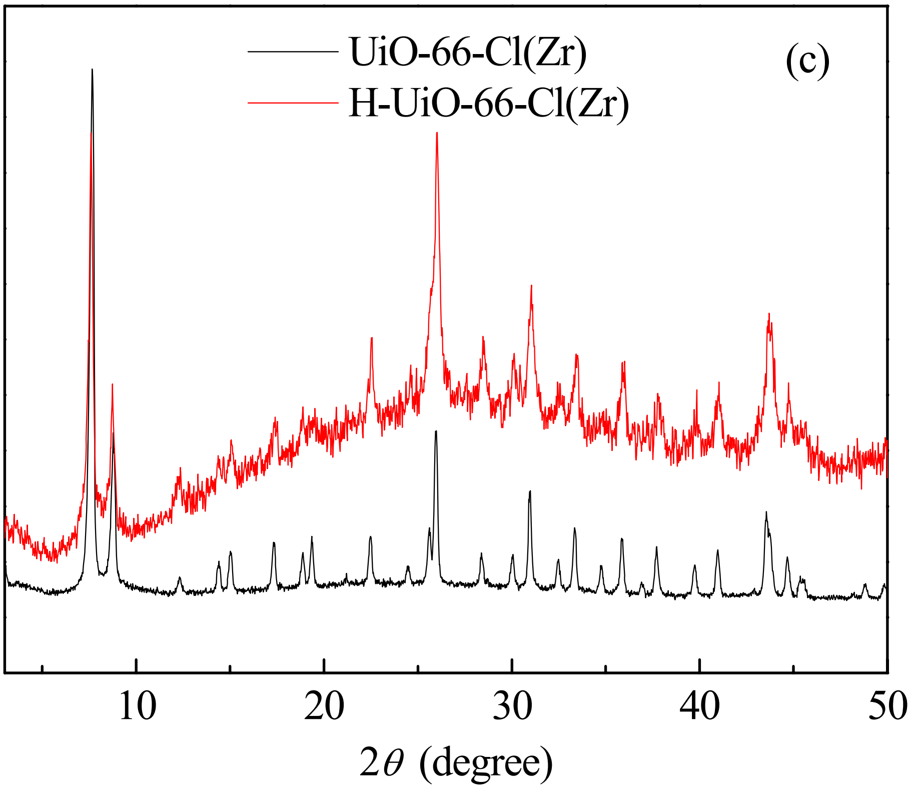

UiO-66-Cl(Zr)
H-UiO-66-Cl(Zr)
(c)
10
20
30
40
50
2θ (degree)

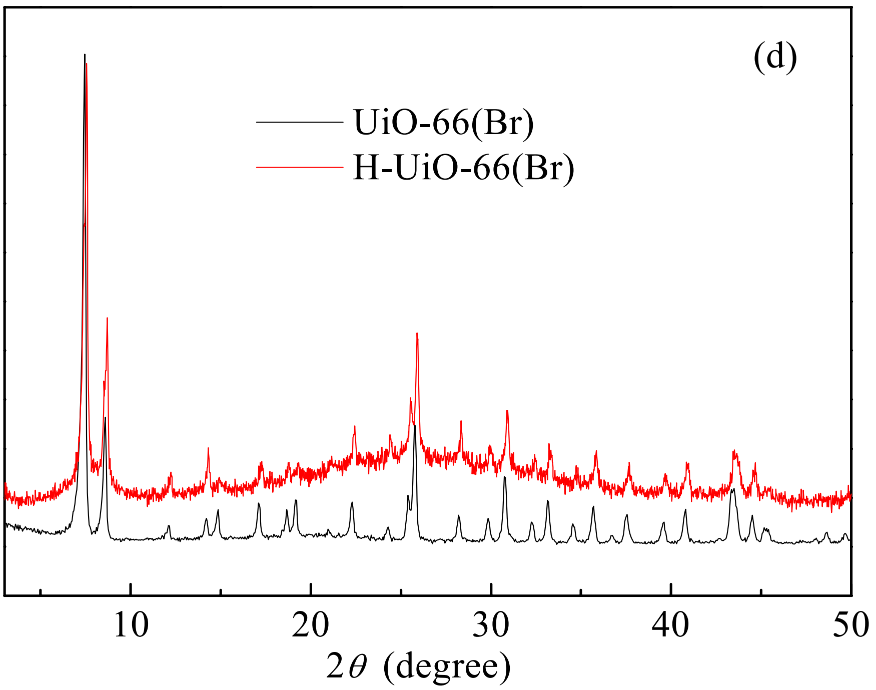

Supplementary Figure 20. PXRD patterns of H-MOFs synthesized using $Zn_4O(BC)_6$ precursors as templates by one-pot reaction method, as well as those of related UiO-66 parents.

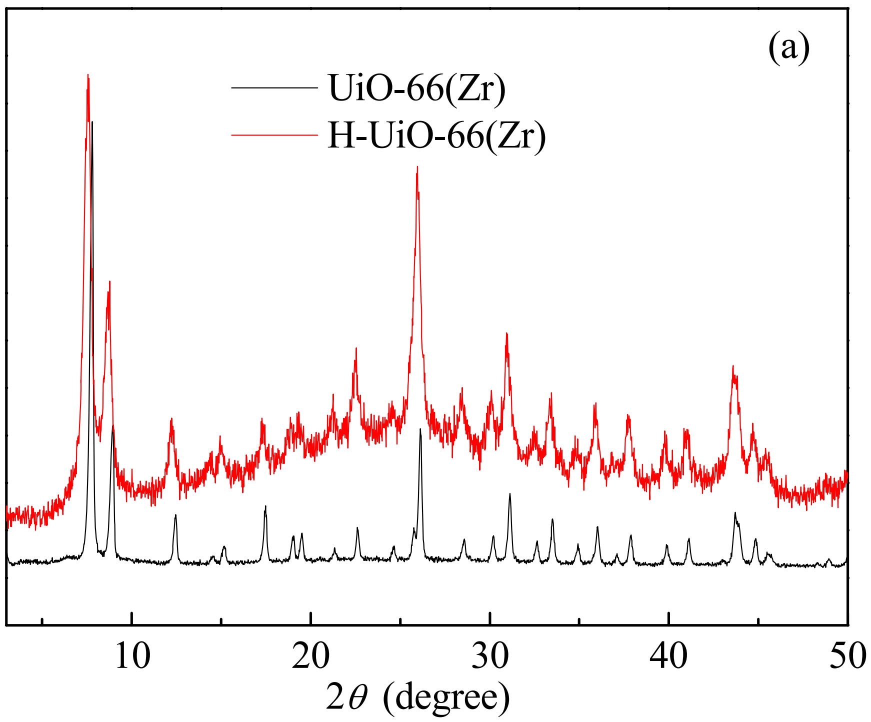

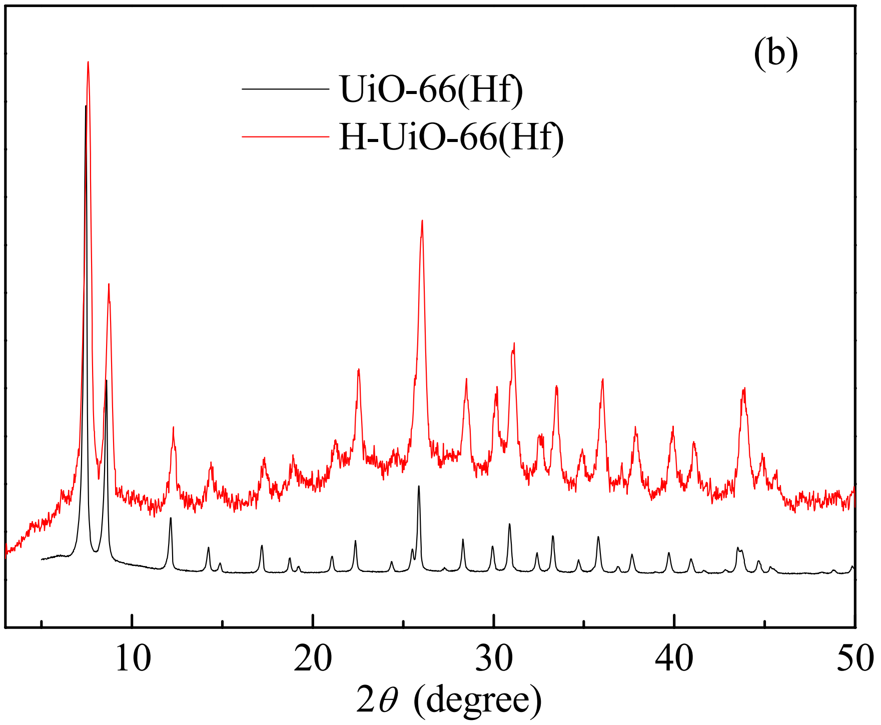

(b)
UiO-66(Hf)
H-UiO-66(Hf)
10
20
30
40
50
2θ (degree)

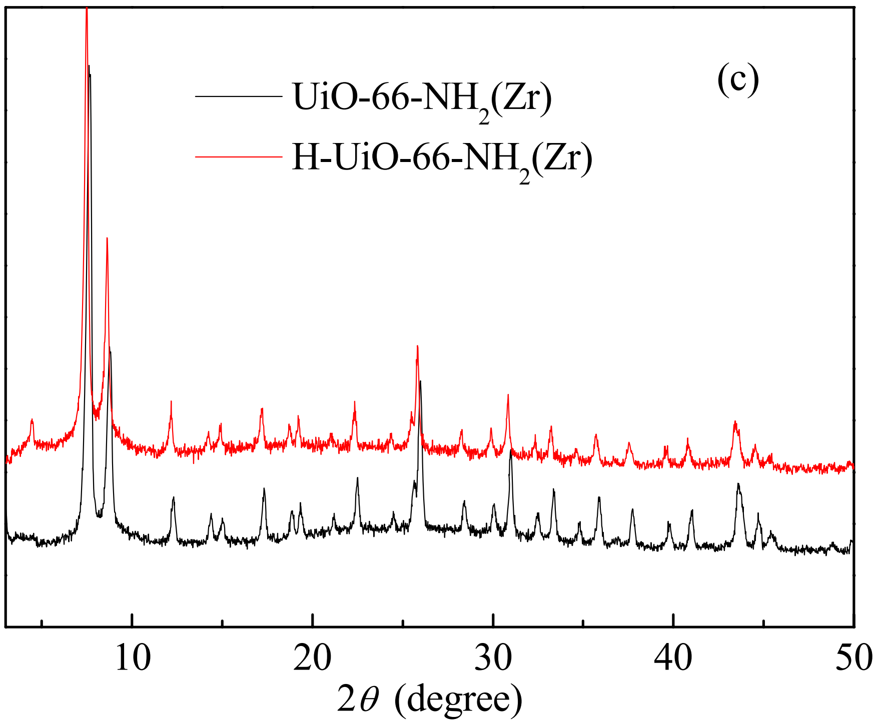

(c)
UiO-66-NH2(Zr)
H-UiO-66-NH2(Zr)
10
20
30
40
50
2θ (degree)

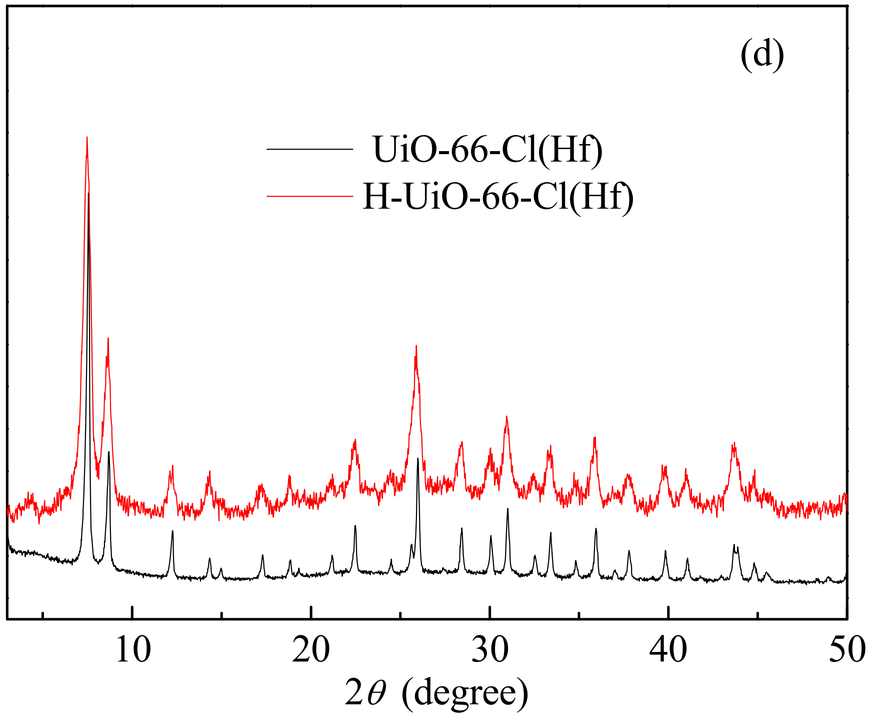

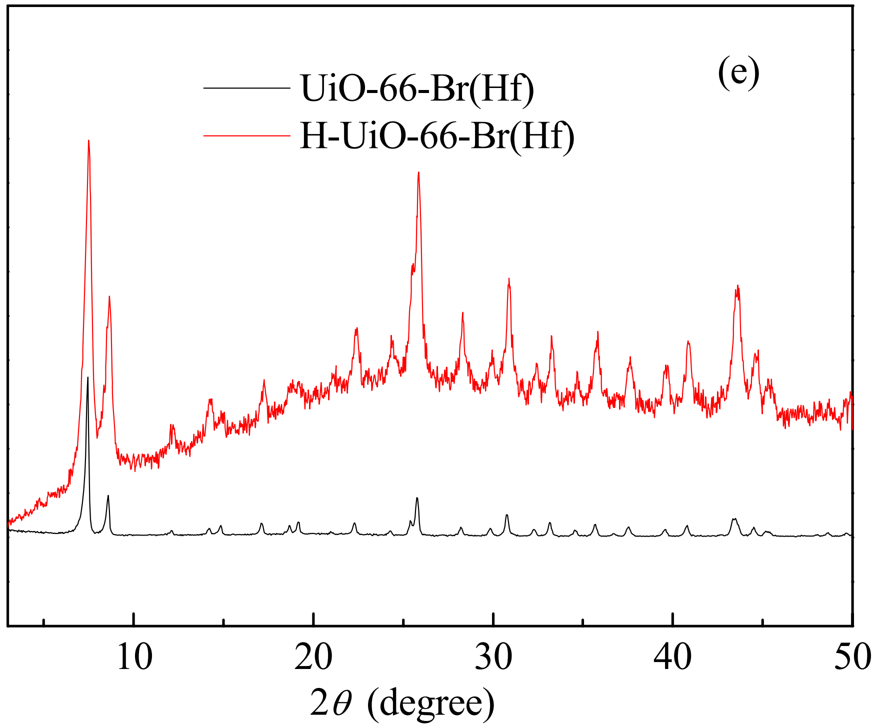

Supplementary Figure 21. PXRD patterns of H-MOFs synthesized using MOP-*t*Bu precursors templates by one-pot reaction method, as well as those of related UiO-66 parents.

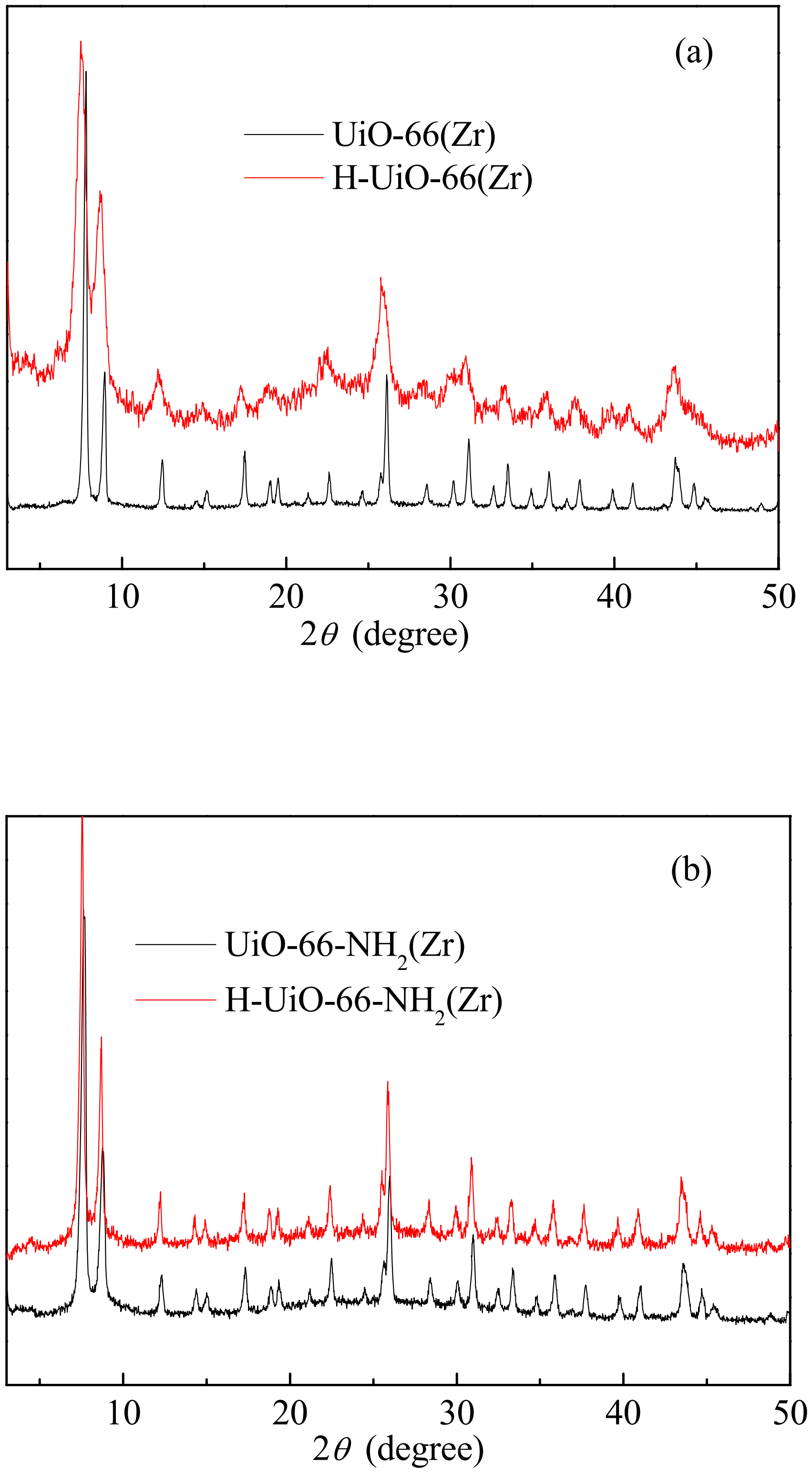
(a)
UiO-66(Zr)
H-UiO-66(Zr)
10
20
30
40
50
2θ (degree)
(b)
UiO-66-NH2(Zr)
H-UiO-66-NH2(Zr)
10
20
30
40
50
2θ (degree)

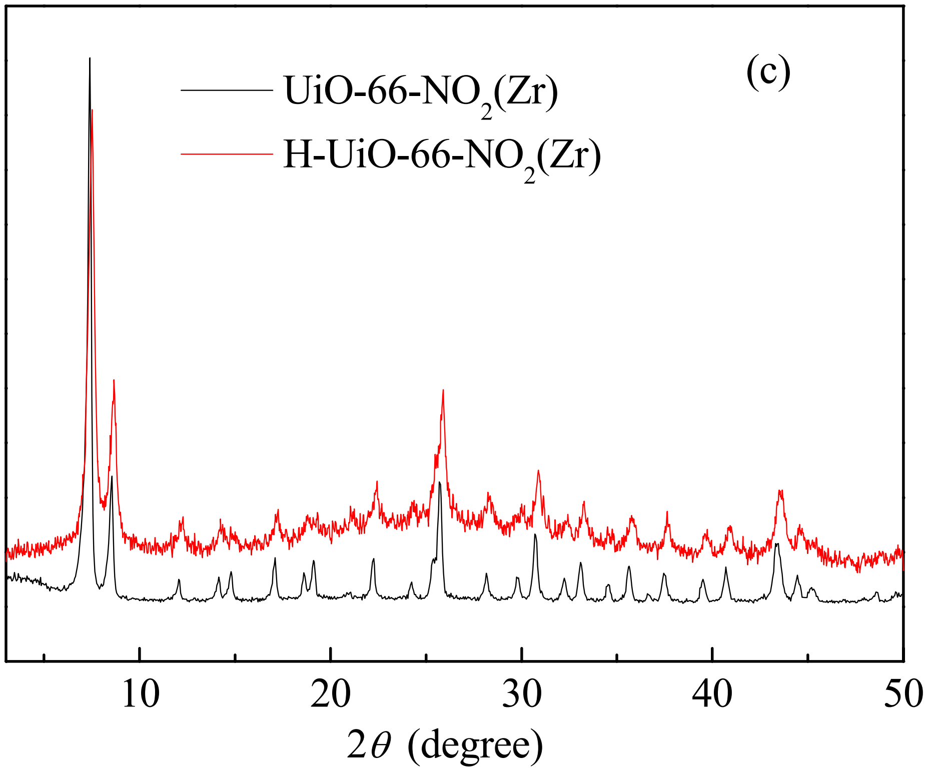
UiO-66-NO2(Zr)
H-UiO-66-NO2(Zr)
(c)
10
20
30
40
50
2θ (degree)

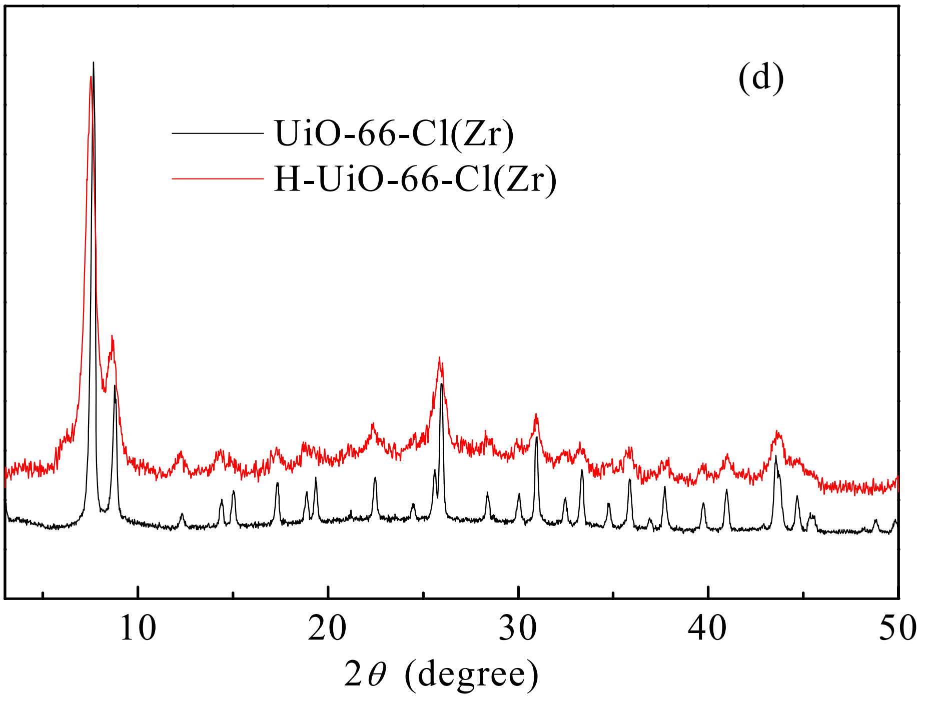
(d)
UiO-66-Cl(Zr)
H-UiO-66-Cl(Zr)
10
20
30
40
50
2θ (degree)

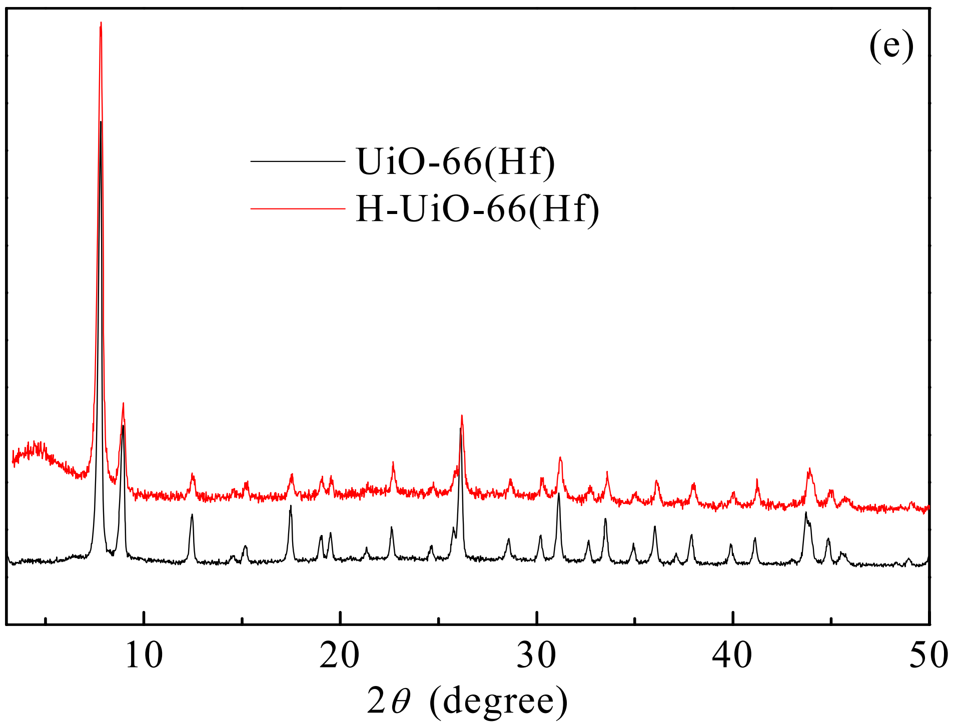
(e)
UiO-66(Hf)
H-UiO-66(Hf)
10
20
30
40
50
2θ (degree)

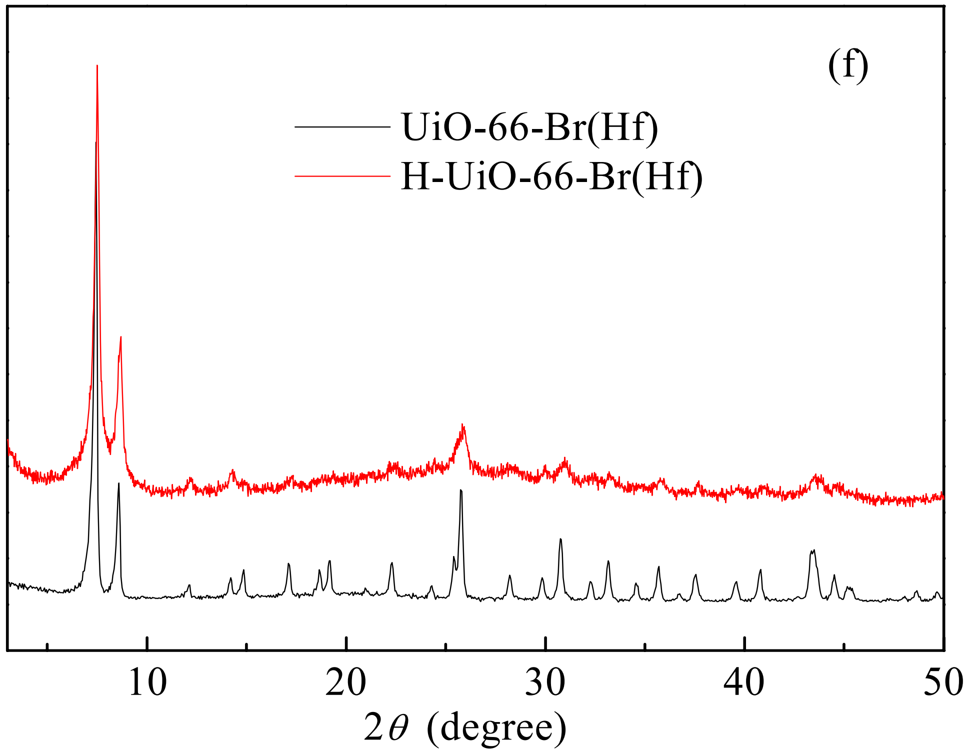
(f)
UiO-66-Br(Hf)
H-UiO-66-Br(Hf)
10
20
30
40
50
2θ (degree)

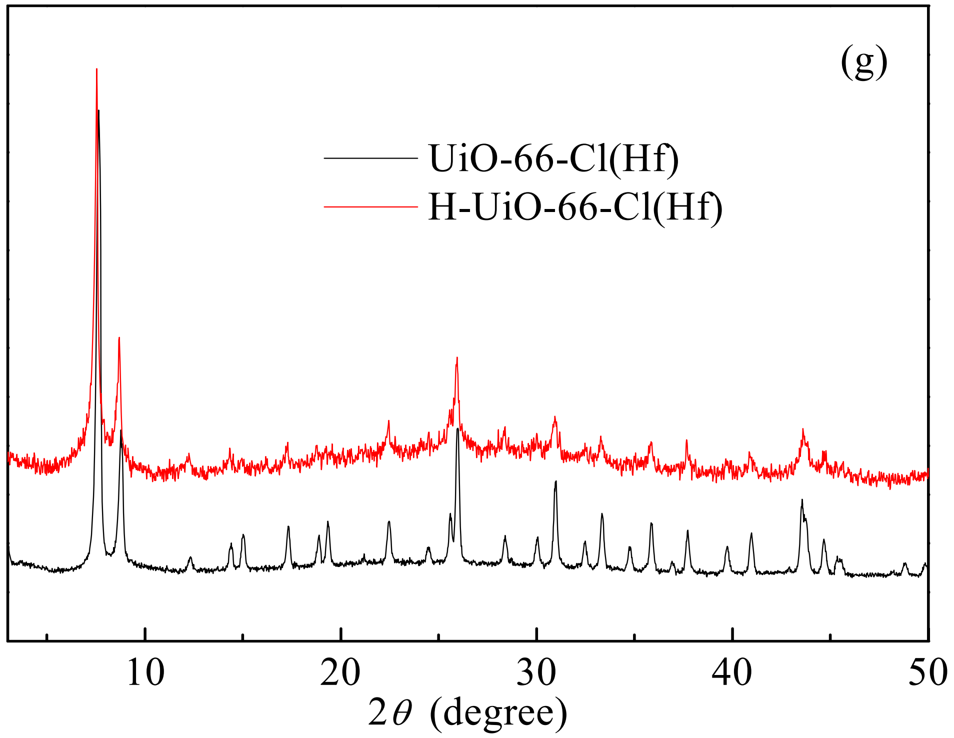
(g)
UiO-66-Cl(Hf)
H-UiO-66-Cl(Hf)
10
20
30
40
50
2θ (degree)

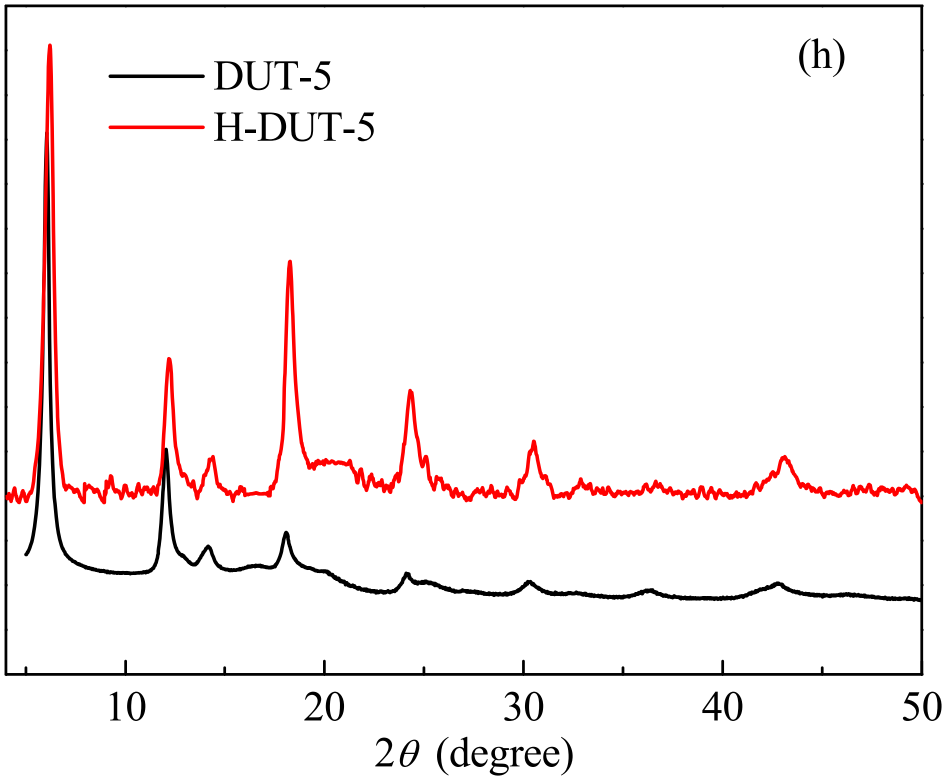
(h)
DUT-5
H-DUT-5
10
20
30
40
50
2θ (degree)

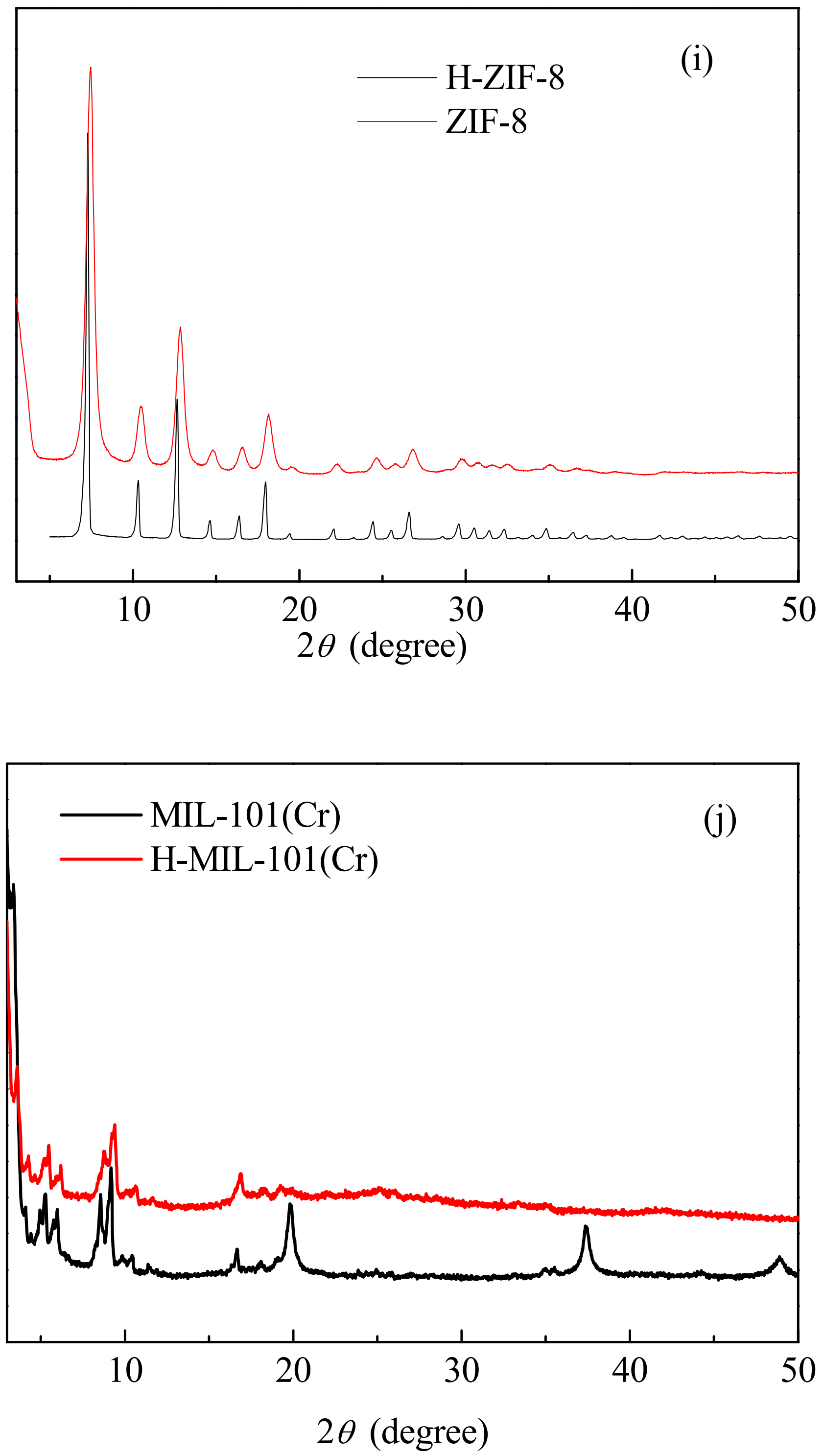

Supplementary Figure 22. PXRD patterns of H-MOFs synthesized using MOFs precursors templates by one-pot reaction method, as well as those of related MOFs parents. (**a**, **e**, **i**), MOF-5 precursors, (**b**) IRMOF-3 precursors, (**c**) MOF-5-NO_2 precursors, (**d**, **g**) MOF-5-Cl precursors, (**f**) MOF-5-Br precursors, (**h**) In-BPDC precursors, and (**j**) ZIF-8 precursors.

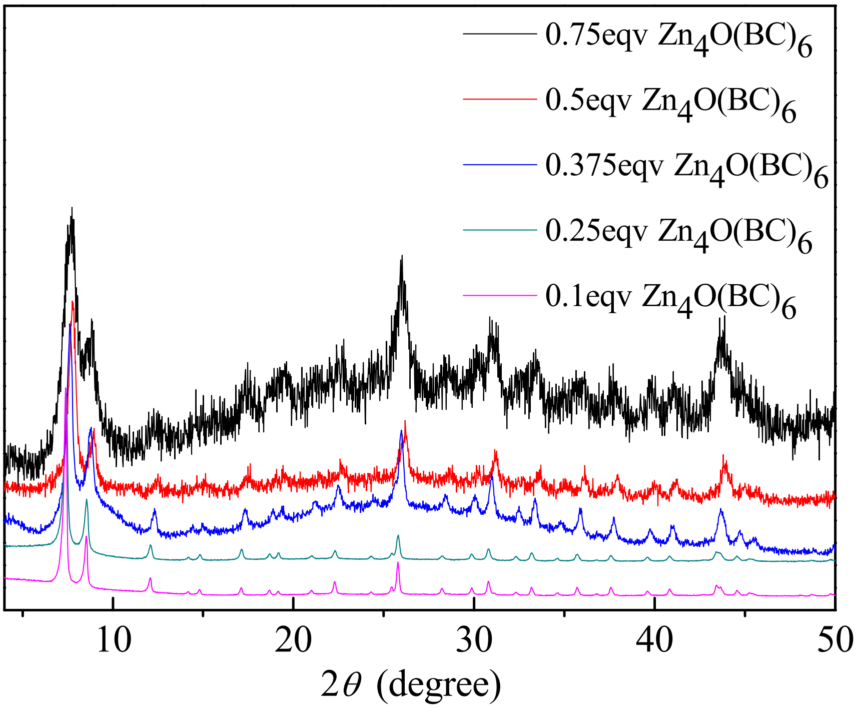

Supplementary Figure 23. PXRD patterns of UiO-66(Zr) samples prepared with different amounts of $Zn_4O(BC)_6$ template (eqv means the equivalent of $Zn_4O(BC)_6$ with respect to $ZrCl_4$).

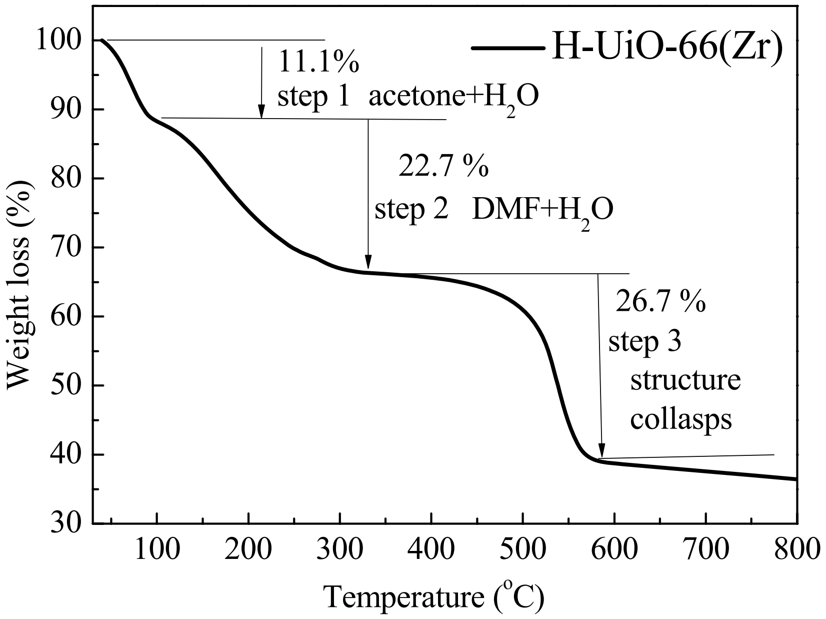

Supplementary Figure 24. TGA curve of H-UiO-66(Zr) synthesized using $Zn_4O(BC)_6$ as template precursors by a two-step process.

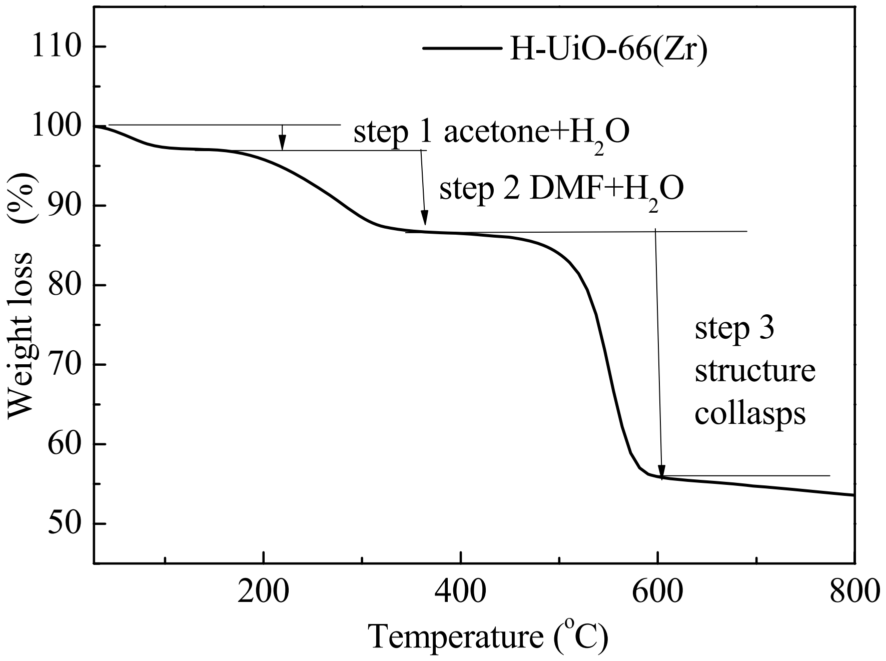

Supplementary Figure 25. TGA curve of H-UiO-66(Zr) synthesized using MOP-*t*Bu as template precursors by a two-step process.

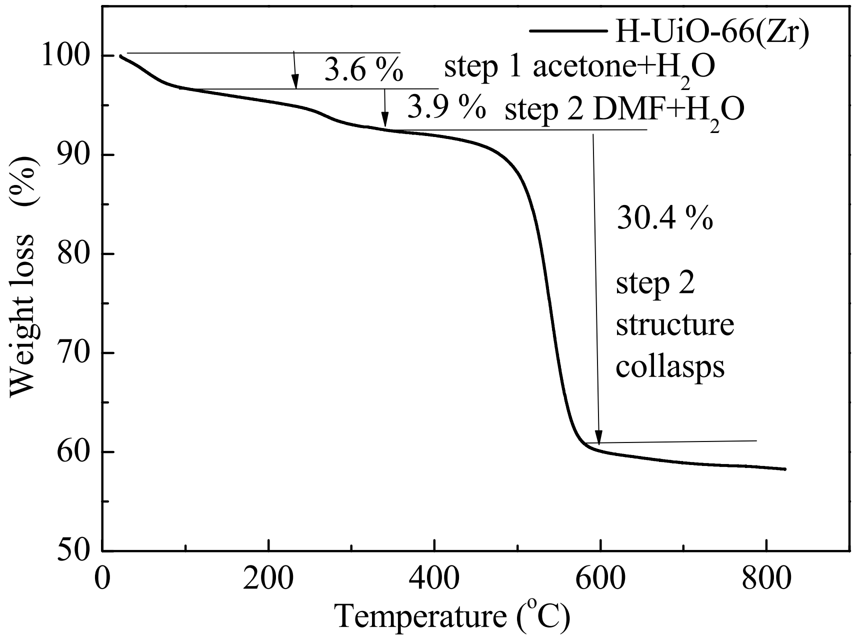

Supplementary Figure 26. TGA curve of H-UiO-66(Zr) synthesized using MOF-5 as template precursors by a two-step process.

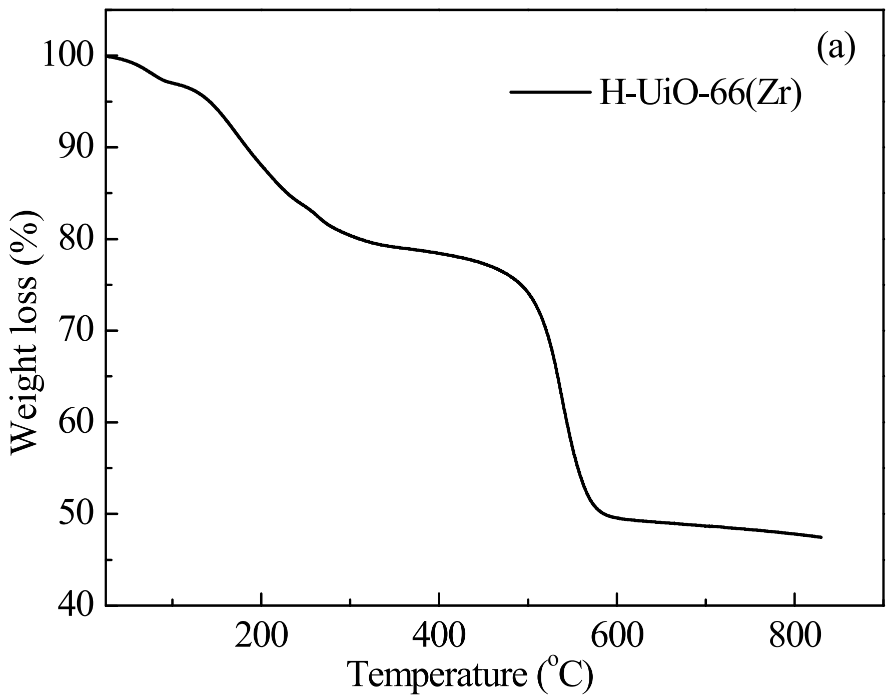
(a)
H-UiO-66(Zr)
Weight loss (%)
Temperature (°C)
100
90
80
70
60
50
40
200
400
600
800

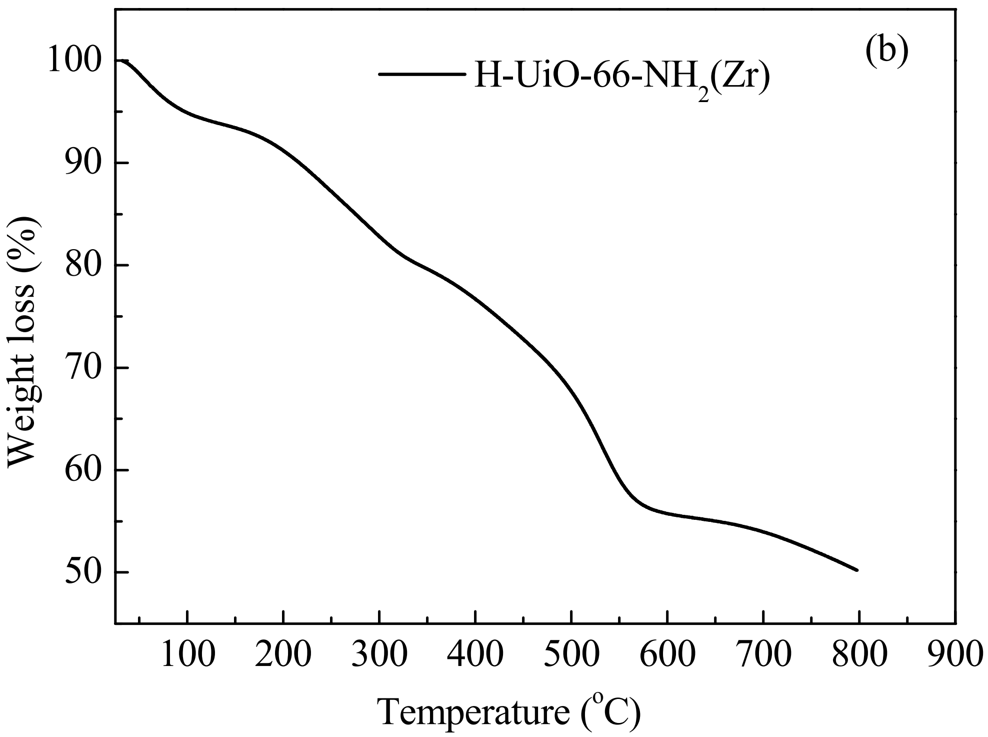
(b)
H-UiO-66-NH2(Zr)
Weight loss (%)
Temperature (°C)
100
90
80
70
60
50
100
200
300
400
500
600
700
800
900

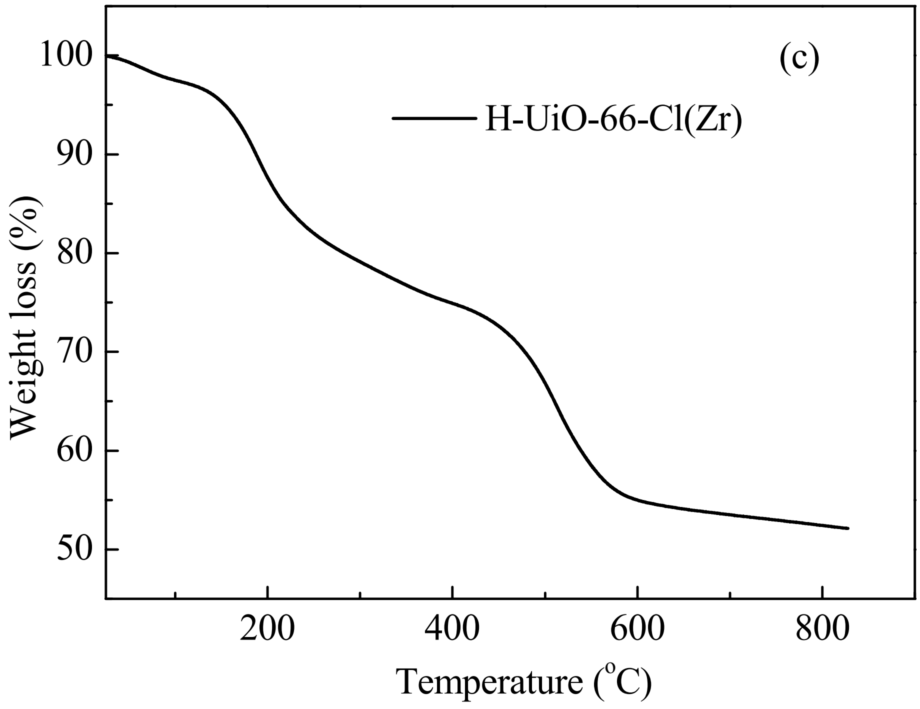

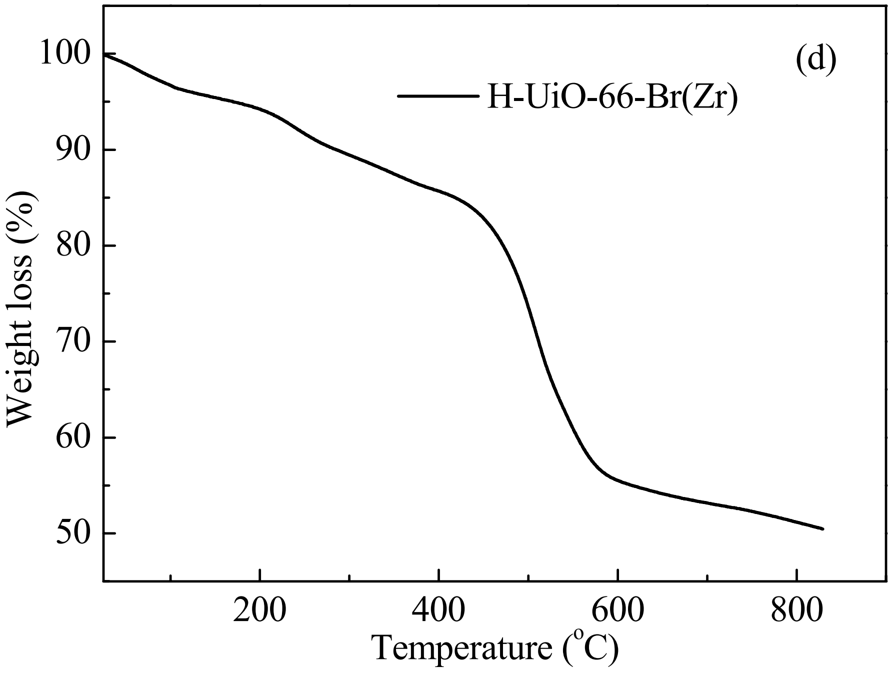

Supplementary Figure 27. TGA curves of H-UiO-66-X(Zr) synthesized using $Zn_4O(BC)_6$ precursors templates by one-pot reaction method.

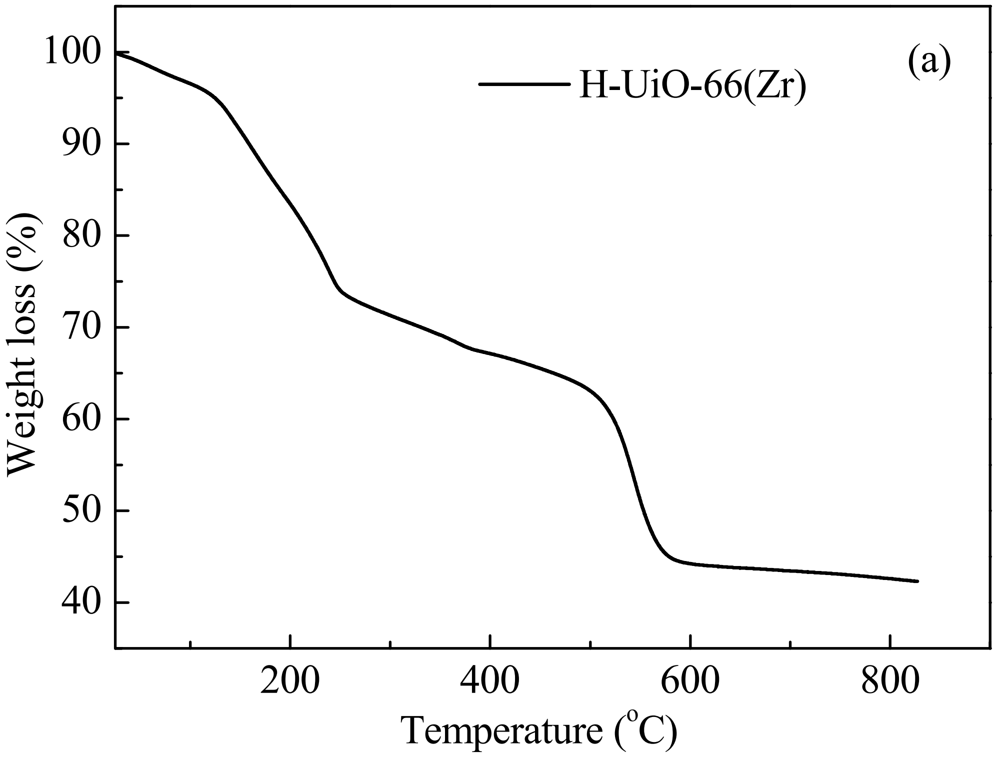

(a)
H-UiO-66(Zr)
Weight loss (%)
Temperature (°C)

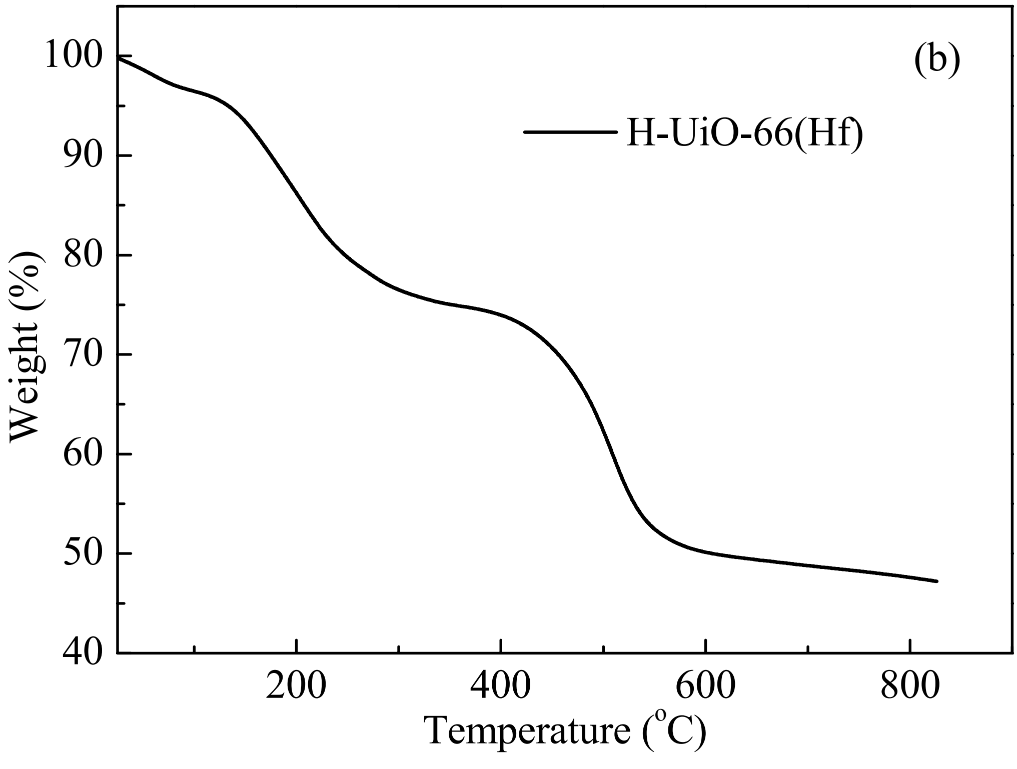

(b)
H-UiO-66(Hf)
Weight (%)
Temperature (°C)

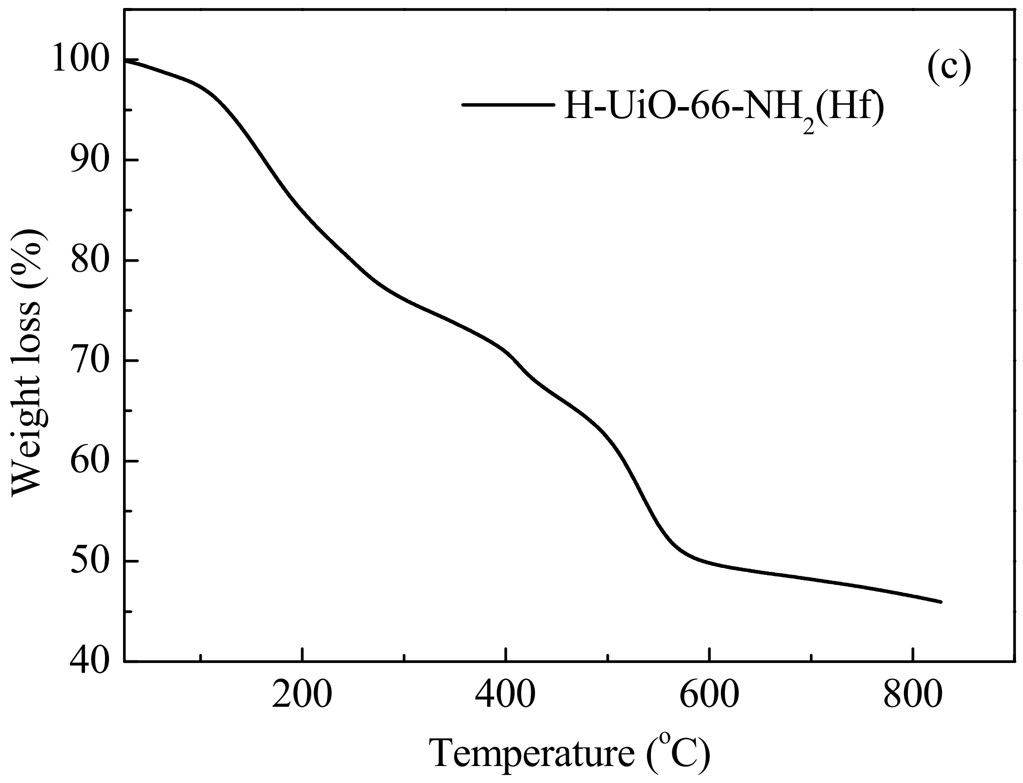
(c)
H-UiO-66-NH2(Hf)
Weight loss (%)
Temperature (°C)
100
90
80
70
60
50
40
200
400
600
800

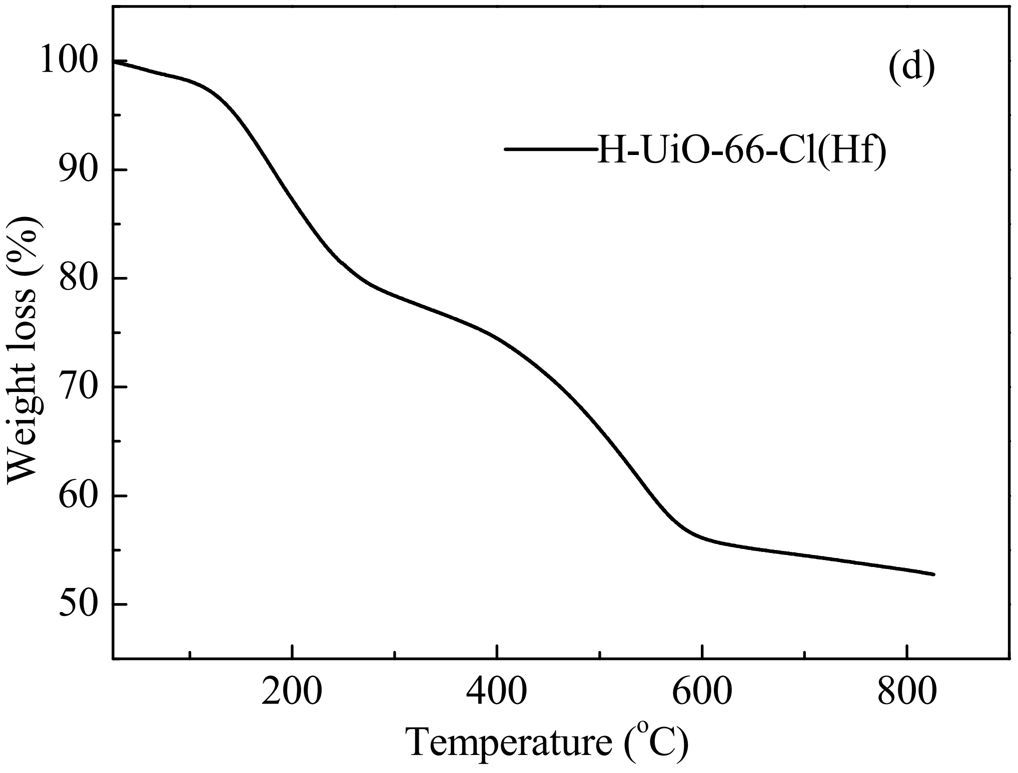
(d)
H-UiO-66-Cl(Hf)
Weight loss (%)
Temperature (°C)
100
90
80
70
60
50
200
400
600
800

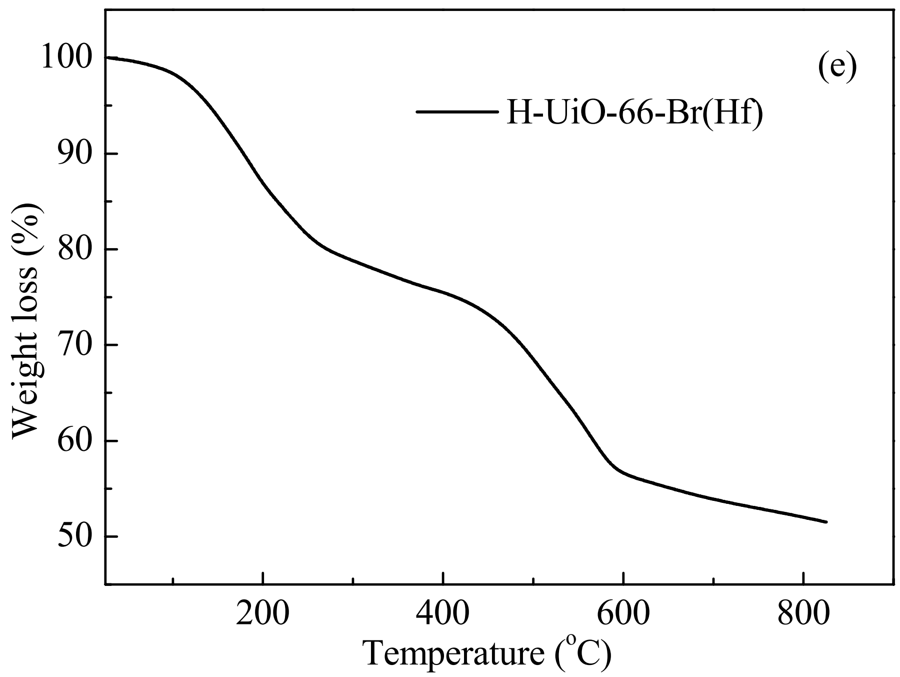

Supplementary Figure 28. TGA curves of H-UiO-66-X synthesized using MOP-*t*Bu precursors templates by one-pot reaction method.

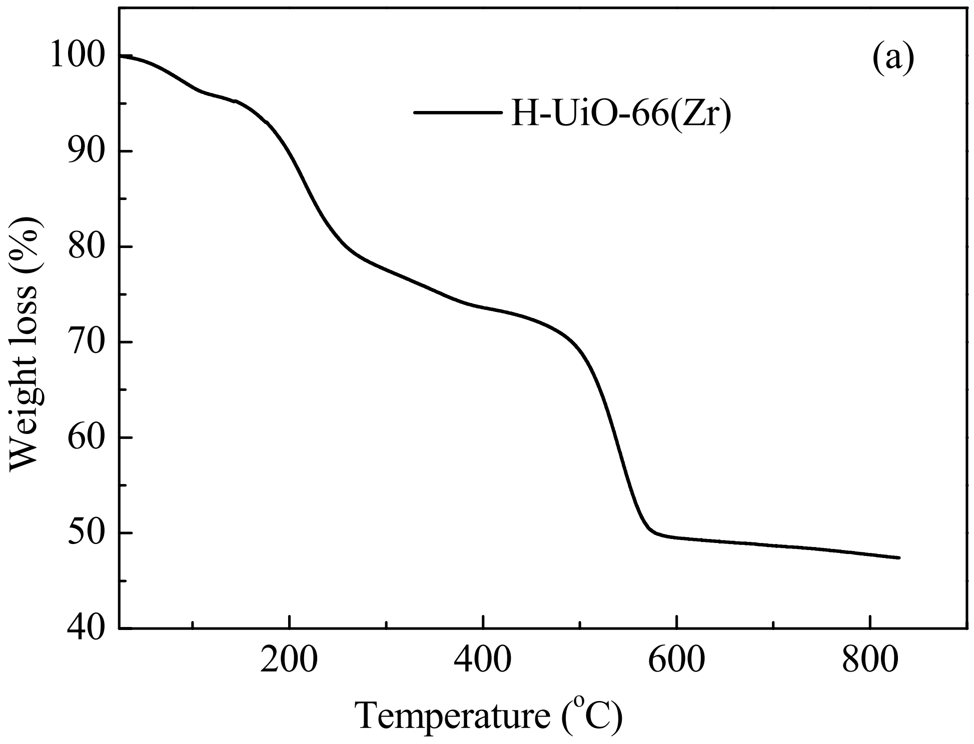

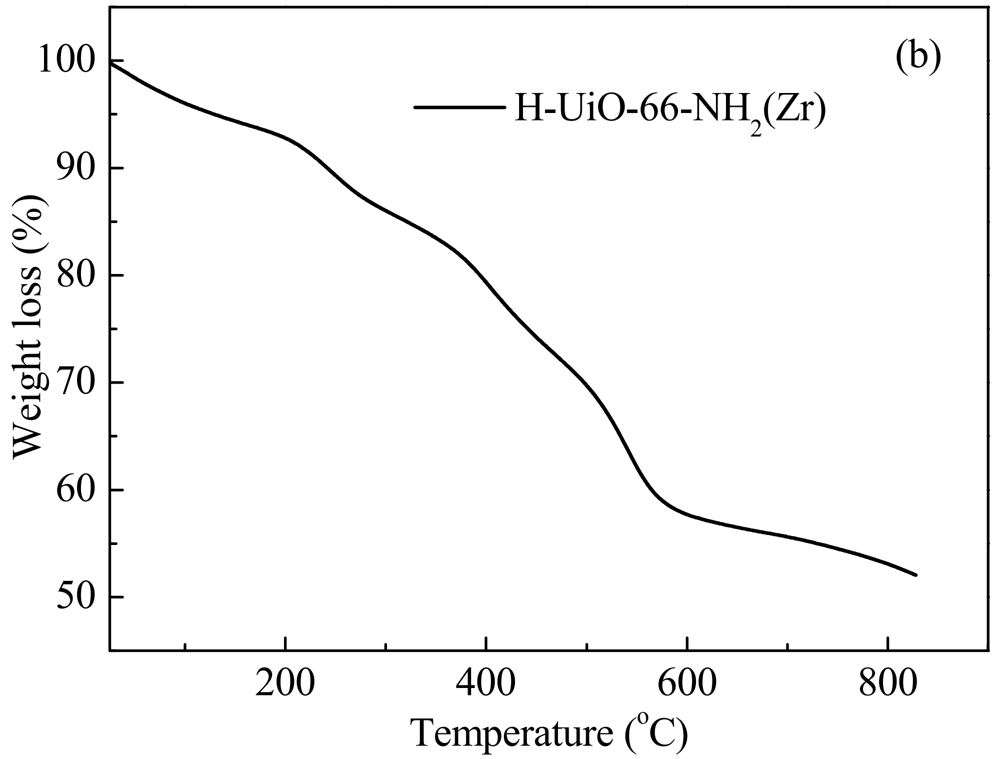
(b)
H-UiO-66-NH2(Zr)
Weight loss (%)
100
90
80
70
60
50
200
400
600
800
Temperature (°C)

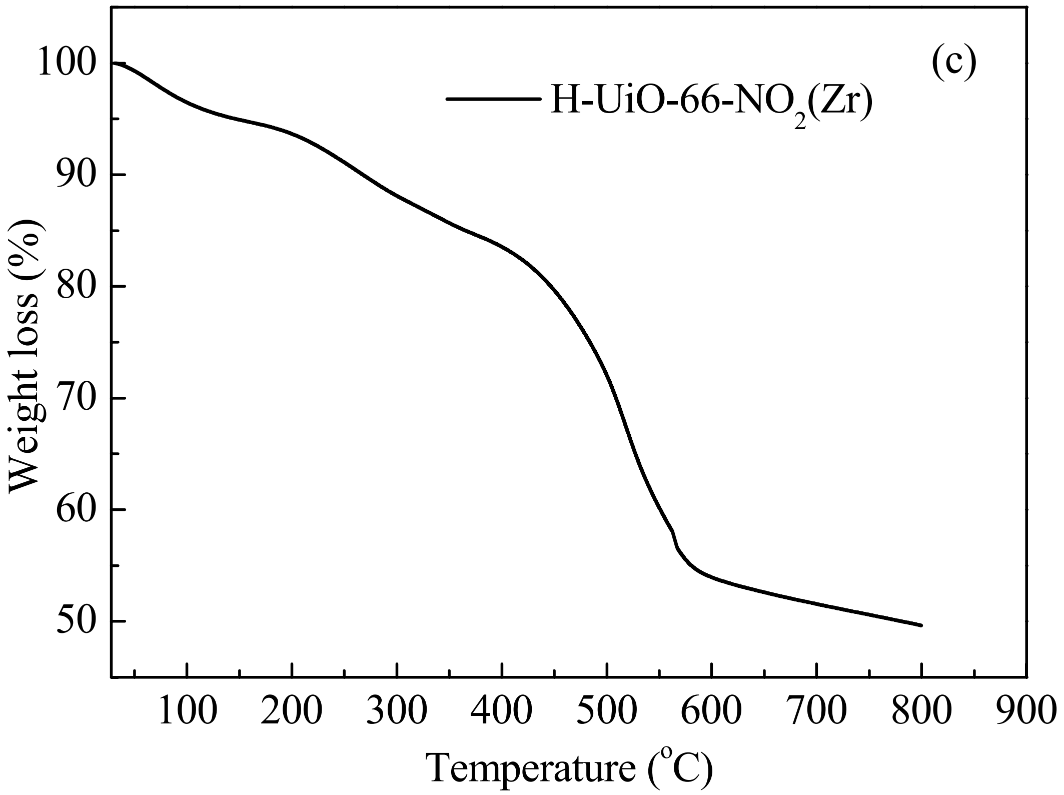
(c)
H-UiO-66-NO2(Zr)
Weight loss (%)
100
90
80
70
60
50
100
200
300
400
500
600
700
800
900
Temperature (°C)

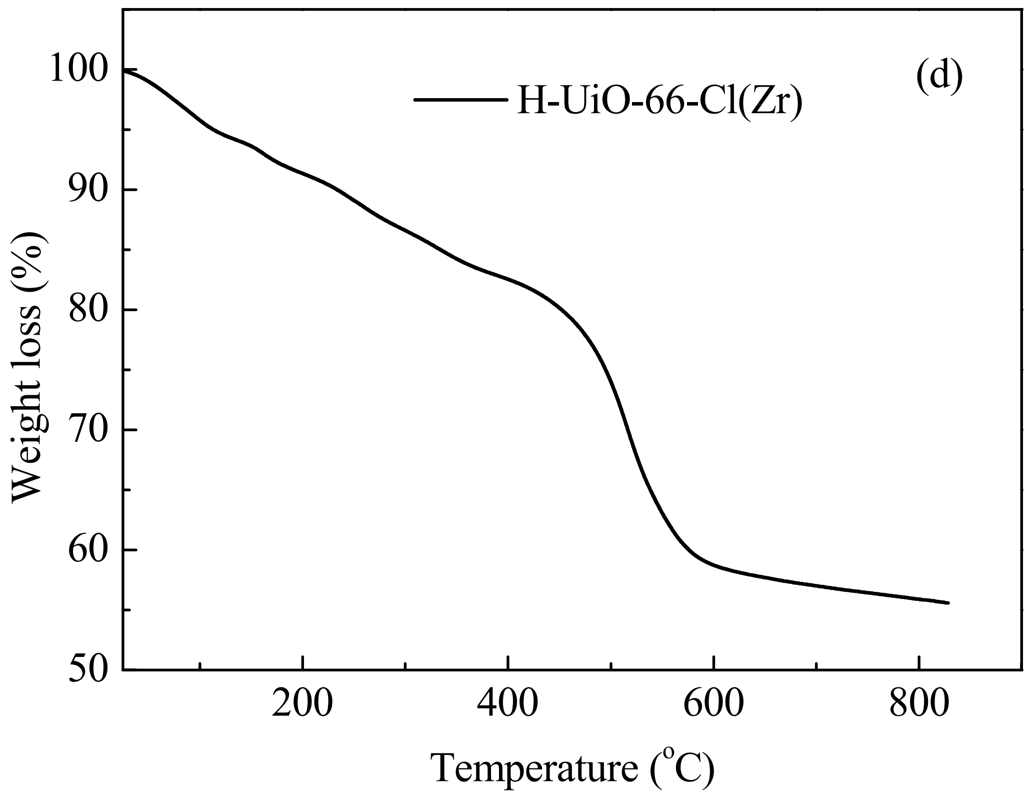
(d)
H-UiO-66-Cl(Zr)
Weight loss (%)
100
90
80
70
60
50
200
400
600
800
Temperature (°C)

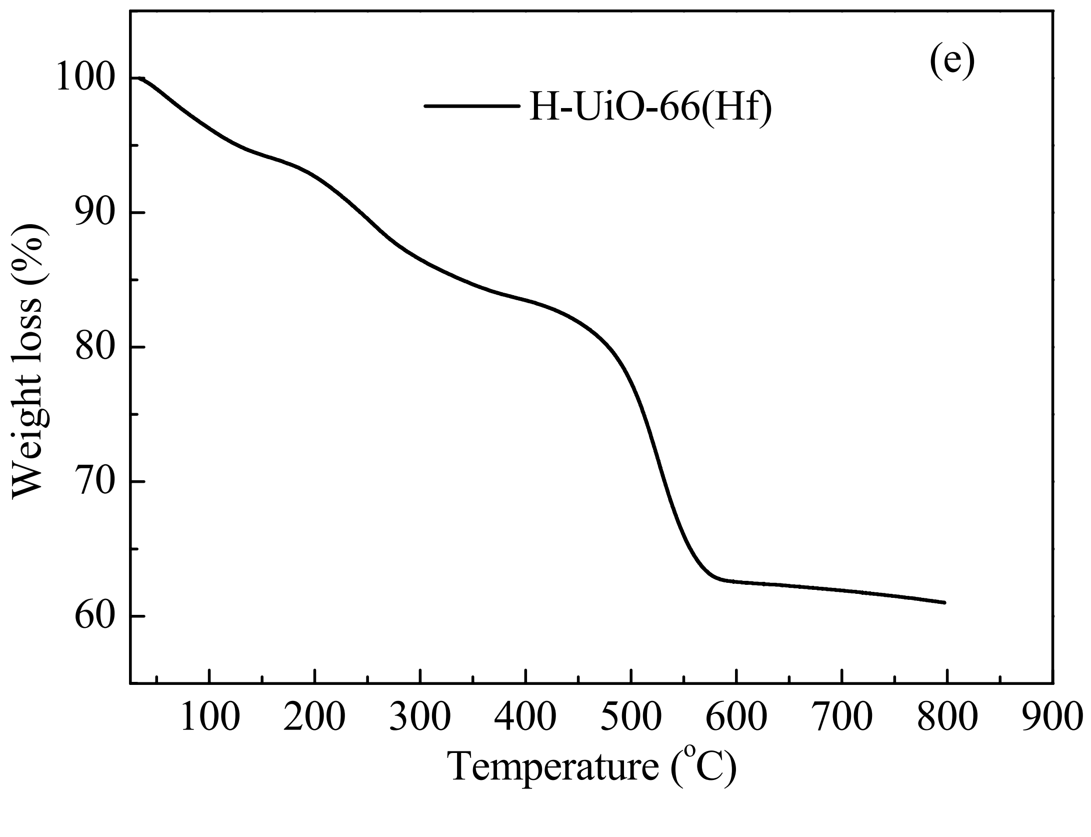
(e)
H-UiO-66(Hf)
Weight loss (%)
100
90
80
70
60
100
200
300
400
500
600
700
800
900
Temperature (°C)

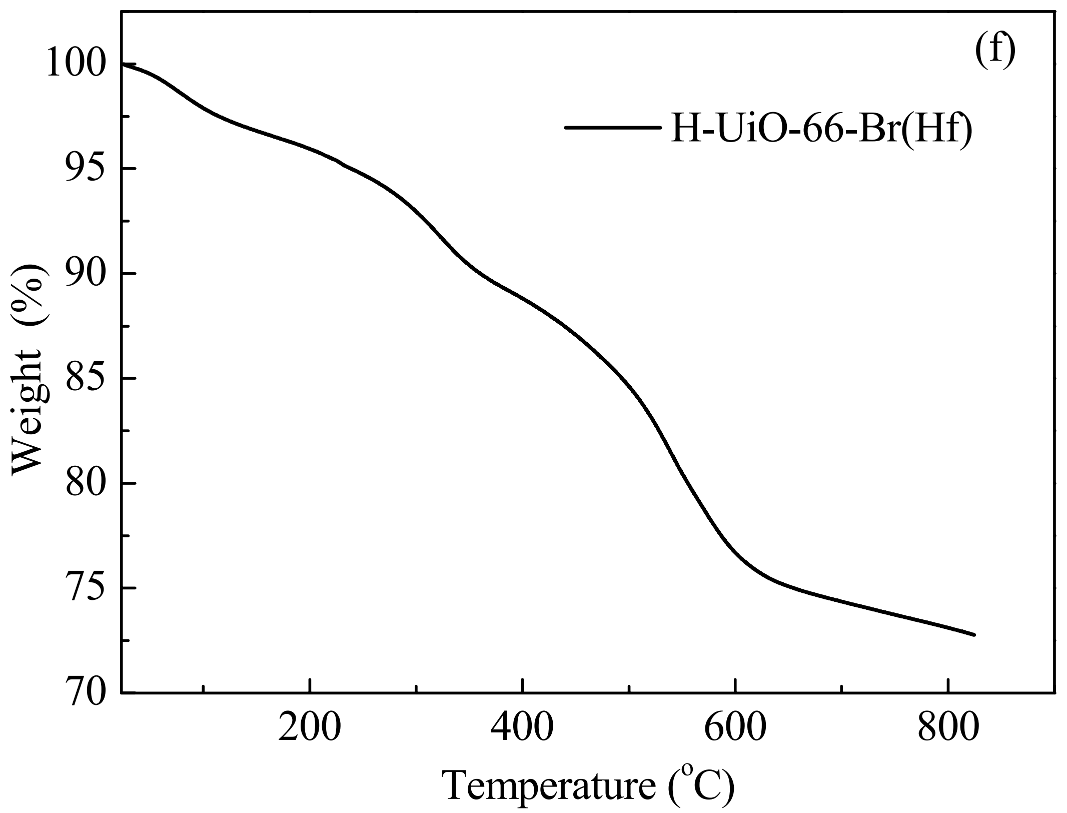
(f)
H-UiO-66-Br(Hf)
Weight (%)
100
95
90
85
80
75
70
200
400
600
800
Temperature (°C)

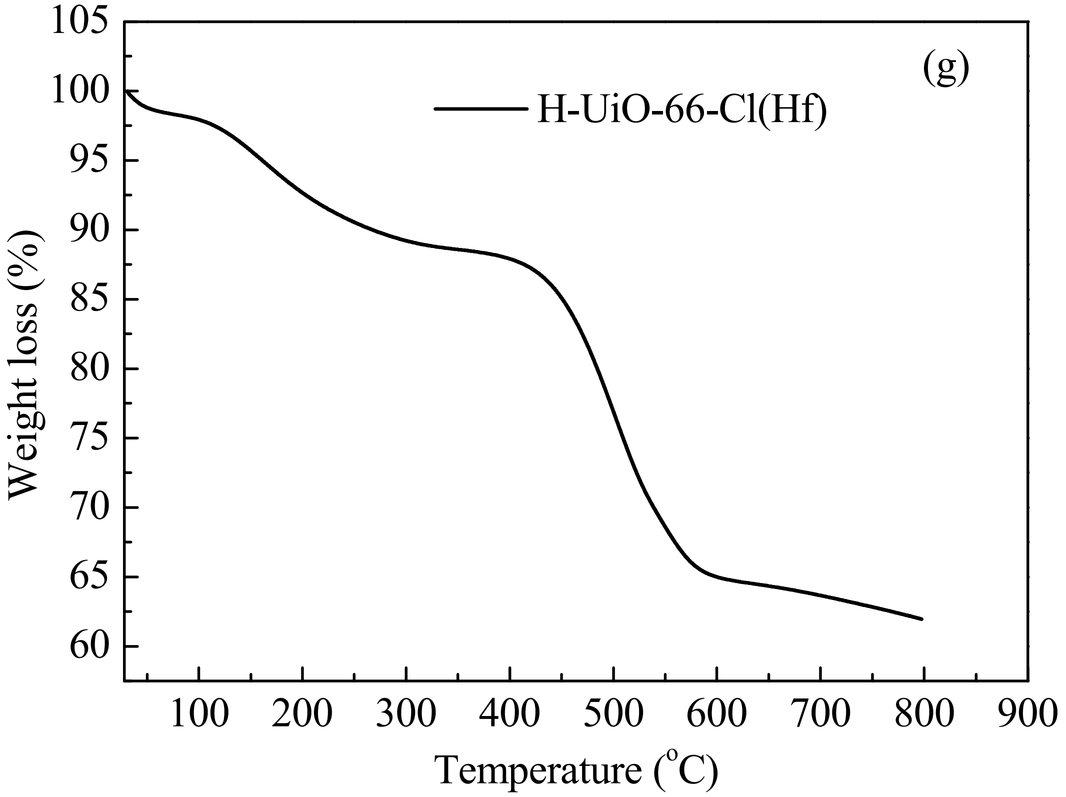
(g)
H-UiO-66-Cl(Hf)
Weight loss (%)
105
100
95
90
85
80
75
70
65
60
100
200
300
400
500
600
700
800
900
Temperature (°C)

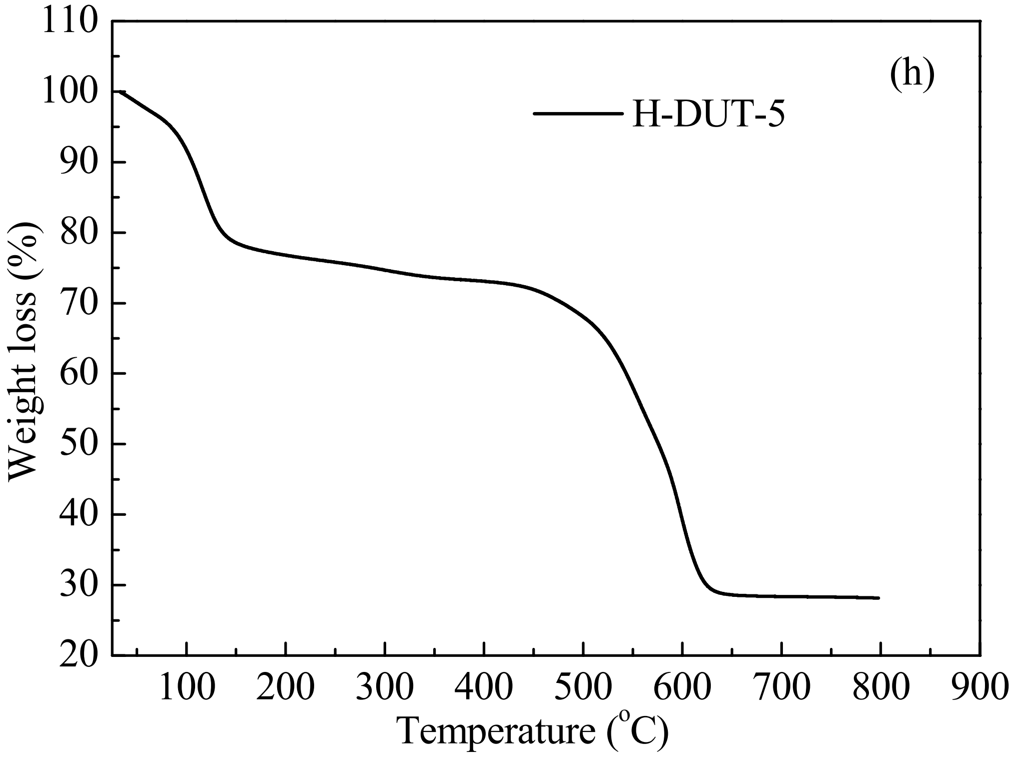

(h)
H-DUT-5
Weight loss (%)
Temperature (°C)
110
100
90
80
70
60
50
40
30
20
100
200
300
400
500
600
700
800
900

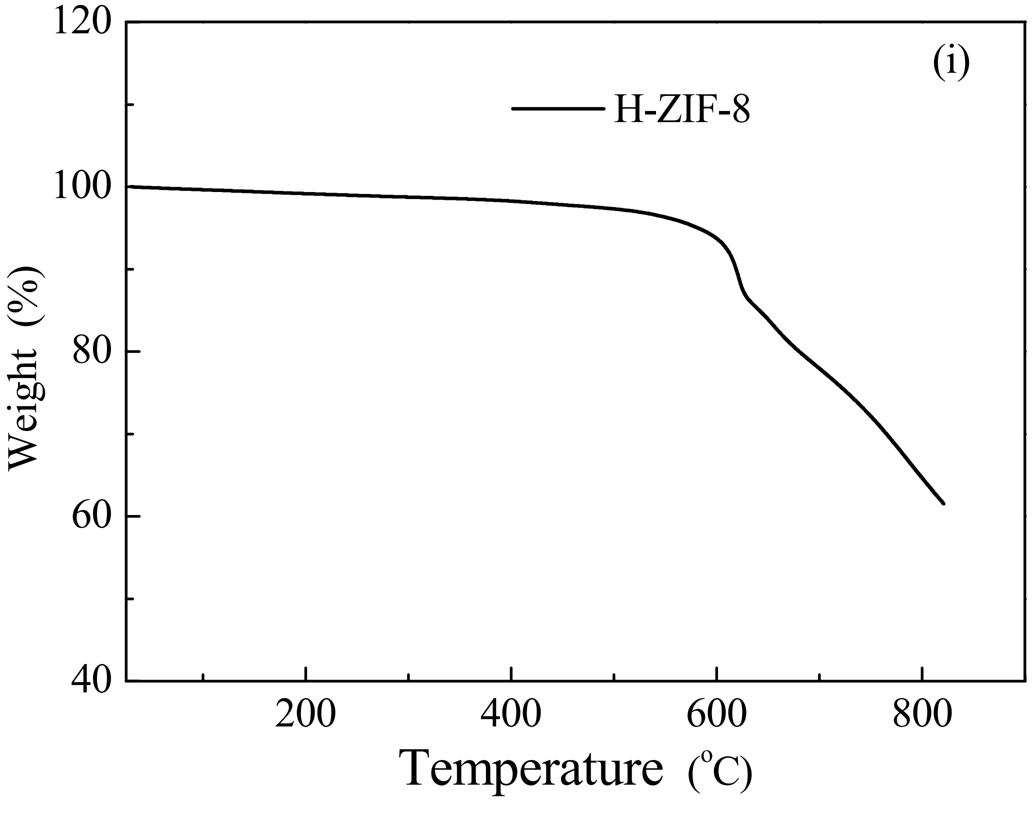

(i)
H-ZIF-8
Weight (%)
Temperature (°C)
120
100
80
60
40
200
400
600
800

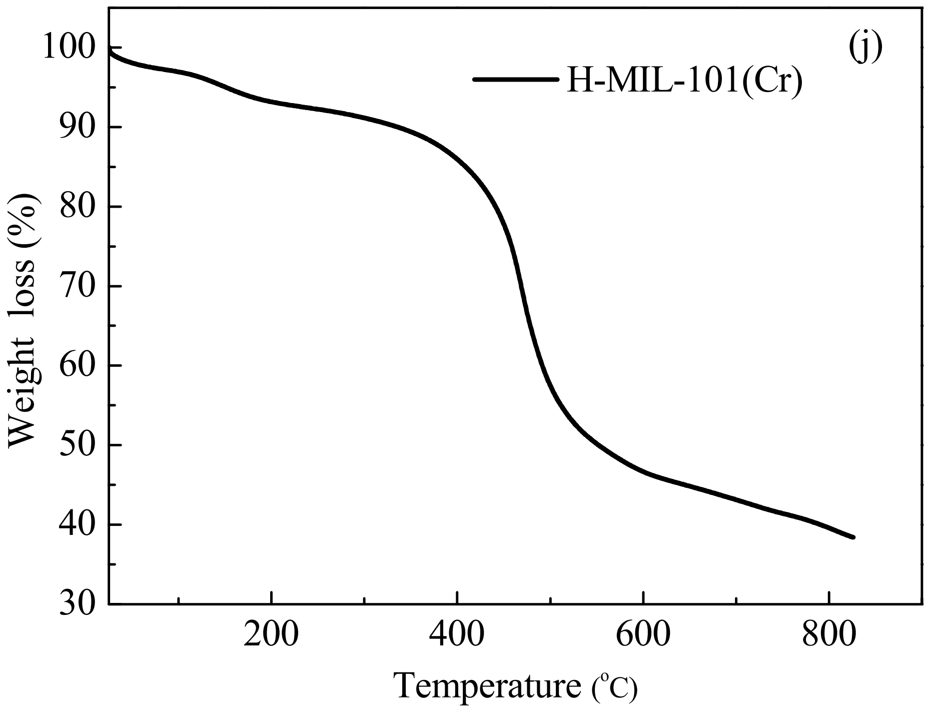

Supplementary Figure 29. TGA curves of H-MOFs synthesized using different MOF precursors templates by one-pot reaction method. (**a**, **e**, **i**) MOF-5 precursors, (**b**) IRMOF-3 precursors, (**c**) MOF-5-NO_2 precursors, (**d**, **g**) MOF-5-Cl precursors, (**f**) MOF-5-Br precursors, (**h**) In-BPDC precursors, and (**j**) ZIF-8 precursors.

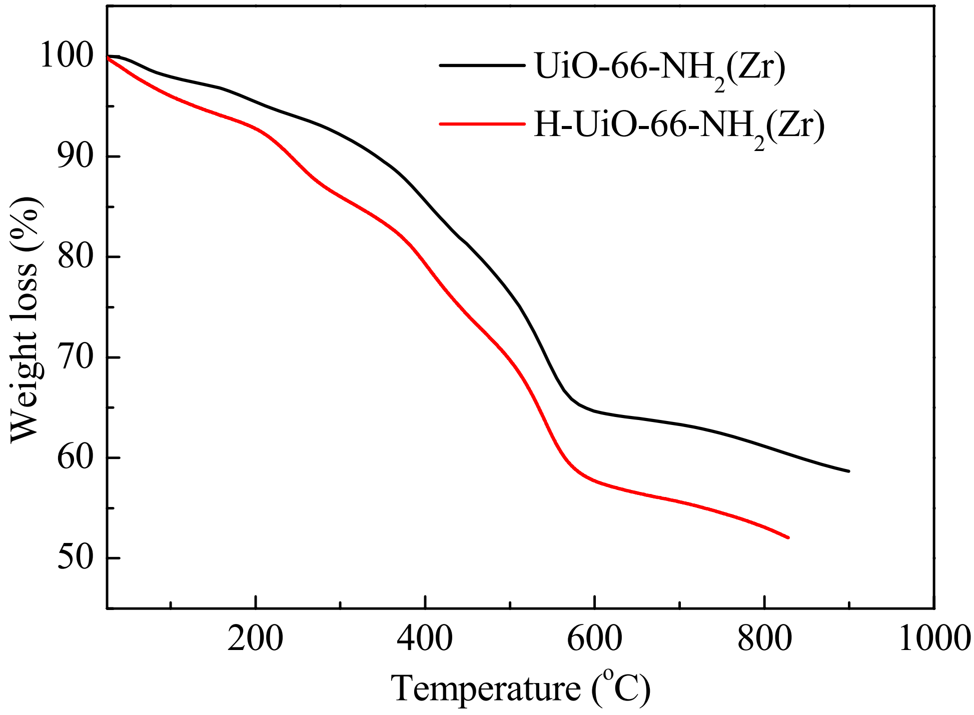

Supplementary Figure 30. TGA curves of H-UiO-66-NH_2(Zr) and UiO-66-NH_2(Zr). The continuous weight loss of H-UiO-66-NH_2(Zr) and UiO-66-NH_2(Zr) in a broad temperature range may be ascribed to the introduction of polar group in these MOFs. This phenomenon has also been observed in other functionalized MOFs[1,2], including UiO-66-NH_2(Zr). DeCoste *et al.* indeed have demonstrated that the ability of the nitrogen atoms in the ligand to withdraw electrons inductively weakens the neighboring carbon-carbon bonds in benzene ring, causing thermal breakdown of the UiO-66-NH_2(Zr) at lower temperatures[3]. This was combined by the coordinated water removal, thereby leading to a continuous weight loss as observed.

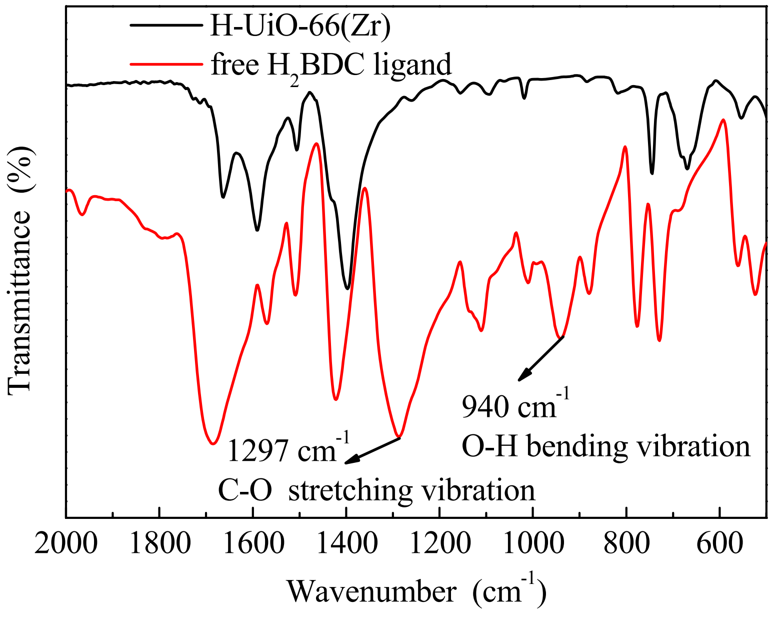

Supplementary Figure 31. FT-IR spectra of free H_2BDC ligand and H-UiO-66(Zr) synthesized using MOF-5 as the template precursors. The disappear of C-O stretching vibration at 1297 cm^{-1} and O-H bending vibration at 940 cm^{-1} indicates no free H_2BDC remained in the pores of H-UiO-66(Zr).

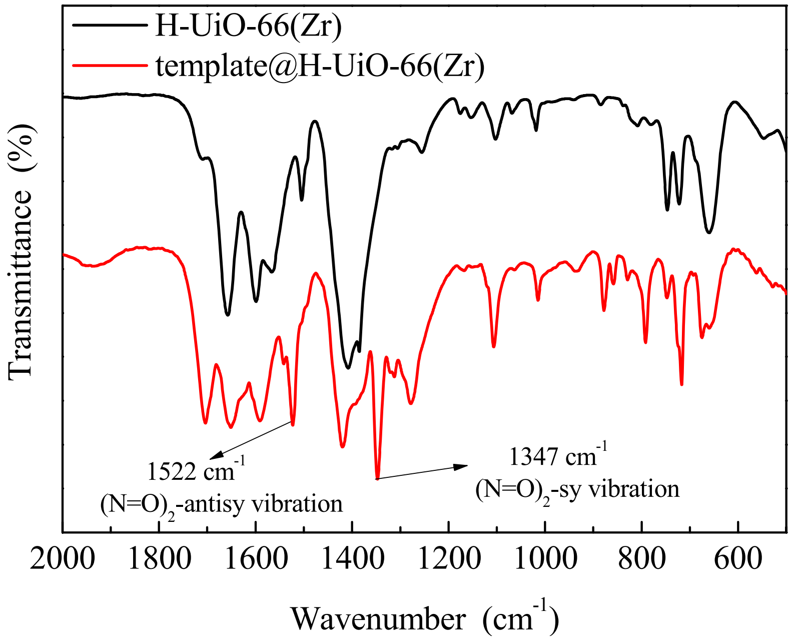

Supplementary Figure 32. FT-IR spectra of H-UiO-66(Zr) and template@H-UiO-66(Zr) synthesized using $Zn_4O(BC\text{-}NO_2)_6$ as the template precursors templates by two-step reaction method.

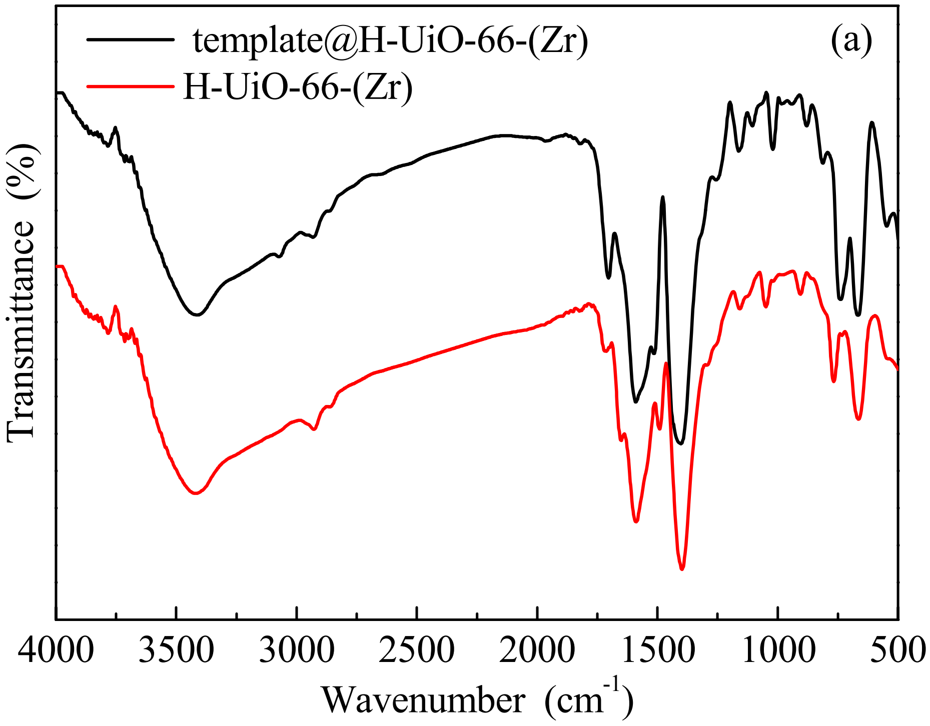
template@H-UiO-66-(Zr)
H-UiO-66-(Zr)
(a)
Transmittance (%)
4000
3500
3000
2500
2000
1500
1000
500
Wavenumber (cm-1)

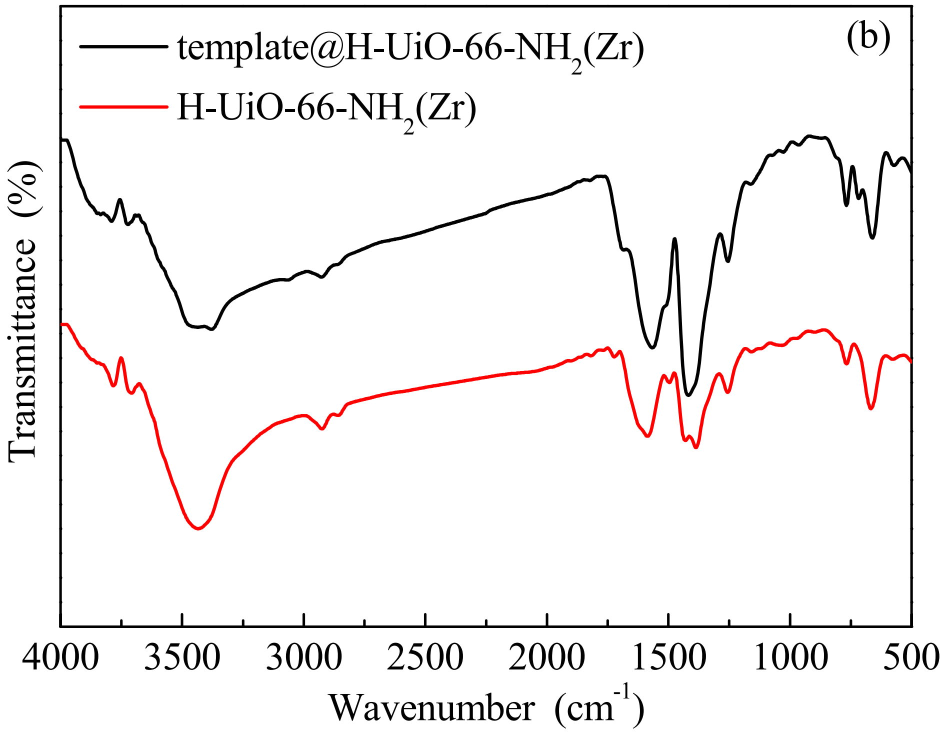
template@H-UiO-66-NH2(Zr)
H-UiO-66-NH2(Zr)
(b)
Transmittance (%)
4000
3500
3000
2500
2000
1500
1000
500
Wavenumber (cm-1)

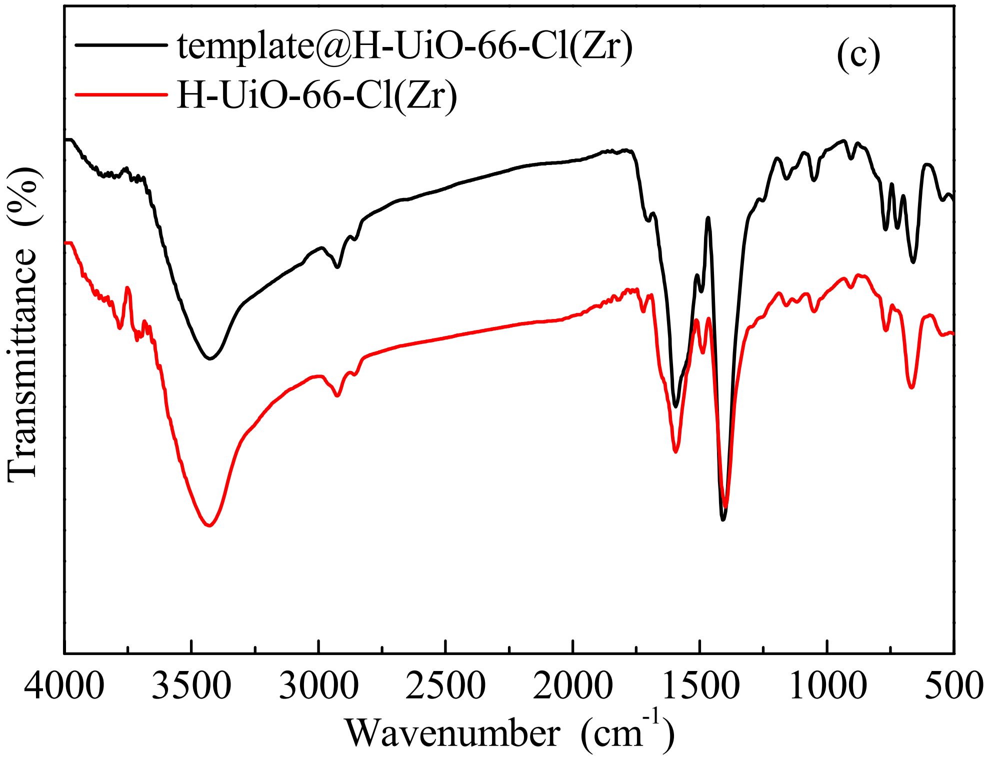

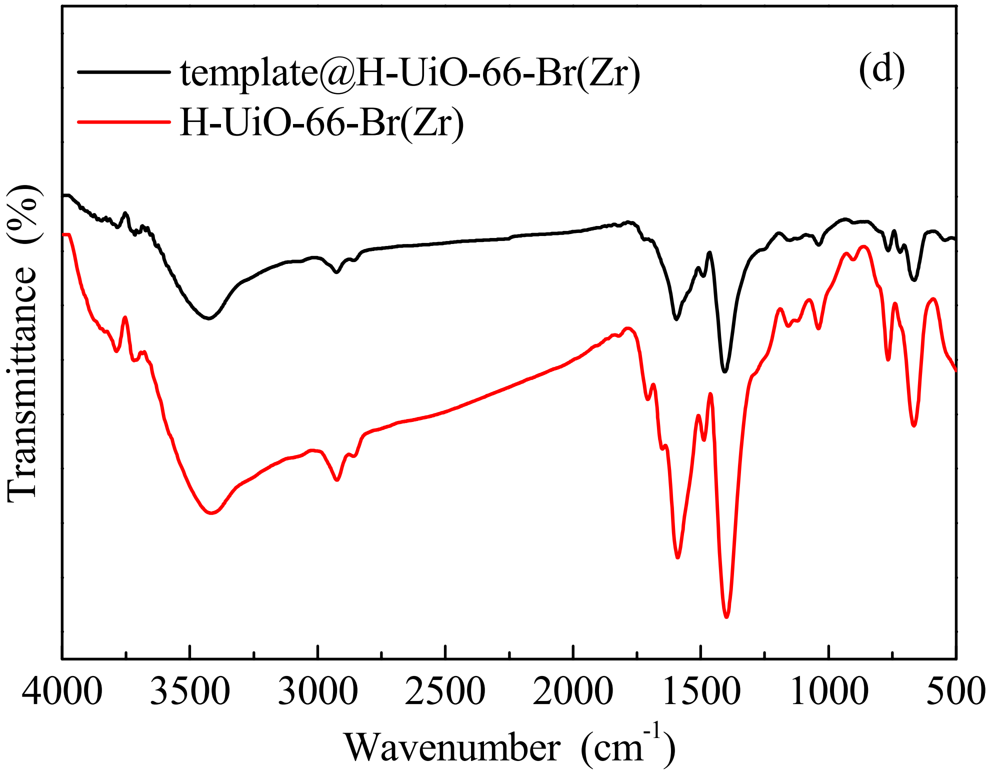

Supplementary Figure 33. FT-IR spectra of template@H-MOFs and H-MOFs synthesized using $Zn_4O(BC)_6$ precursors by one-pot reaction method.

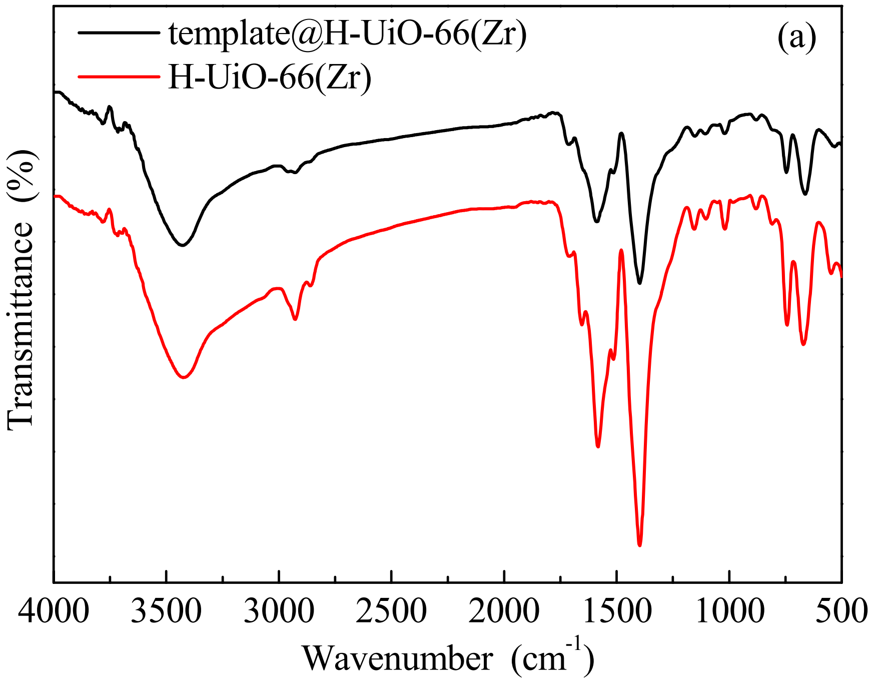
template@H-UiO-66(Zr)
H-UiO-66(Zr)
(a)
Transmittance (%)
4000
3500
3000
2500
2000
1500
1000
500
Wavenumber (cm-1)

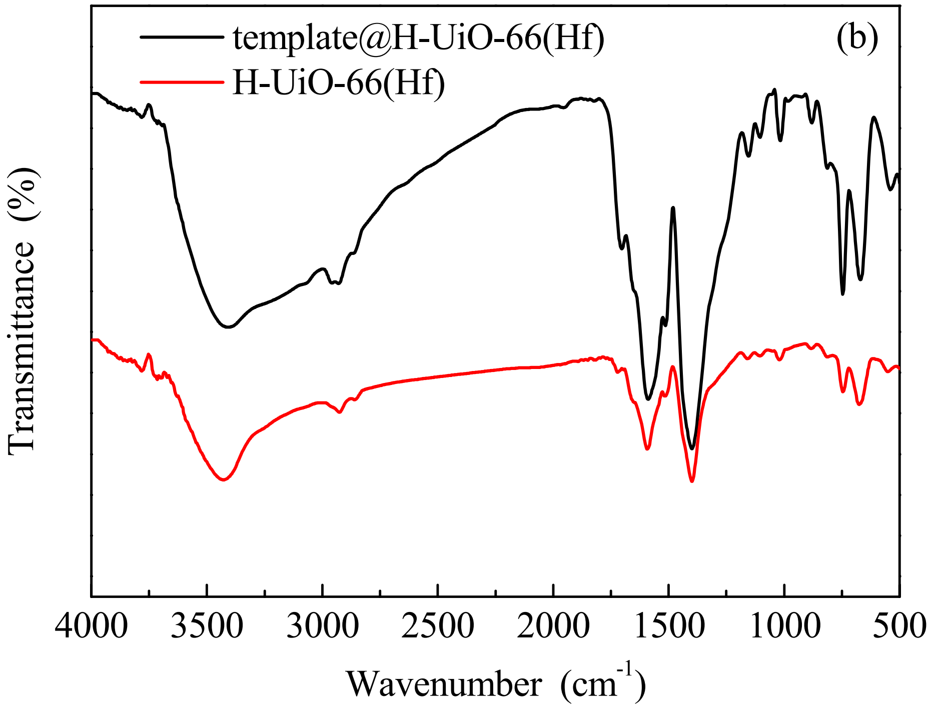
template@H-UiO-66(Hf)
H-UiO-66(Hf)
(b)
Transmittance (%)
4000
3500
3000
2500
2000
1500
1000
500
Wavenumber (cm-1)

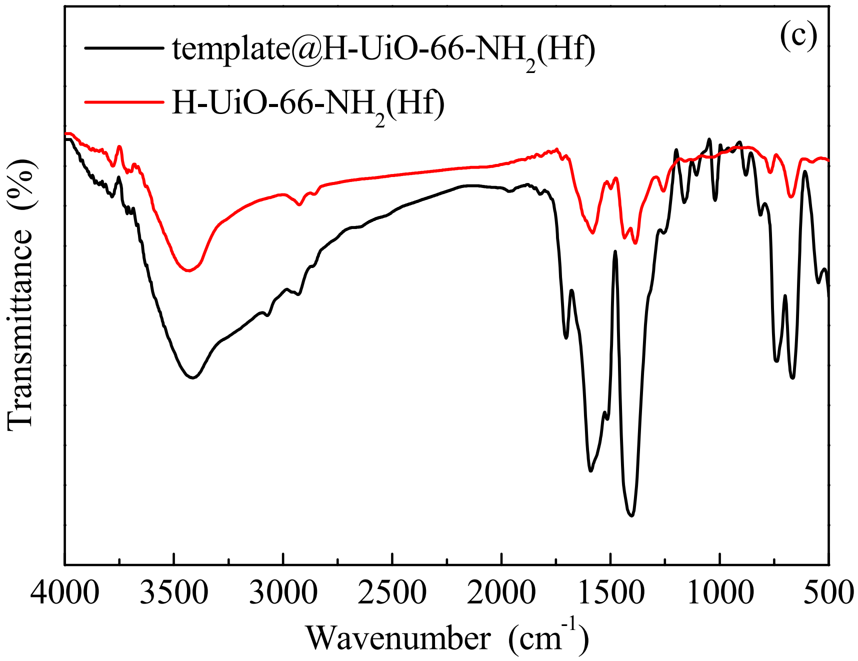
template@H-UiO-66-NH2(Hf)
H-UiO-66-NH2(Hf)
(c)
Transmittance (%)
4000
3500
3000
2500
2000
1500
1000
500
Wavenumber (cm-1)

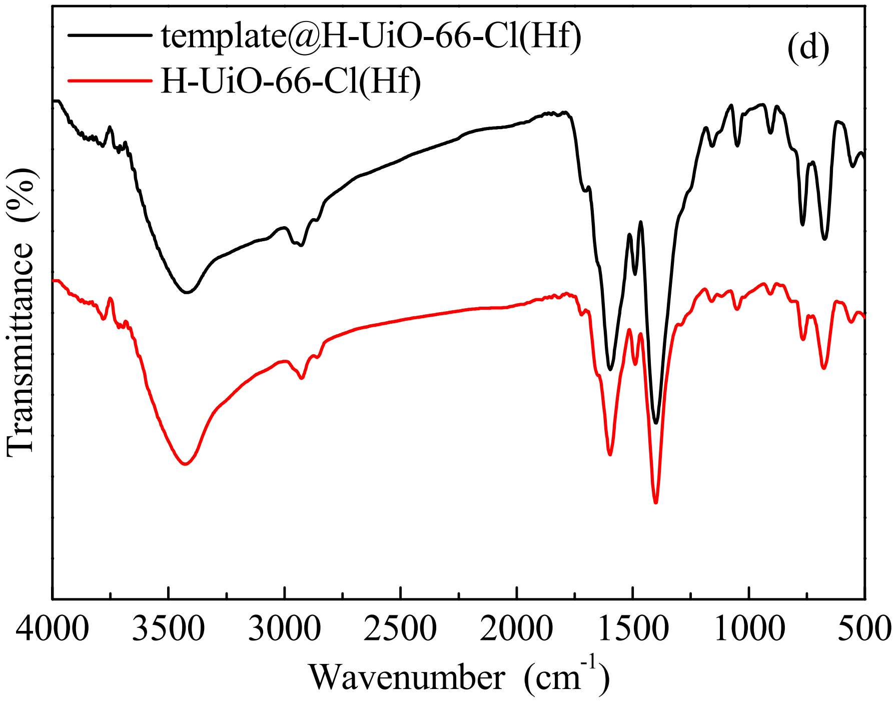
template@H-UiO-66-Cl(Hf)
H-UiO-66-Cl(Hf)
(d)
Transmittance (%)
4000
3500
3000
2500
2000
1500
1000
500
Wavenumber (cm-1)

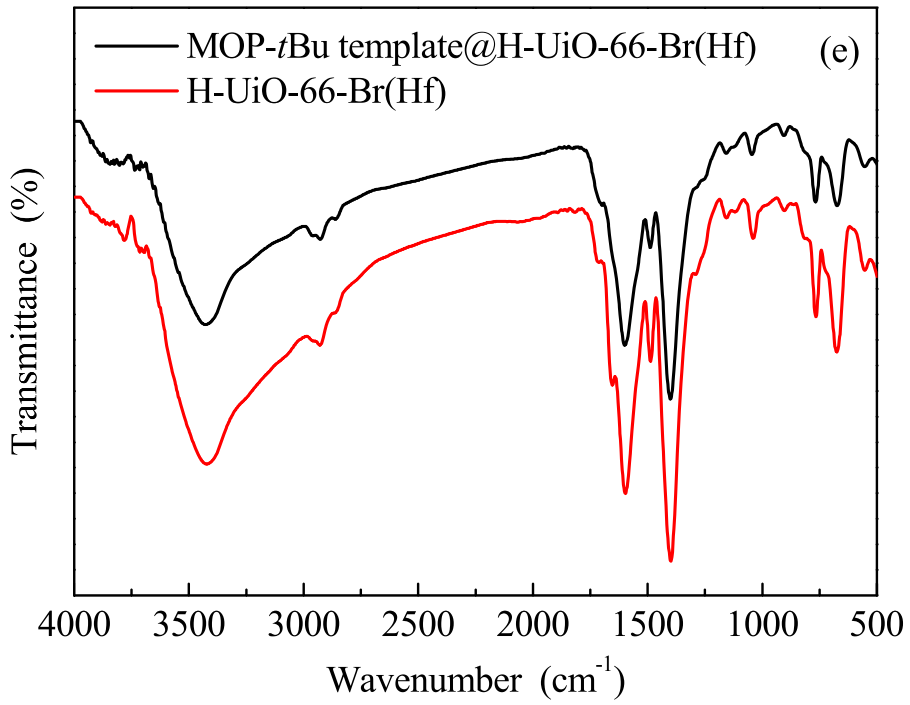

Supplementary Figure 34. FT-IR spectra of template@H-UiO-66-X and H-UiO-66-X synthesized using MOP-*t*Bu precursors by one-pot reaction method.

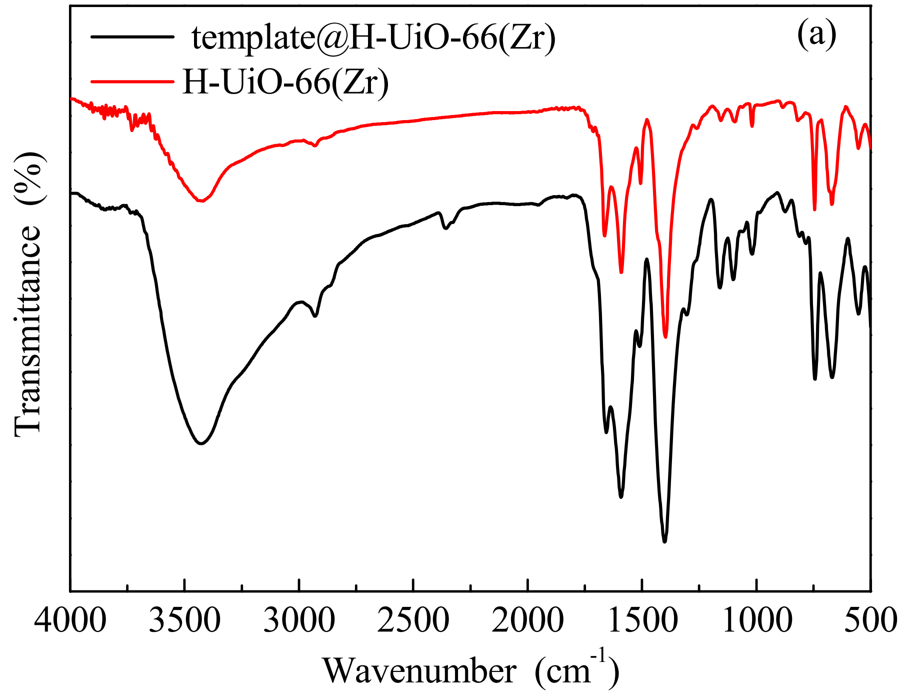

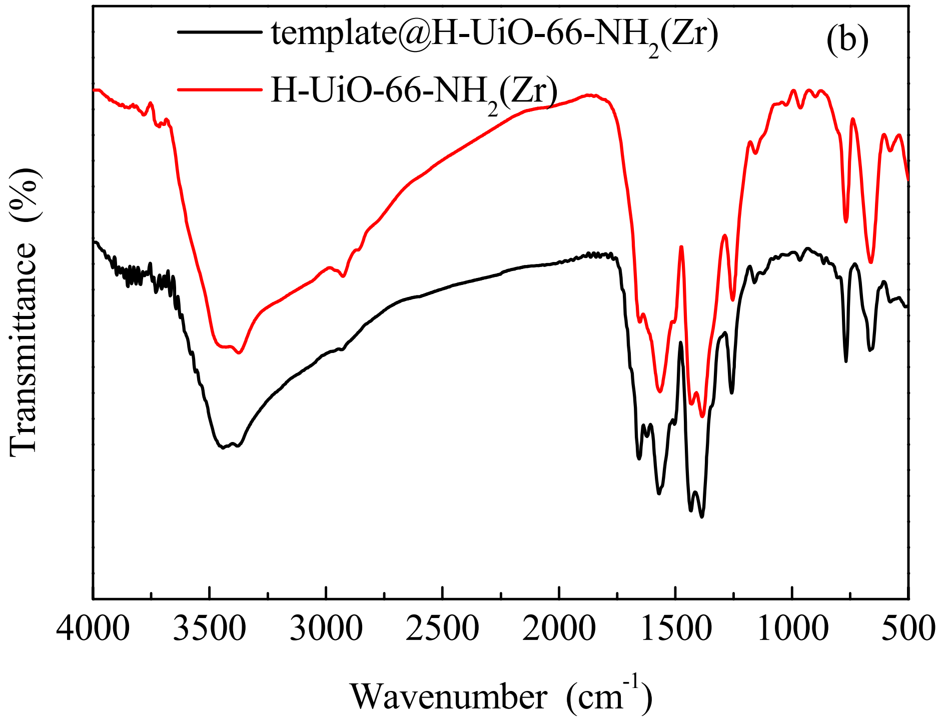

template@H-UiO-66-NH2(Zr)
H-UiO-66-NH2(Zr)
(b)
Transmittance (%)
4000
3500
3000
2500
2000
1500
1000
500
Wavenumber (cm-1)

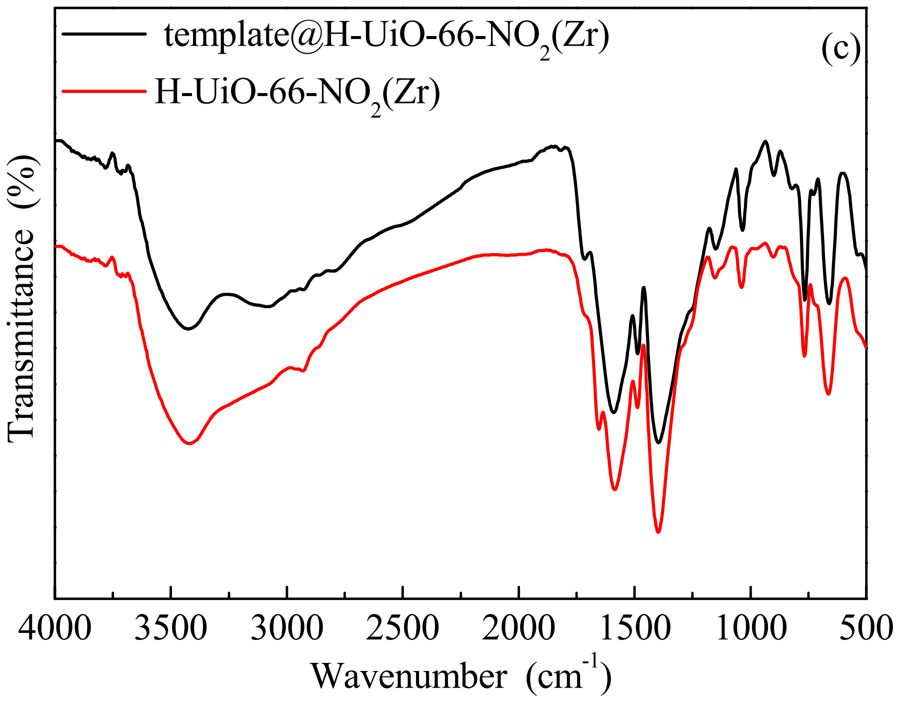

template@H-UiO-66-NO2(Zr)
H-UiO-66-NO2(Zr)
(c)
Transmittance (%)
4000
3500
3000
2500
2000
1500
1000
500
Wavenumber (cm-1)

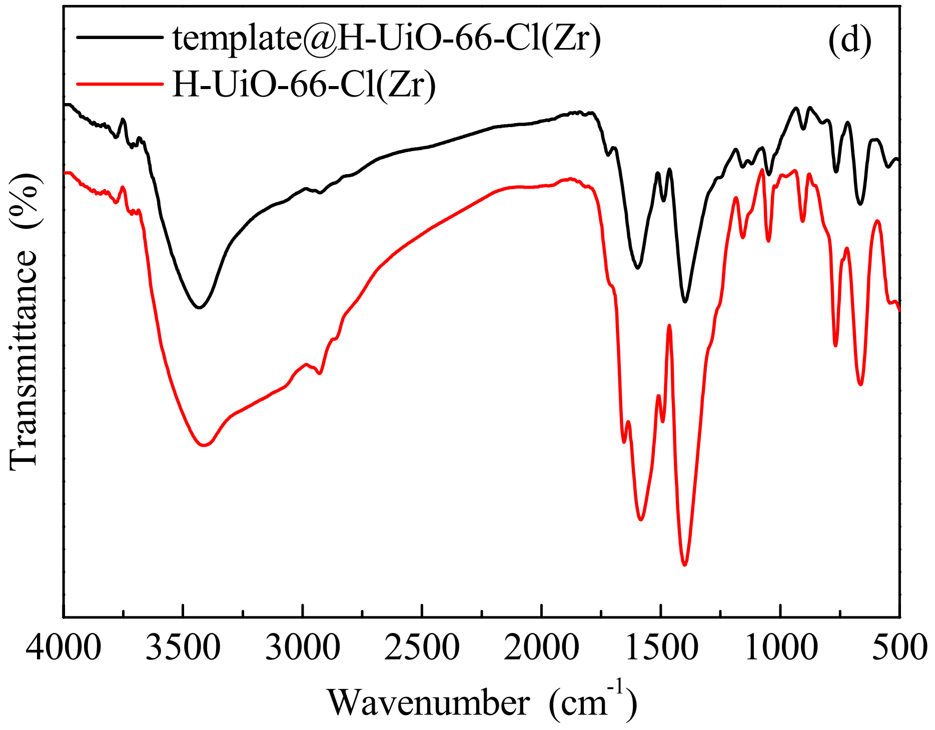

template@H-UiO-66-Cl(Zr)
H-UiO-66-Cl(Zr)
(d)
Transmittance (%)
4000
3500
3000
2500
2000
1500
1000
500
Wavenumber (cm-1)

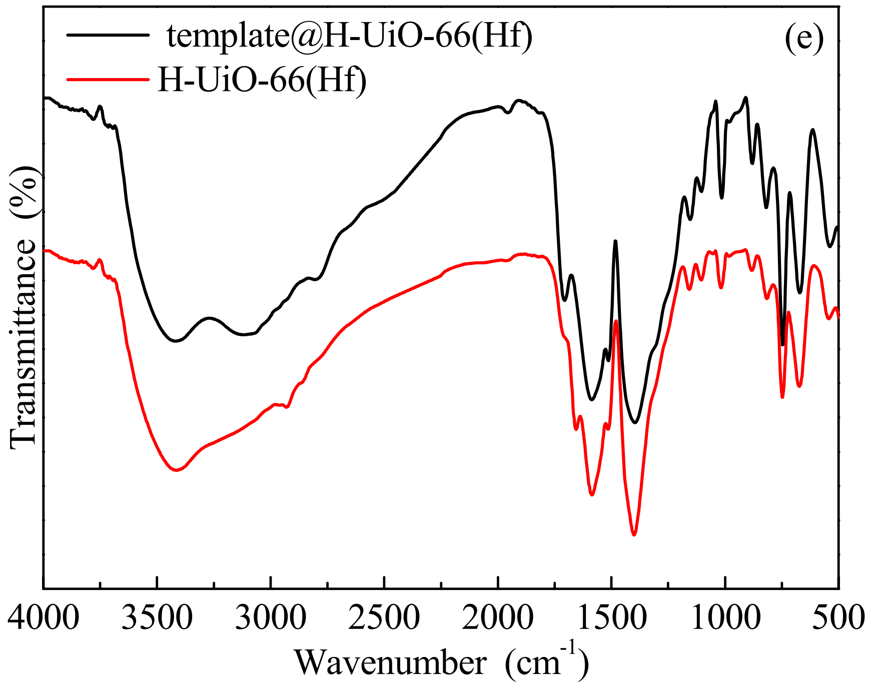

template@H-UiO-66(Hf)
H-UiO-66(Hf)
(e)
Transmittance (%)
4000
3500
3000
2500
2000
1500
1000
500
Wavenumber (cm-1)

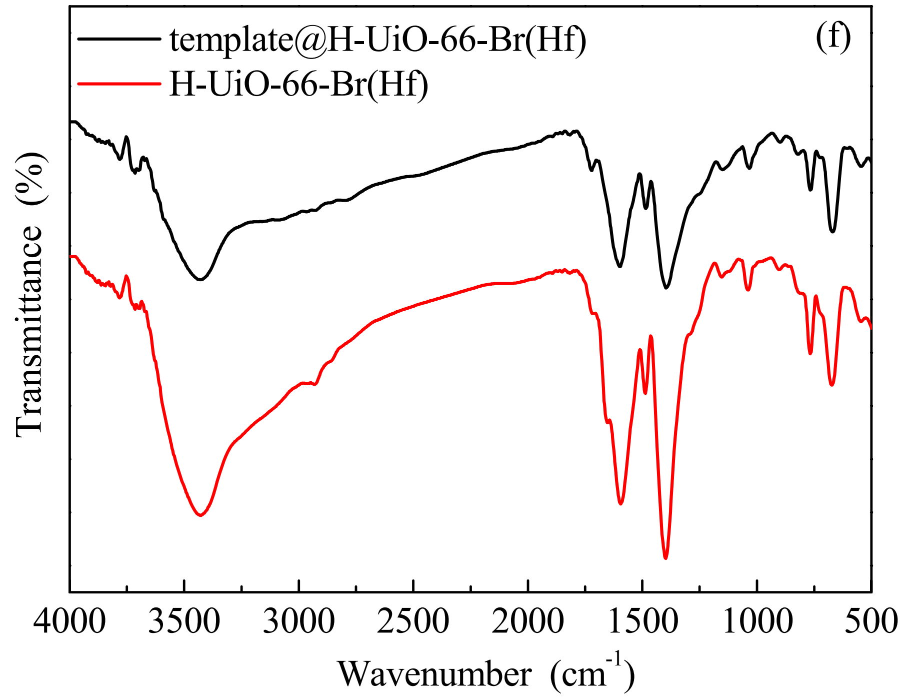
template@H-UiO-66-Br(Hf)
H-UiO-66-Br(Hf)
(f)
Transmittance (%)
4000
3500
3000
2500
2000
1500
1000
500
Wavenumber (cm-1)

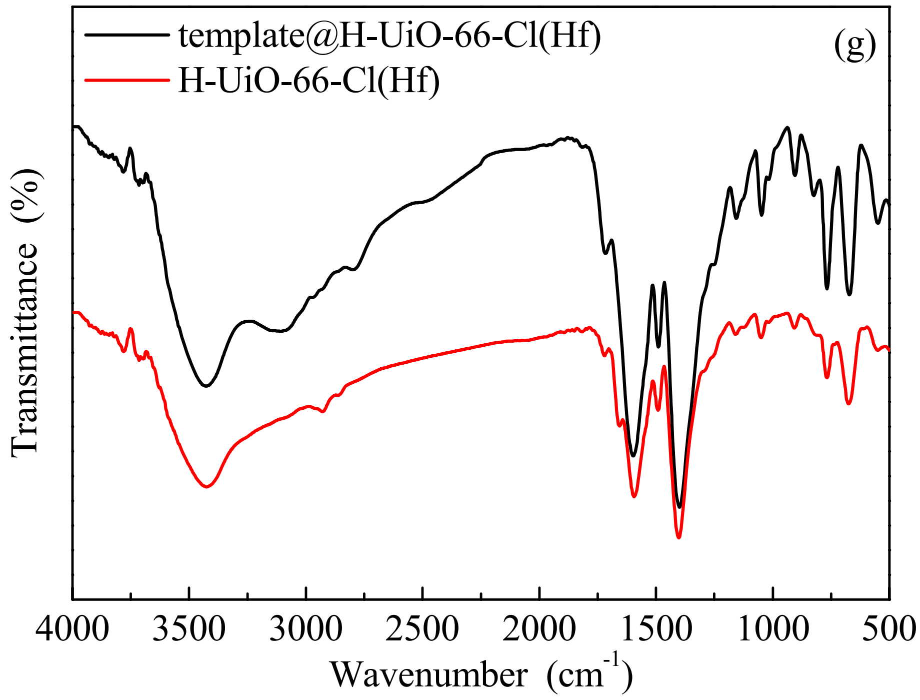
template@H-UiO-66-Cl(Hf)
H-UiO-66-Cl(Hf)
(g)
Transmittance (%)
4000
3500
3000
2500
2000
1500
1000
500
Wavenumber (cm-1)

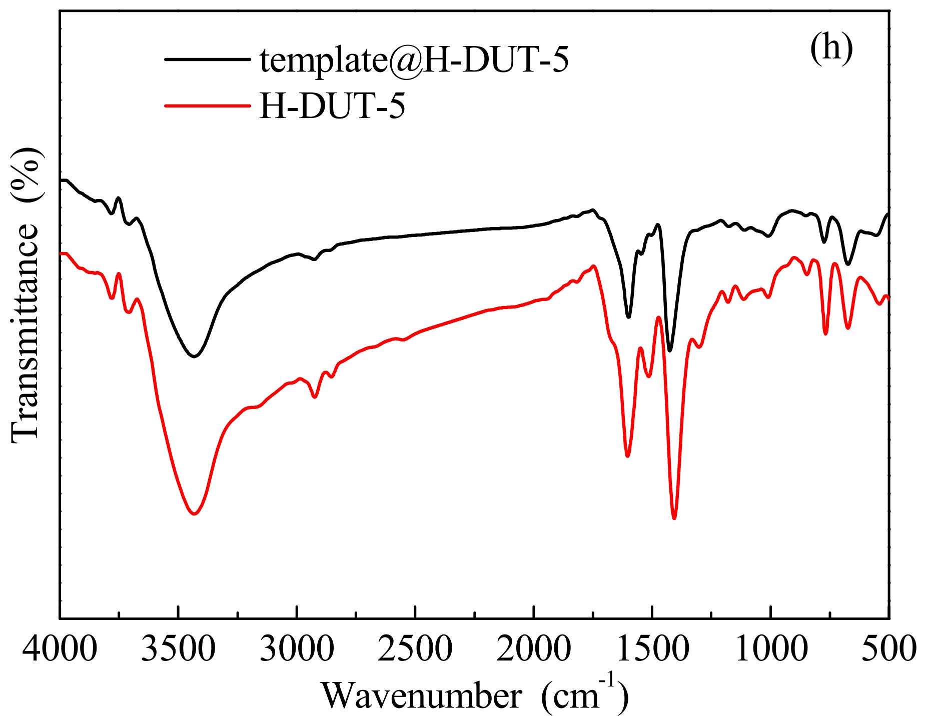
template@H-DUT-5
H-DUT-5
(h)
Transmittance (%)
4000
3500
3000
2500
2000
1500
1000
500
Wavenumber (cm-1)

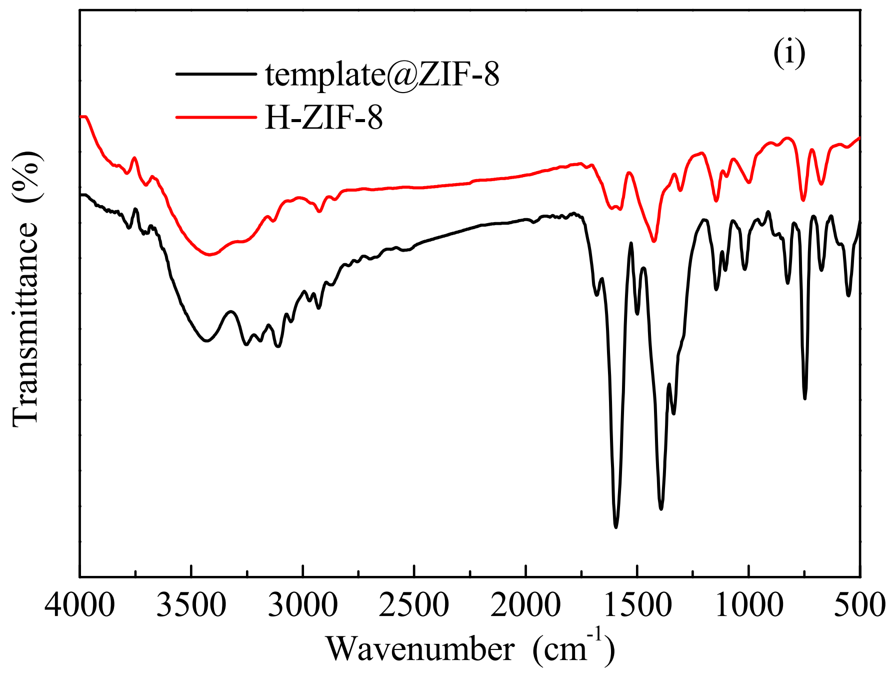
template@ZIF-8
H-ZIF-8
(i)
Transmittance (%)
4000
3500
3000
2500
2000
1500
1000
500
Wavenumber (cm-1)

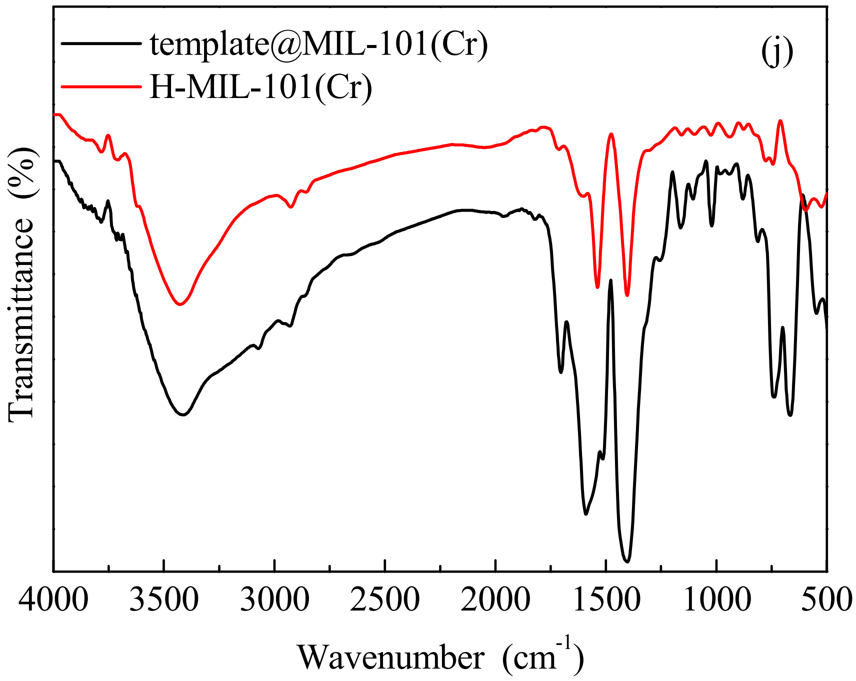

Supplementary Figure 35. FT-IR spectra of H-MOFs and template@H-MOFs synthesized using different MOF precursors by one-pot reaction method. (**a**, **e**, **i**) MOF-5 precursors, (**b**) IRMOF-3 precursors, (**c**) MOF-5-NO_2 precursors, (**d**, **g**) MOF-5-Cl precursors, (f) MOF-5-Br precursors, (**h**) In-BPDC precursors, and (**j**) ZIF-8 precursors.

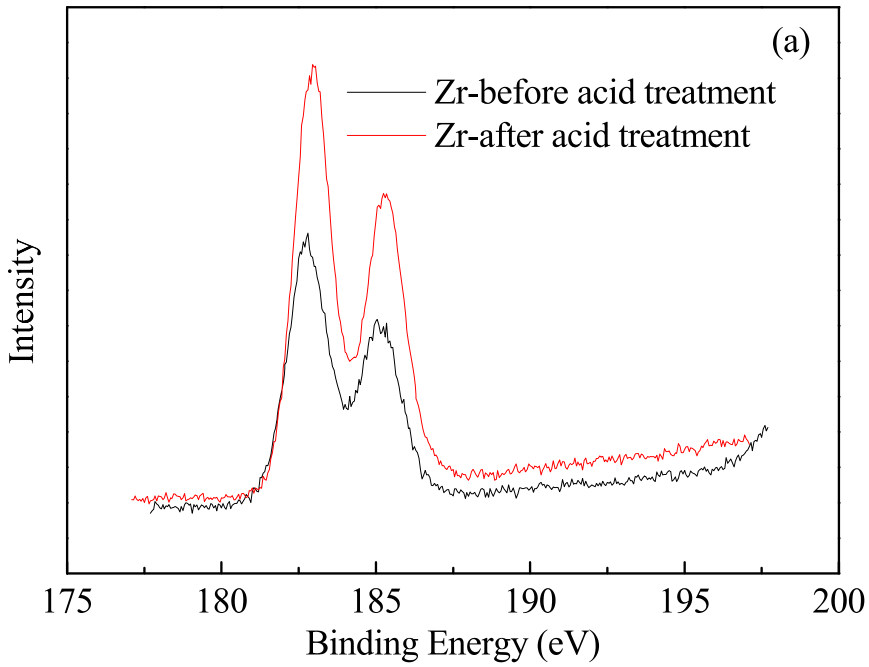

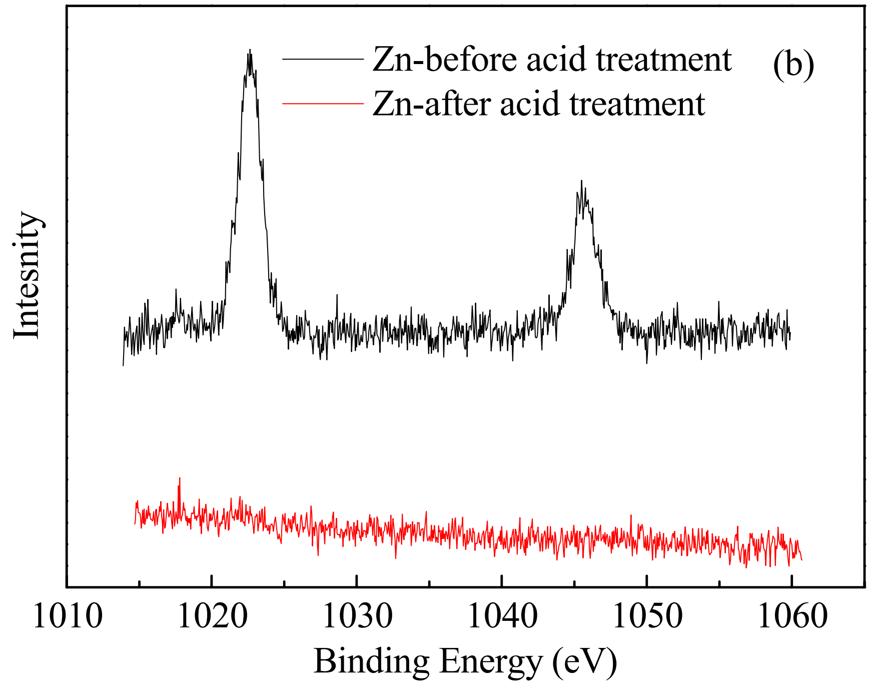

Supplementary Figure 36. The XPS spectra of H-UiO-66(Zr) prepared with $Zn_4O(BC)_6$ as template precursor by a two-step process before and after acid treatment over the Zr 3d (**a**) and Zn 2p (**b**) spectral region, respectively.

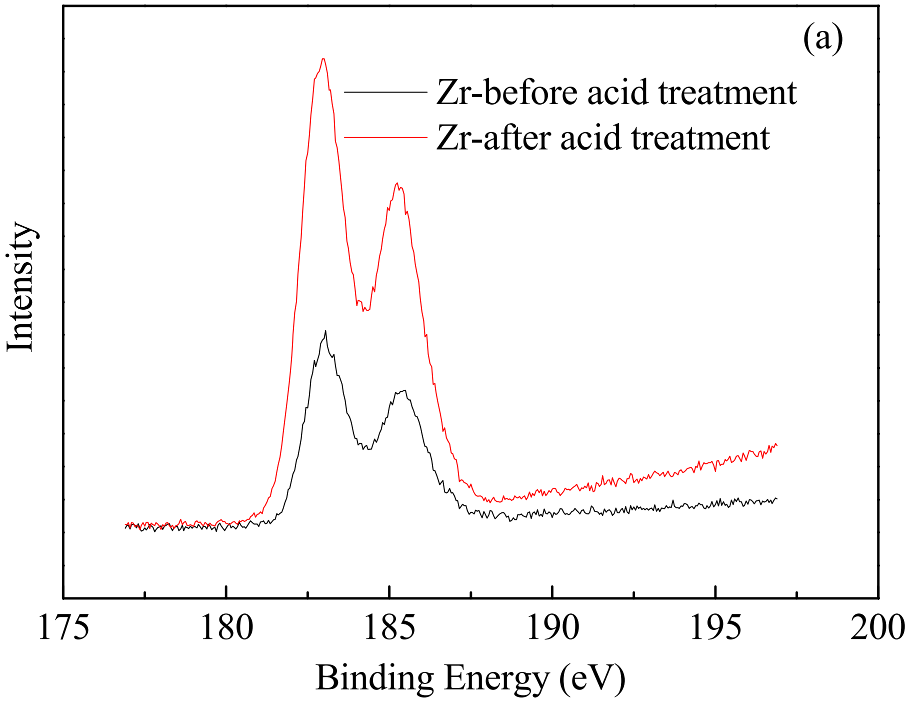

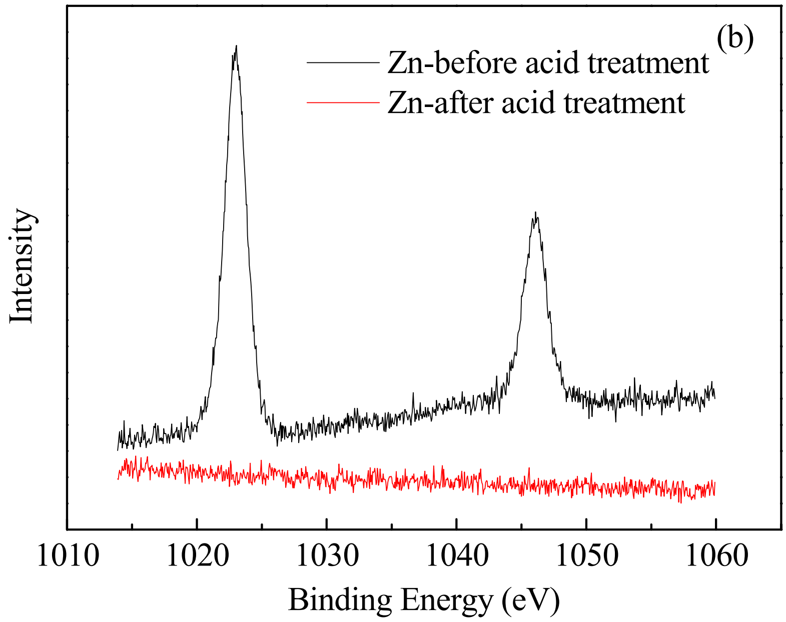

Supplementary Figure 37. The XPS spectra of H-UiO-66(Zr) prepared with MOF-5 as template precursor by a two-step process before and after acid treatment over the Zr 3d (**a**) and Zn 2p (**b**) spectral region, respectively.

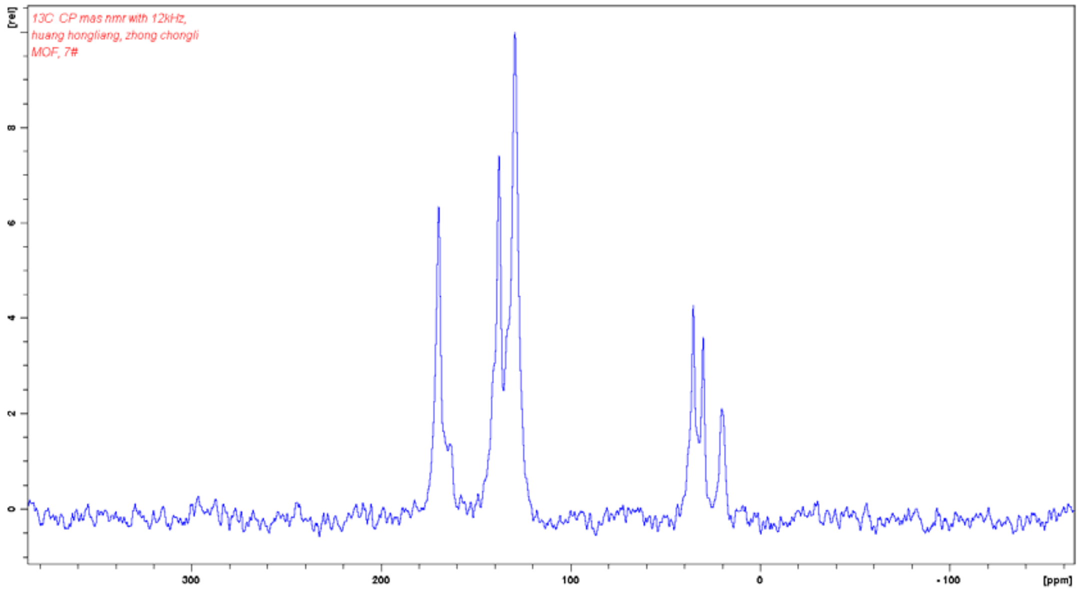

Supplementary Figure 38. ^{13}C-NMR of template@H-UiO-66(Zr) prepared by using $Zn_4O(BC\text{-}CH_3)_6$ acting as the template precursor. Chemical shift of methyl groups at 20 ppm in solid-state ^{13}C-NMR spectra indicates the existence of the $BC\text{-}CH_3$ in the $Zn_4O(BC\text{-}CH_3)_6$ template.

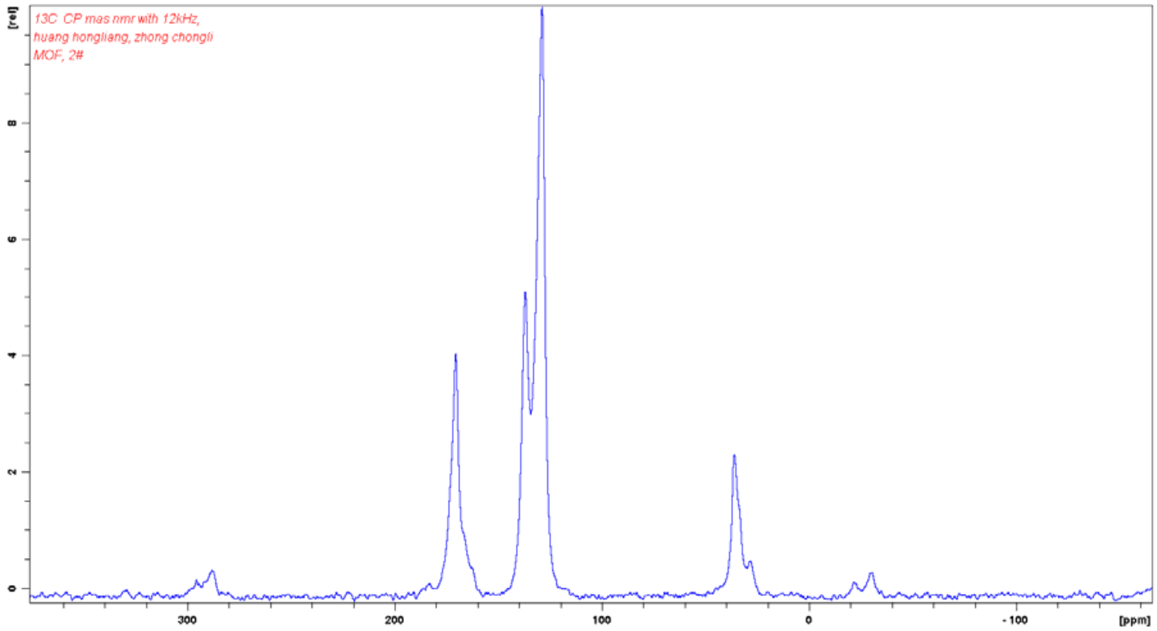

Supplementary Figure 39. ^{13}C-NMR of H-UiO-66(Zr) prepared by using $Zn_4O(BC\text{-}CH_3)_6$ as the template precursor.

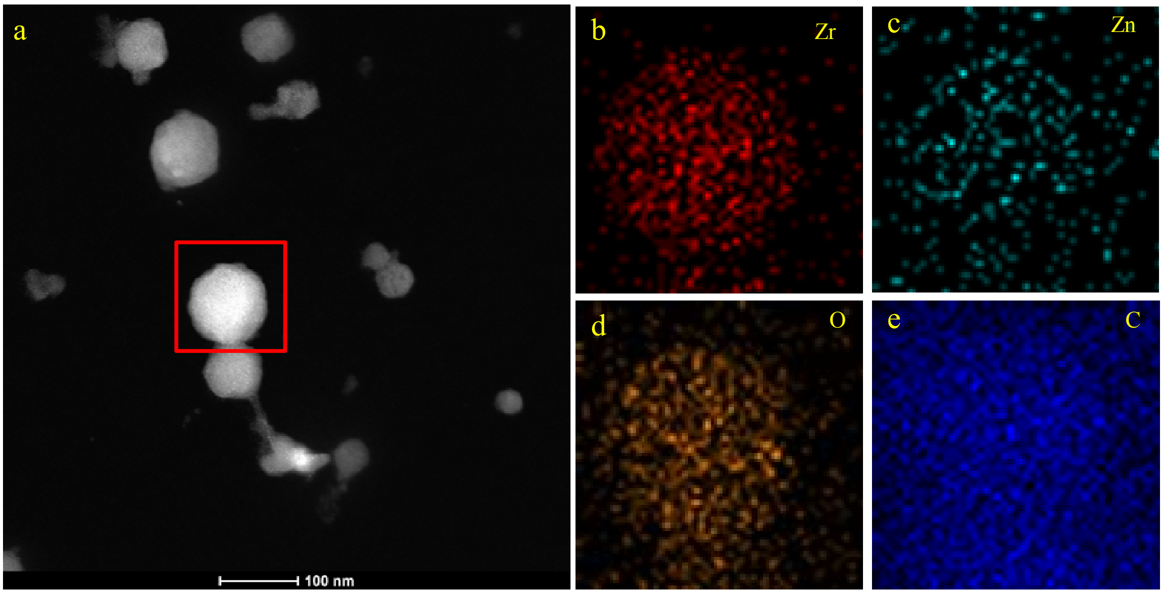

Supplementary Figure 40. STEM-HAADF image (**a**) and corresponding Zr (**b**), Zn (**c**), O (**d**), and C (**e**) elemental maps in template@H-UiO-66(Zr) prepared with MOF-5 as template precursor.

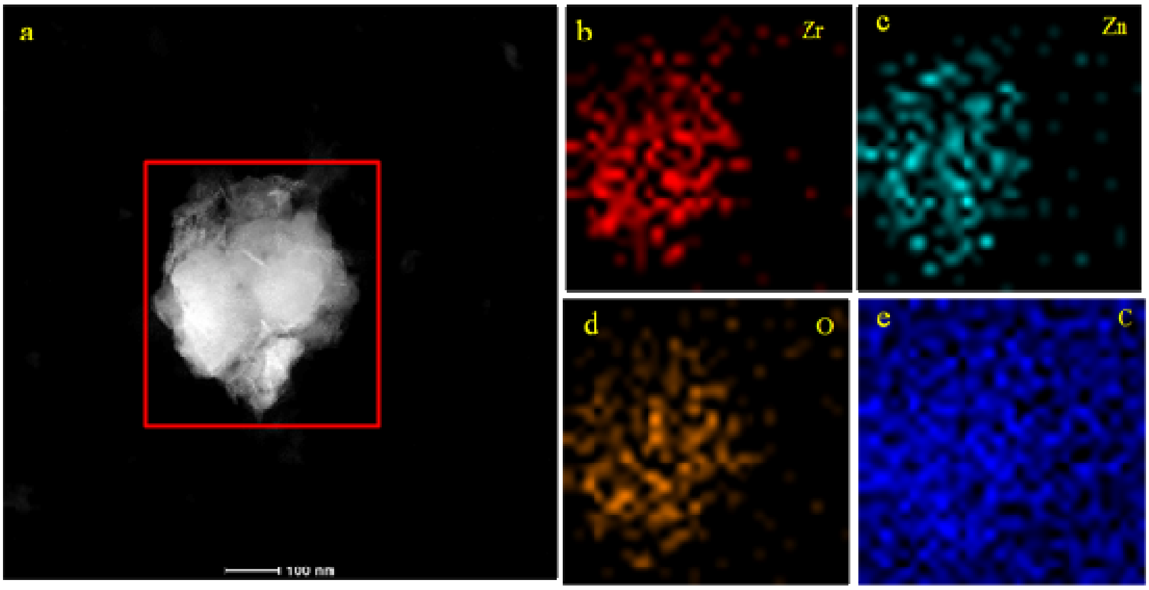

Supplementary Figure 41. STEM-HAADF image (**a**) and corresponding Zr (**b**), Zn (**c**), O (**d**), and C (**e**) elemental maps in template@H-UiO-66(Zr) prepared with $Zn_4O(BC)_6$ as template precursor.

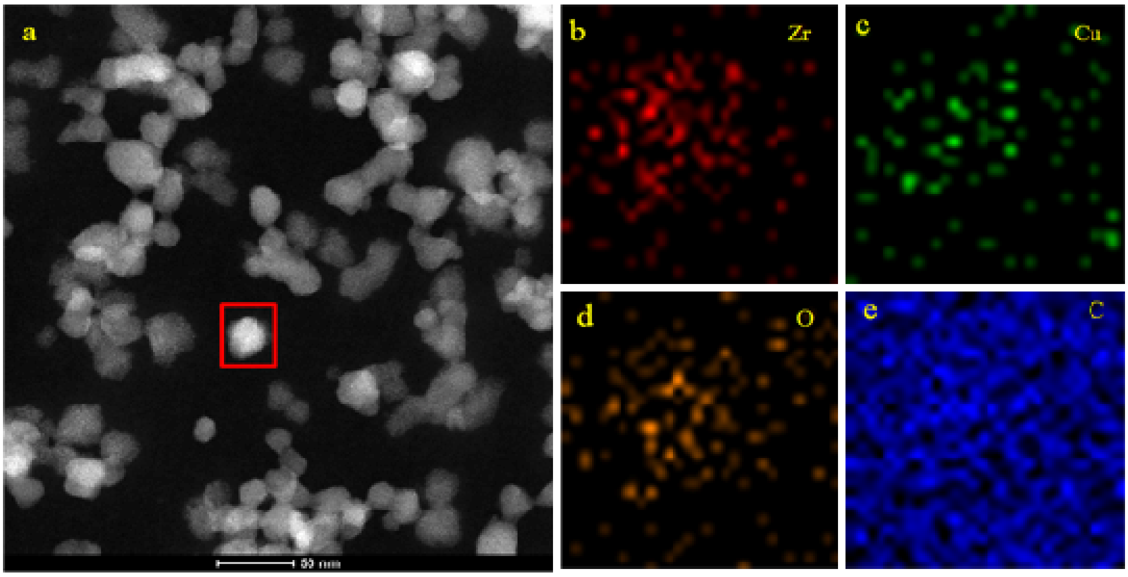

Supplementary Figure 42 STEM-HAADF image (**a**) and corresponding Zr (**b**), Cu (**c**), O (**d**), and C (**e**) elemental maps in template@H-UiO-66(Zr) prepared with MOP-*t*Bu as template precursor.

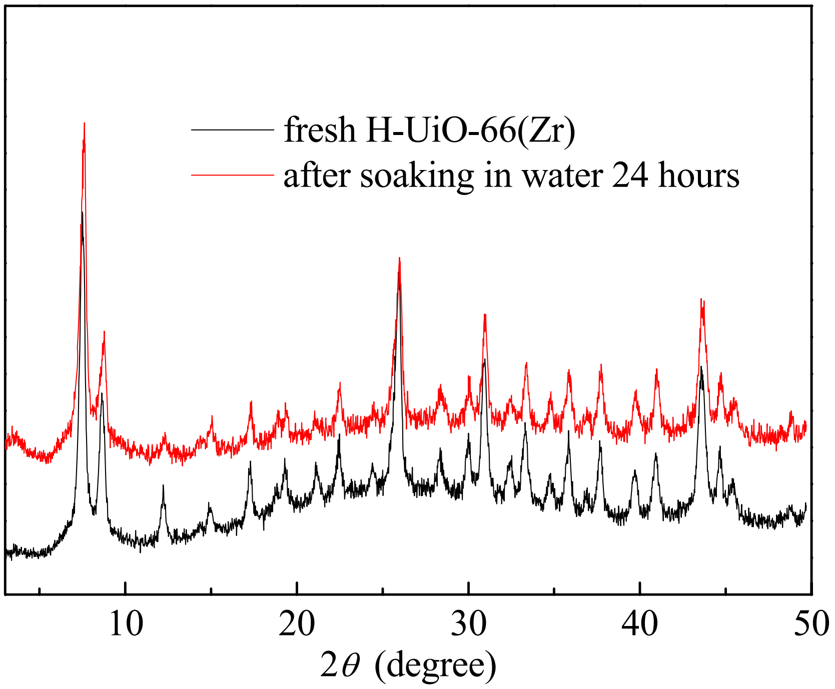

Supplementary Figure 43. PXRD patterns of H-UiO-66(Zr) before and after soaking in water for 24 hours.

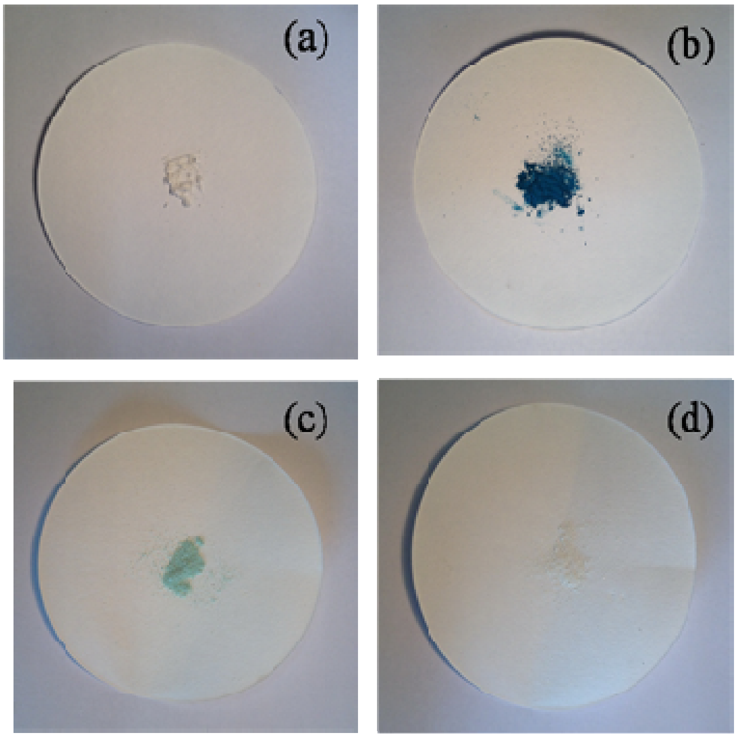

Supplementary Figure 44. Photographs of H-UiO-66(Zr) before and after bulk molecular adsorption. (**a**) fresh H-UiO-66(Zr), (**b**) H-UiO-66(Zr) (with ~40 Å mesopores) after adsorption of DB 86 , (**c**) H-UiO-66(Zr) (with ~120 Å mesopores) after adsorption of MOP-OH, and (**d**) H-UiO-66(Zr) (with ~120 Å mesopores) after adsorption of BSA.

Supplementary Tables

Supplementary Table 1. Porosity properties of various H-MOFs (prepared by using different MOAs template precursors with one-pot reaction method) and their parent MOFs.

H-MOF and MOF	MOA template precursors type	S_{BET}† ($m^2 g^{-1}$)	S_{micro}‡ ($m^2 g^{-1}$)	S_{micro}/S_{meso} §	V_t‖ ($m^2 g^{-1}$)	V_{micro}¶ ($m^2 g^{-1}$)	V_{micro}/V_{meso}#	Micro pore size (Å) *	Meso pore size** (Å)
H-ZIF-8	MOF-5	1611	1117	2.26	0.82	0.29	0.55	12.7	38.5
ZIF-8		1737	1737		0.7			11	
H-MIL-101(Cr)	ZIF-8	441	157	0.55	0.58	0.07	0.13	51	123
MIL-101(Cr)		2927	2357		1.49			29/34	
H-DUT-5	In-BPDC	1183	737	1.65	0.91	0.68	2.96	11.0	39.4
DUT-5		1652	1424		0.82			11.1	
H-UiO-66(Zr)	MOF-5	1018	671	1.93	0.65	0.27	0.71	11.8/14.1	3.82
UiO-66(Zr)		1204	1024		0.59			8/11	
H-UiO-66-NH_2(Zr)	IRMOF-3	600	327	1.19	0.49	0.14	0.38	11.8/14.7	56.2
UiO-66-NH_2(Zr)		1070	1052		0.42			7.4/9.5	
H-UiO-66-Cl(Zr)	MOF-5-Cl	736	452	1.59	0.69	0.19	0.38	15.4	77.9
UiO-66-Cl(Zr)		794	750		0.33			5.8/7.5	
H-UiO-66-NO_2(Zr)	MOF-5-NO_2	760	379	0.99	0.58	0.16	0.38	11.8/14.8	43.1
UiO-66-NO_2(Zr)		922	734		0.47			5.2/7.8	
H-UiO-66(Hf)	MOF-5	334	133	0.66	0.34	0.06	0.21	11.8/14.5	38.3
UiO-66(Hf)		890	739		0.43			8/11	
H-UiO-66-Cl(Hf)	MOF-5-Cl	327	31.3	0.11	0.30	0.02	0.07	11.7/14.0	38.2
UiO-66-Cl(Hf)		654	601		0.31			5.8/7.5	
H-UiO-66-Br(Hf)	MOF-5-Br	320	61	0.24	0.30	0.03	0.11	11.8	38.2
UiO-66-Br(Hf)		777	688		0.36			5.8/7.5	
H-UiO-66(Zr)	MOP-*t*Bu	606	438	2.61	0.36	0.18	1.00	11.8/15.4	38.3
H-UiO-66(Hf)	MOP-*t*Bu	505	171	0.51	0.77	0.09	0.12	11.2	52.5
H-UiO-66-NH_2(Hf)	MOP-*t*Bu	392	104	0.36	0.66	0.1	0.18	12.0	76
UiO-66-NH_2(Hf)		1030	933		0.46			7.4/9.5	
H-UiO-66-Br(Hf)	MOP-*t*Bu	558	221	0.66	0.50	0.11	0.29	11.0	55.5
H-UiO-66-Cl(Hf)	MOP-*t*Bu	443	112	0.34	0.66	0.13	0.25	11.0	75.6
H-UiO-66(Zr)	$Zn_4O(BC)_6$	917	300	0.49	0.87	0.13	0.17	11.8	38

H-UiO-66-NH_2(Zr)	$Zn_4O(BC)_6$	768	283	0.58	0.78	0.13	0.20	11.7	123.0
H-UiO-66-Cl(Zr)	$Zn_4O(BC)_6$	758	334	0.79	0.81	0.15	0.23	12	56.2
H-UiO-66-Br(Zr)	$Zn_4O(BC)_6$	750	429	1.34	0.55	0.18	0.49	11.7/14.7	38
UiO-66-Br(Zr)		958	783		0.47			5.8/7.5	

† S_{BET} is the BET specific surface area.

‡ S_{micro} is the *t*-plot specific micropore surface area calculated from the N_2 adsorption-desorption isotherm.

§ S_{meso} is the specific mesopore surface area estimated by subtracting S_{micro} from S_{BET}.

|| V_t is the total specific pore volume determined by using the adsorption branch of the N_2 isotherm at P/P_0 = 0.99.

¶ V_{meso} is the specific mesopore volume obtained from the BJH cumulative specific adsorption volume of pores of 1.70 to 300.00 nm in diameter.

# V_{micro} is the specific micropore volume calculated by subtracting V_{meso} from V_t.

* The micropore diameter is determined by the DFT method.

** The mesopore diameter is determined from the local maximum of the BJH distribution of pore diameters obtained in the adsorption branch of the N_2 isotherm at 77 K.

Supplementary Table 2. Element mole ratio in template@H-MOFs and H-MOFs

H-MOFs	MOA template precursors type	molar ratio of M1/M2† before acid treatment	molar ratio of M1/M2† after acid treatment	mole ratio of C/M2 in "template"§	molar ratio of C/H in H-MOF††
H-ZIF-8	MOF-5	1‡	1‡	5.99(6)||	1.05(1.14)
H-MIL-101(Cr)	ZIF-8	1.07	108.30	6.08(8)	1.57(2.0)
H-DUT-5	In-BPDC	2.47	185.65	5.68(28)	1.11(1.22)
H-UiO-66(Zr)	MOF-5	1.76	93.14	4.75(6)	1.47(1.71)
H-UiO-66-NH_2(Zr)	IRMOF-3	3.58	42.08	9.30(6)	1.25(1.41)
H-UiO-66-Cl(Zr)	MOF-5-Cl	3.29	59.19	6.16(6)	1.93(2.18)
H-UiO-66-NO_2(Zr)	MOF-5-NO_2	4.51	46.3	6.47(6)	1.83(2.18)
H-UiO-66(Hf)	MOF-5	3.72	77.91	11.6(6)	1.43(1.71)
H-UiO-66-Cl(Hf)	MOF-5-Cl	2.29	78.66	12.3(6)	1.47(2.18)
H-UiO-66-Br(Hf)	MOF-5-Br	2.34	75.01	14.87(6)	1.82(2.18)

H-UiO-66(Zr)	MOP-*t*Bu	17.89	449.37	19.78(12)	1.44(1.71)
H-UiO-66(Hf)	MOP-*t*Bu	7.98	83.60	34.46(12)	1.57(1.71)
H-UiO-66-NH_2(Hf)	MOP-*t*Bu	8.01	74.17	30.7(12)	1.25(1.41)
H-UiO-66-Br(Hf)	MOP-*t*Bu	8.77	288.67	20.12(12)	1.85(2.18)
H-UiO-66-Cl(Hf)	MOP-tBu	8.77	767.00	17.36(12)	1.81(2.18)
H-UiO-66(Zr)	$Zn_4O(BC)_6$	12.47	285.27	16.72(10.5)	1.25(1.71)
H-UiO-66-NH_2(Zr)	$Zn_4O(BC)_6$	14.38	173.96	22.4(10.5)	1.26(1.41)
H-UiO-66-Cl(Zr)	$Zn_4O(BC)_6$	9.04	567.71	14.07(10.5)	1.93(2.18)
H-UiO-66-Br(Zr)	$Zn_4O(BC)_6$	13.55	1043.99	25.41(10.5)	1.73(2.18)

† M1 is the metal iron in H-MOF and M1 is the metal iron in the template.

‡ both M1 and M2 is Zn^{2+}.

§ The mole ratio of C/M2 of the “template” in template@H-MOF can be estimated by assuming that all M1 comes from H-MOF, and H-MOF has the same element ratio as that in corresponding parent MOF. The mole ratio of C/M2 of ideal template was provided in the parentheses.

|| For both M1 = M2 = Zn^{2+}, the mole ratio of C/M2 of the “template” in template@H-ZIF-8 was estimated by assuming that all the N atoms comes from H-ZIF-8 and H-ZIF-8 has the same element ratio as that in parent ZIF-8.

††The mole ratio of C/H of ideal parent MOF was provided in the parentheses.

Supplementary Methods

All general reagents and solvents (AR grade) were commercially available and used as received except DMF, which was treated by activated 4A zeolite. Zirconium chloride ($ZrCl_4$, 98%) was purchased from Merck Schuchardt OHG. Hafnium chloride ($HfCl_4$, 99%) was purchased from Acros. Terephthalic acid (H_2BDC, 99%), and 4-methylbenzoic acid (HBC-CH_3, 98%) were purchased from Aladdin chemistry Co. Ltd. 2-methylimidazole (mIm, 99%), and 2-aminoterephthalic acid (H_2BDC-NH_2, 99%) were purchased from Alfa Aesar.

Zinc nitrate hexahydrate ($Zn(NO_3)_2·6H_2O$, 99%), zinc acetate dihydrate ($Zn(CH_3COO)_2·2H_2O$, 99%), aluminum nitrate nonahydrate ($Al(NO_3)_3·9H_2O$, 98%), coomassie brilliant blue G250 (85%), N,N′-dimethylformamide (DMF, 99.5%), and N,N′-dimethylacetamide (DMA, 99.5%) were purchased from Sinopharm Chemical Reagent Co. Ltd. Methanol (99.5%), acetone (99.5%), copper nitrate hydrate ($Cu(NO_3)_2·3H_2O$, 98%), and cupric acetate ($Cu_2(OAc)_4·2H_2O$, 98%) were purchased from Beijing Chemical Works (China). Benzoic acid (HBC, 99%), nitroterephthalic acid (H_2BDC-NO_2, 98%), 5-hydroxyisophthalic acid, (H_2BDC-OH, 99%), 5-*tert*-butyl-1,3-benzenedicarboxylic acid (H_2BDC-*t*Bu, 98%), 4,4'-biphenyldicarboxylic acid (H_2BPDC, 97%), and direct blue 86 (DB86) were purchased from TCI Shanghai. 2-bromoterephthalic acid (H_2BDC-Br, 98%), tetramethylammonium hydroxide (TMAOH, 25% in H_2O), and indium nitrate hydrate ($In(NO_3)_3·xH_2O$, 99%) were purchased from J&K Scientific Ltd. Bovine serum albumin (BSA, 98%) was purchased from Beijing Xin Jing biological technology Co. Ltd. 2-chloro-1,4-benzendicarboxylic aicd (H_2BDC-Cl) was synthesized according to the literature method[4]. Nitrogen adsorption-desorption measurements at 77 K were performed on Autosorb-IQ-MP surface area analyzer (from American Quantachrome Co. Ltd.). Prior to adsorption experiments, the samples were degassed at 150 °C for 12 hours on degassing station at vacuum. TGA data were obtained on a TGA-50 (SHIMADZU) thermogravimetric analyzer with a heating rate of 2 °C min^{-1} under N_2 atmosphere. The powder X-ray diffraction patterns (PXRD) were recorded on a BRUKER D8-Focus Bragg-Brentano X-ray Powder Diffractometer equipped with a Cu sealed tube (λ = 1.54178 Å) at room temperature. Dynamic light scattering measurements were performed with a Brookhaven BI-200SM goniometer and a Brookhaven BI-9000AT autocorrelator.

X-ray photoemission spectroscopy (XPS) spectra were recorded on a Thermo Fisher ESCALAB 250. Bruker AV 300 NMR spectrometer (300 MHz) was used for ^{13}C-NMR spectra. Elemental analysis (C, H, and N) were performed by vario EL cube (Elementar). Fourier transform infrared (FT-IR) spectra were collected in the range 2000-500 cm^{-1} on a Bruker Equinox 55 instrument. The morphologies of the MOFs were characterized using a Hitachi S-4700 scanning electron microscope (SEM) with an accelerating voltage of 20.0 kV. A Tecnai G2 F20 transmission electron microscope (FEI) equipped with an energy-dispersive X-ray spectrometer system and high-angle angular dark-field detector was used at 200 kV for high-resolution electron microscopy imaging and HAADF imaging. UV-Vis spectra measurements were performed on a TU-1901 spectrophotometer (from Beijing Purkinje General Instrument Co. Ltd.) using quartz optical cells.

Sample preparation

Preparation of nanosized MOF-5 particles, MOP-*t*Bu and MOP-OH

MOF-5 particles

Nanosized MOF-5 particles were synthesized according to the literature method[5]. H_2BDC (0.066 g, 0.4 mmol) and $Zn(CH_3COO)_2{\cdot}2H_2O$ (0.220 g, 1.0 mmol) were dissolved in 7.5 mL DMF separately. The two solutions were then mixed and stirred at room temperature for 24 hours. The resulting white suspension was isolated by centrifugation and washed with DMF at least three times to remove the unreacted raw material. Finally, nanosized MOF-5 particles were dispersed in 4 ml DMF for use in next step. Dynamic light scattering results indicate that the size of MOF-5 particles is in the range of 370~520 nm with an average value of 440 nm

(Supplementary Fig. 1). Clearly, MOF-5 crystals are typical cubic shape and no obvious particles aggregation was observed (Supplementary Fig. 2). The size of MOF-5 crystals is about 500 nm, conforming with the DLS result. The polydispersity index of DLS is 0.10, which also implies that the MOF-5 nano crystals have relative narrow particle size distribution in DMF solution.

MOP-*t*Bu

MOP-*t*Bu particles were synthesized with the scale-up method according to our previous work[6]. A DMA solution (200 mL) of H_2BDC-*t*Bu (4.450 g, 20 mmol) was mixed with a DMA solution (200 mL) of $Cu_2(OAc)_4 \cdot 2H_2O$ (4.000 g, 20 mmol) in a 500 mL beaker and the mixture was stirred for 30 minutes at room temperature. After that, 100 mL of methanol was then added in batches to the dark-blue solution and then allowed the beaker stand at room temperature. After 20 days homogeneous dark-blue block crystals of MOP-*t*Bu were collected by filtration and washed with methanol.

MOP-OH

MOP-OH particles were synthesized according to our previous work[6]. A methanol solution (100 mL) of H_2BDC-OH (3.650 g, 20 mmol) was mixed with a methanol solution (300 mL) of $Cu_2(OAc)_4 \cdot 2H_2O$ (4.000 g, 20 mmol) in a 500 mL beaker and the mixture was stirred for 30 minutes at room temperature. After that, 100 mL of DMA was then added in batches to the dark-blue solution and then allowed the beaker stand at room temperature. After 20 days homogeneous dark-blue block crystals of MOP-OH were collected by filtration and washed with DMA and a little acetone.

Preparation of H-UiO-66(Zr) with $Zn_4O(BC)_6$ as the template precursor by a two-step process

$Zn(NO_3)_2·6H_2O$ (0.148 g, 0.5 mmol) and HBC (1.830 g, 15 mmol) were dissolved in 20 mL DMF in a 100 mL Teflon liner. The mixture was sonicated to give a clear solution and the Teflon liner vessel was then sealed and placed in a preheated oven at 120 °C for 12 hours. After cooling to room temperature, $ZrCl_4$ (0.120 g, 0.5 mmol) and H_2BDC (0.166 g, 1.0 mmol) were added into the Teflon liner. After sonicated for additional 10 minutes, the Teflon liner vessel was again sealed and placed in a preheated oven at 120 °C for another 24 hours. After cooling to room temperature, the resulted solid was separated by centrifugation and washed several times with DMF. The solid was then dispersed in hydrochloric acid solution (pH = 1.0) and stirred for about 10 minutes to destroy the MOA template. The final product was isolated by centrifugation from the suspension and washed three times with DMF and acetone, respectively, to remove the decomposed template residues and dried at 60 °C overnight in oven. For its PXRD, see Supplementary Fig. 16; TGA, see Supplementary Fig. 24; N_2 adsorption-desorption isotherms, see Supplementary Fig. 3. Phase identification of H-UiO-66(Zr) was done by matching the PXRD pattern with the reference pattern of pure UiO-66(Zr)[7]. The element analysis and ICP analysis for template@H-UiO-66(Zr): Zr, 27.95, Zn 12.7, C 45.32, and H 3.15%; for H-UiO-66(Zr): Zr 31.08, Zn 0.25, C 33.12, and H 2.05%.

Preparation of H-UiO-66(Zr) with MOP-*t*Bu as template precursor by a two-step process

MOP-*t*Bu (0.200 g) was dispersed in 20 mL of DMF in a 100 mL Teflon liner. The suspension solution was sonicated for 10 minutes, then $ZrCl_4$ (0.120 g, 0.5 mmol) and H_2BDC (0.166 g, 1.0 mmol) were added into the Teflon liner. After sonicated for additional 10 minutes, the Teflon liner vessel was sealed and placed in a preheated oven at 120 °C for 24 hours. After cooling to room temperature, the resulted solid was separated by centrifugation and washed several times with DMF. Then the solid was dispersed in hydrochloric acid solution (pH = 1.0) and stirred for about 10 minutes to destroy the MOA template. The final product was isolated by centrifugation and washed three times with DMF and acetone, respectively, to remove the decomposed template residues and dried at 60 °C overnight in oven. For its PXRD, see Supplementary Fig. 17; TGA, see Supplementary Fig. 25; N_2 adsorption-desorption isotherms, see Supplementary Fig. 4. Phase identification of H-UiO-66(Zr) was done by matching the PXRD pattern with the reference pattern of pure UiO-66(Zr)[7]. The element analysis and ICP analysis for template@H-UiO-66(Zr): Zr 24.98, Cu 2.44, C 37.54, and H 2.28% ; for H-UiO-66(Zr): Zr 30.98, Cu 0.14, C 33.35, and H 1.87%.

Preparation of H-UiO-66(Zr) with MOF-5 as template precursor by a two-step process

$ZrCl_4$ (0.106 g, 0.45 mmol) and H_2BDC (0.075 g, 0.45 mmol) were dissolved in 20 mL DMF in a 100 mL Teflon liner. Then the suspension solution was sonicated for 10 minutes. After that, 2 mL of nanosized MOF-5 particles in DMF (~12 mg/g) made as above-mentioned was added into the solution and the suspension was sonicated for additional 10 minutes Finally, the Teflon liner vessel was sealed and placed in a preheated oven at 120 °C for 24

hours. After cooling to room temperature, the resulted powder was separated by centrifugation and washed several times with DMF and acetone. The solid was dispersed in hydrochloric acid solution (pH = 1.0) and stirred for about 10 minutes to destroy the acid-sensitive MOA template. The final product was isolated by centrifugation and washed three times with DMF and acetone, respectively, to remove the decomposed template residues and dried at 60 °C overnight in oven. For its PXRD, see Supplementary Fig. 18; TGA, see Supplementary Fig. 26; N_2 adsorption-desorption isotherms, see Supplementary Fig. 5. Phase identification of H-UiO-66(Zr) was done by matching the PXRD pattern with the reference pattern of pure UiO-66(Zr)[7]. The element analysis and ICP analysis for template@H-UiO-66(Zr): Zr 27.14, Zn 11.05, C 35.65%, and H 1.57%; for H-UiO-66(Zr): Zr 31.24, Zn 0.24, C 33.49, and H 2.27%.

Preparation of H-MOFs with $Zn_4O(BC)_6$ precursors as templates by one-pot reaction method

H-UiO-66(Zr)

$Zn(NO_3)_2{\cdot}6H_2O$ (0.148 g, 0.5 mmol), HBC (1.830 g, 15 mmol), $ZrCl_4$ (0.120 g, 0.5 mmol), and H_2BDC (0.166 g, 1.0 mmol) were dissolved in 20 mL DMF in a 100 mL Teflon liner. The mixture was sonicated for 10 minutes. Then the Teflon liner vessel was sealed and placed in a preheated oven at 120 °C for 24 hours. After cooling to room temperature, the resulted powder was separated by centrifugation and washed several times with DMF. The resulted solid was then dispersed in hydrochloric acid solution (pH = 1.0) and stirred for about 10 minutes to destroy the acid-sensitive MOA template. The final product was isolated by

centrifugation and washed three times with DMF and acetone, respectively, to remove the decomposed template residues and dried at 60 °C overnight in oven. For its PXRD, see Supplementary Fig. 20a; TGA, see Supplementary Fig. 27a; N_2 adsorption-desorption isotherms, see Supplementary Fig. 10a. Phase identification of H-UiO-66(Zr) was done by matching the PXRD pattern with the reference pattern of pure UiO-66(Zr)[7]. The element analysis and ICP analysis for template@H-UiO-66(Zr): Zr 31.94, Zn 1.82, C 39.20, and H 3.26%,; for H-UiO-66(Zr): Zr 32.94, Zn, 0.08, C 32.07, and H 2.15%.

H-UiO-66-NH_2(Zr)

$Zn(NO_3)_2 \cdot 6H_2O$ (0.148 g, 0.5 mmol), HBC (1.830 g, 15 mmol), $ZrCl_4$ (0.120 g, 0.5 mmol), and H_2BDC-NH_2 (0.181 g, 1.0 mmol) were dissolved in 20 mL DMF in a 100 mL Teflon liner. The mixture was sonicated to give a clear solution. Then the Teflon liner vessel was sealed and placed in a preheated oven at 120 °C for 24 hours. After cooling to room temperature, the resulted powder was separated by centrifugation and washed several times with DMF. The resulted solid was dispersed in hydrochloric acid solution (pH = 1.0) and stirred for about 10 minutes to destroy the acid-sensitive MOA template. The final product was isolated by centrifugation and washed three times with DMF and acetone, respectively, to remove the decomposed template residues and dried at 60 °C overnight in oven. For its PXRD, see Supplementary Fig. 20b; TGA, see Supplementary Fig. 27b; N_2 adsorption-desorption isotherms, see Supplementary Fig. 10b. Phase identification of H-UiO-66-NH_2(Zr) was done by matching the PXRD pattern with the reference pattern of pure UiO-66-NH_2(Zr)[8]. The element analysis and ICP analysis for template@H-UiO-66-NH_2(Zr): Zr 28.35, Zn 1.41, C 35.64, and H 2.36%; for H-UiO-66-NH_2(Zr): Zr 29.79, Zn 0.12, C 25.64, and H 1.71%.

H-UiO-66-Cl(Zr)

$Zn(NO_3)_2{\cdot}6H_2O$ (0.148 g, 0.5 mmol), HBC (1.830 g, 15 mmol), $ZrCl_4$ (0.120 g, 0.5 mmol), and H_2BDC-Cl (0.200 g, 1.0 mmol) were dissolved in 20 mL DMF in a 100 mL Teflon liner. The mixture was sonicated to give a clear solution. Then the Teflon liner vessel was sealed and placed in a preheated oven at 120 °C for 24 hours. After cooling to room temperature, the resulted powder was separated by centrifugation and washed several times with DMF. Then the solid was dispersed in hydrochloric acid solution (pH = 1.0) and stirred for about 10 minutes to destroy the acid-sensitive MOA template. The final product was isolated by centrifugation and washed three times with DMF and acetone, respectively, to remove the decomposed template residues and dried at 60 °C overnight in oven. For its PXRD, see Supplementary Fig. 20c; TGA, see Supplementary Fig. 27c; N_2 adsorption-desorption isotherms, see Supplementary Fig. 10c. Phase identification of H-UiO-66-Cl(Zr) was done by matching the PXRD pattern with the reference pattern of pure UiO-66-Cl(Zr)[9]. The element analysis and ICP analysis for template@H-UiO-66-Cl(Zr): Zr 27.98, Zn 2.22 ,C 35.18, and H 1.81%; for H-UiO-66-Cl (Zr): Zr 28.45, Zn 0.04, C 28.92, and H 1.26%.

H-UiO-66-Br(Zr)

$Zn(NO_3)_2{\cdot}6H_2O$ (0.148 g, 0.5 mmol), HBC (1.830 g, 15 mmol), $ZrCl_4$ (0.120 g, 0.5 mmol), and H_2BDC-Br (0.243 g, 1.0 mmol) were dissolved in 20 mL DMF in a 100 mL Teflon liner. The mixture was sonicated to give a clear solution. Then the Teflon liner vessel was sealed and placed in a preheated oven at 120 °C for 24 hours. After cooling to room temperature, the resulted powder was separated by centrifugation and washed several times with DMF. Then the solid was dispersed in hydrochloric acid solution (pH = 1.0) and stirred

for about 10 minutes to destroy the acid-sensitive MOA template. The final product was isolated by centrifugation and washed three times with DMF and acetone, respectively, to remove the decomposed template residues and dried at 60 °C overnight in oven. For its PXRD, see Supplementary Fig. 20d; TGA, see Supplementary Fig. 27d; N_2 adsorption-desorption isotherms, see Supplementary Fig. 10d. Phase identification of H-UiO-66-Br(Zr) was done by matching the PXRD pattern with the reference pattern of pure UiO-66-Br (Zr)[8]. The element analysis and ICP analysis for template@H-UiO-66-Br(Zr): Zr 24.41, Zn 1.29, C 31.72, and H 2.43% for H-UiO-66-Br(Zr): Zr 25.10%, Zn 0.02%, C 24.48%, and H 1.19%.

Preparation of H-MOFs with MOP-*t*Bu precursors as template by one-pot reaction method

H-UiO-66(Zr)

H_2BDC-*t*Bu (0.089 g, 0.4 mmol), $Cu(NO_3)_2 \cdot 3H_2O$ (0.097 g, 0.5 mmol), $ZrCl_4$ (0.120 g, 0.5 mmol), and H_2BDC (0.166 g, 1.0 mmol) were dispersed in 20 mL DMF in a 100 mL Teflon liner. The mixture was sonicated for 10 minutes. Then the Teflon liner vessel was sealed and placed in a preheated oven at 120 °C for 24 hours. After cooling to room temperature, the resulted powder was separated by centrifugation and washed several times with DMF. Then the solid was dispersed in hydrochloric acid solution (pH = 1.0) and stirred for about 10 minutes to destroy the acid-sensitive MOA template. The final product was isolated by centrifugation and washed three times with DMF and acetone, respectively, to remove the decomposed template residues and dried at 60 °C overnight in oven. For its PXRD, see Supplementary Fig. 21a; TGA, see Supplementary Fig. 28a; N_2 adsorption-desorption

isotherms, see Supplementary Fig. 11a. Phase identification of H-UiO-66(Zr) was done by matching the PXRD pattern with the reference pattern of pure UiO-66(Zr)[7]. The element analysis and ICP analysis for template@H-UiO-66(Zr): Zr 31.21, Cu 1.22, C 37.46, and H 2.39%; for H-UiO-66(Zr): Zr 31.71, Cu 0.05, C 33.2, and H 1.93%.

H-UiO-66(Hf)

H_2BDC-*t*Bu (0.089 g, 0.4 mmol), $Cu(NO_3)_2 \cdot 3H_2O$ (0.097 g, 0.5 mmol), $HfCl_4$ (0.160 g, 0.5 mmol), and H_2BDC (0.166 g, 1.0 mmol) were dispersed in 5 mL DMF in a 20 mL Teflon liner. Then the mixture was sonicated for 10 minutes and the Teflon liner vessel was sealed and placed in a preheated oven at 100 °C for 24 hours. After cooling to room temperature, the resulted powder was separated by centrifugation and washed several times with DMF. Then the solid was dispersed in hydrochloric acid solution (pH = 1.0) and stirred for about 10 minutes to destroy the acid-sensitive MOA template. The final product was isolated by centrifugation and washed three times with DMF and acetone, respectively, to remove the decomposed template residues and dried at 60 °C overnight in oven. For its PXRD, see Supplementary Fig. 21b; TGA, see Supplementary Fig. 28b; N_2 adsorption-desorption isotherms, see Supplementary Fig. 11b. Phase identification of H-UiO-66(Hf) was done by matching the PXRD pattern with the reference pattern of pure UiO-66(Hf)[10]. The element analysis and ICP analysis for template@H-UiO-66(Hf): Hf 37.15, Cu 1.65, C 30.72, H 3.14%; for H-UiO-66(Hf): Hf 46.75, Cu 0.19, C 25.78, and H 1.38%.

H-UiO-66-NH_2(Hf)

H_2BDC-*t*Bu (0.067 g, 0.3 mmol), $Cu(NO_3)_2 \cdot 3H_2O$ (0.075 g, 0.4 mmol), $HfCl_4$ (0.16 g, 1.0 mmol), and H_2BDC-NH_2 (0.181 g, 1.0 mmol) were dispersed in 5 mL DMF in a 20 mL

Teflon liner. Then the mixture was sonicated for 10 minutes and the Teflon liner vessel was sealed and placed in a preheated oven at 100 °C for 24 hours. After cooling to room temperature, the resulted powder was separated by centrifugation and washed several times with DMF. Then the solid was dispersed in hydrochloric acid solution (pH = 1.0) and stirred for about 10 minutes to destroy the acid-sensitive MOA template. The final product was isolated by centrifugation and washed three times with DMF and acetone, respectively, to remove the decomposed template residues and dried at 60 °C overnight in oven. For its PXRD, see Supplementary Fig. 21c; TGA, see Supplementary Fig. 28c; N_2 adsorption-desorption isotherms, see Supplementary Fig. 11c. Phase identification of H-UiO-66-NH_2(Hf) was done by matching the PXRD pattern with the reference pattern of UiO-66(Hf)[10]. The element analysis and ICP analysis for template@H-UiO-66-NH_2(Hf): Hf 35.49, Cu 1.56, C 28.13, and H 1.927%; for H-UiO-66-NH_2(Hf): Hf 45.55, Cu 0.21, C 24.55, and H 1.65%.

H-UiO-66-Cl(Hf)

H_2BDC-*t*Bu (0.089 g, 0.4 mmol), $Cu(NO_3)_2 \cdot 3H_2O$ (0.097 g, 0.5 mmol), $HfCl_4$ (0.160 g, 1.0 mmol), and H_2BDC-Cl (0.200 g, 1.0 mmol) were dispersed in 5 mL DMF in a 20 mL Teflon liner. Then the mixture was sonicated 10 minutes and the Teflon liner vessel was sealed and placed in a preheated oven at 100 °C for 24 hours. After cooling to room temperature, the resulted powder was separated by centrifugation and washed several times with DMF. The result product was dispersed in hydrochloric acid solution (pH = 1.0) and stirred for about 10 minutes to destroy the acid-sensitive MOA template. The final product was isolated by centrifugation and washed three times with DMF and acetone, respectively, to remove the decomposed template residues and dried at 60 °C overnight in oven. For its PXRD,

see Supplementary Fig. 21d; TGA, see Supplementary Fig. 28d; N_2 adsorption-desorption isotherms, see Supplementary Fig. 11d. Phase identification of H-UiO-66-Cl(Hf) was done by matching the PXRD pattern with the reference pattern of parent UiO-66(Hf)[10]. The element analysis and ICP analysis for template@H-UiO-66-Cl(Hf): Hf 33.27, Cu 1.35, C 22.32, and H 1.87%; for H-UiO-66-Cl(Hf): Hf 41.43, Cu 0.05, C 22.19, and H 1.03%.

H-UiO-66-Br(Hf)

H_2BDC-*t*Bu (0.089 g, 0.4 mmol), $Cu(NO_3)_2 \cdot 3H_2O$ (0.097 g, 0.5 mmol), $HfCl_4$ (0.160 g, 1.0 mmol), and H_2BDC-Br (0.243 g, 1.0 mmol) were dispersed in 5 mL DMF in a 20 mL Teflon liner. Then the mixture was sonicated for 10 minutes and the Teflon liner vessel was sealed and placed in a preheated oven at 100 °C for 24 hours. After cooling to room temperature, the resulted powder was separated by centrifugation and washed several times with DMF. Then the solid was dispersed in hydrochloric acid solution (pH = 1.0) and stirred for about 10 minutes to destroy the acid-sensitive MOA template. The final product was isolated by centrifugation and washed three times with DMF and acetone, respectively, to remove the decomposed template residues and dried at 60 °C overnight in oven. For its PXRD, see Supplementary Fig. 21e; TGA, see Supplementary Fig. 28e; N_2 adsorption-desorption isotherms, see Supplementary Fig. 11e. Phase identification of H-UiO-66-Br(Hf) was done by matching the PXRD pattern with the reference pattern of UiO-66(Hf)[10]. The element analysis and ICP analysis for template@H-UiO-66-Br(Hf): Hf 31.41, Cu 1.27, C 21.72, and H 1.80%; for H-UiO-66-Br(Hf): Hf 38.08, Cu 0.018, C 20.96, and H 0.95%.

Preparation of H-MOFs with MOFs precursors (MOF-5, IRMOF-3, MOF-5-NO_2,

MOF-5-Cl, MOF-5-Br, In-BPDC, and ZIF-8) as template by one-pot reaction method

H-UiO-66(Zr) by MOF-5 precursors

$Zn(NO_3)_2·6H_2O$ (0.297 g, 1.0 mmol), $ZrCl_4$ (0.120 g, 0.5 mmol), and H_2BDC (0.166 g, 1.0 mmol) were dissolved in 20 mL DMF in a 100 mL Teflon liner. Then the mixture was sonicated for 10 minutes. Finally, the Teflon liner vessel was sealed and placed in a preheated oven at 120 °C for 24 hours. After cooling to room temperature, the resulted powder was separated by centrifugation and washed several times with DMF and acetone. The solid was dispersed in hydrochloric acid solution (pH = 1.0) and stirred for about 10 minutes to destroy the acid-sensitive MOA template. The final product was isolated by centrifugation and washed three times with DMF and acetone, respectively, to remove the decomposed template and dried at 60 °C overnight in oven. For its PXRD, see Supplementary Fig. 22a; TGA, see Supplementary Fig. 29a; N_2 adsorption-desorption isotherms, see Supplementary Fig. 12a. Phase identification of H-UiO-66(Zr) was done by matching the PXRD pattern with the reference pattern of pure UiO-66(Zr)[7]. The element analysis and ICP analysis for template@H-UiO-66(Zr): Zr 29.27, Zn 8.89, C 38.56, and H 1.55%; for H-UiO-66(Zr): Zr 31.78, Zn 0.22, C 32.99, and H 1.88%.

H-UiO-66-NH_2(Zr) by IRMOF-3[11] precursors

$Zn(NO_3)_2·6H_2O$ (0.223 g, 0.75 mmol), $ZrCl_4$ (0.120 g, 0.5 mmol), and $H_2BDC-NH_2$ (0.181 g, 1.0 mmol) were dissolved in 20 mL DMF in a 100 mL Teflon liner. Then the mixture was sonicated for 10 minutes. Finally, the Teflon liner vessel was sealed and placed in a preheated oven at 120 °C for 24 hours. After cooling to room temperature, the resulted powder was separated by centrifugation and washed several times with DMF and acetone.

The solid was dispersed in hydrochloric acid solution (pH = 1.0) and stirred for about 10 minutes to destroy the acid-sensitive MOA template. The final product was isolated by centrifugation and washed three times with DMF and acetone, respectively, to remove the decomposed template and dried at 60 °C overnight in oven. For its PXRD, see Supplementary Fig. 22b; TGA, see Supplementary Fig. 29b; N_2 adsorption-desorption isotherms, see Supplementary Fig. 12b. Phase identification of H-UiO-66-NH_2(Zr) was done by matching the PXRD pattern with the reference pattern of pure UiO-66-NH_2(Zr)[8]. The element analysis and ICP analysis for template@H-UiO-66-NH_2(Zr): Zr 21.74, Zn 4.34, C 30.28, and H 1.81%; for H-UiO-66-NH_2 (Zr): Zr 27.38, Zn 0.46, C 25.79, and H 1.74%.

H-UiO-66-NO_2(Zr) by MOF-5-NO_2[12] precursors

$Zn(NO_3)_2{\cdot}6H_2O$ (0.223 g, 0.75 mmol), $ZrCl_4$ (0.120 g, 0.5 mmol), and H_2BDC-NO_2 (0.211 g, 1.0 mmol) were dissolved in 20 mL DMF in a 100 mL Teflon liner. Then the mixture was sonicated for 10 minutes. Finally, the Teflon liner vessel was sealed and placed in a preheated oven at 120 °C for 24 hours. After cooling to room temperature, the resulted powder was separated by centrifugation and washed several times with DMF and acetone. The solid was dispersed in hydrochloric acid solution (pH = 1.0) and stirred for about 10 minutes to destroy the acid-sensitive MOA template. The final product was isolated by centrifugation and washed three times with DMF and acetone, respectively, to remove the decomposed template and dried at 60 °C overnight in oven. For its PXRD, see Supplementary Fig. 22c; TGA, see Supplementary Fig. 29c; N_2 adsorption-desorption isotherms, see Supplementary Fig. 12c. Phase identification of H-UiO-66-NO_2(Zr) was done by matching the PXRD pattern with the reference pattern of pure UiO-66-NO_2(Zr)[8]. The element analysis

and ICP analysis for template@H-UiO-66-NO_2(Zr): Zr 21.85, Zn 3.47, C 28.18, and H 1.07%; for H-UiO-66-NO_2(Zr): Zr 26.43, Zn 0.40, C 25.64, and H 1.18%.

H-UiO-66-Cl(Zr) by MOF-5-Cl[13] precursors

$Zn(NO_3)_2{\cdot}6H_2O$ (0.148 g, 0.5 mmol), $ZrCl_4$ (0.120 g, 0.5 mmol), and H_2BDC-Cl (0.200 g, 1.0 mmol) were dissolved in 20 mL DMF in a 100 mL Teflon liner. Then the mixture was sonicated for 10 minutes. Finally, the Teflon liner vessel was sealed and placed in a preheated oven at 120 °C for 24 hours. After cooling to room temperature, the resulted powder was separated by centrifugation and washed several times with DMF and acetone. Then the solid was dispersed in hydrochloric acid solution (pH = 1.0) and stirred for about 10 minutes to destroy the acid-sensitive MOA template. The final product was isolated by centrifugation and washed three times with DMF and acetone, respectively, to remove the decomposed template and dried at 60 °C overnight in oven. For its PXRD, see Supplementary Fig. 22d; TGA, see Supplementary Fig. 29d; N_2 adsorption-desorption isotherms, see Supplementary Fig. 12d. Phase identification of H-UiO-66-Cl(Zr) was done by matching the PXRD pattern with the reference pattern of pure UiO-66-Cl(Zr)[9]. The element analysis and ICP analysis for template@H-UiO-66-Cl(Zr): Zr 22.45, Zn 4.90, C 29.17, and H 1.27%; for H-UiO-66-Cl(Zr): Zr 28.14, Zn 0.34, C 28.49, and H 1.27%.

H-UiO-66(Hf) by MOF-5 precursors

$Zn(NO_3)_2{\cdot}6H_2O$ (0.148 g, 0.5 mmol), $HfCl_4$ (0.160 g, 0.5 mmol), and H_2BDC (0.166 g, 1.0 mmol) were dispersed in 5 mL of DMF in a 20 mL Teflon liner. Then the mixture was sonicated for 10 minutes. The Teflon liner vessel was sealed and placed in a preheated oven at 100 °C for 24 hours. After cooling to room temperature, the resulted powder was separated by

centrifugation and washed several times with DMF. The resulted solid was dispersed in hydrochloric acid solution (pH = 1.0) and stirred for about 10 minutes to destroy the acid-sensitive MOA template. The final product was isolated by centrifugation and washed three times with DMF and acetone, respectively, to remove the decomposed template residues and dried at 60 °C overnight in oven. For its PXRD, see Supplementary Fig. 22e; TGA, see Supplementary Fig. 29e; N_2 adsorption-desorption isotherms, see Supplementary Fig. 12e. Phase identification of H-UiO-66(Hf) was done by matching the PXRD pattern with the reference pattern of pure UiO-66(Hf)[10]. The element analysis and ICP analysis for template@H-UiO-66(Hf): Hf 35.15, Zn 3.46, C 26.28, and H 1.89%; for H-UiO-66(Hf): Hf 47.41, Zn 0.22, C 23.59, and H 1.39%.

H-UiO-66-Br(Hf) by MOF-5-Br[13] precursors

$Zn(NO_3)_2 \cdot 6H_2O$ (0.148 g, 0.5 mmol), $HfCl_4$ (0.160 g, 0.5 mmol), and H_2BDC-Br (0.243 g, 1.0 mmol) were dispersed in 5 mL DMF in a 20 mL Teflon liner. Then the mixture was sonicated for 10 minutes. Then the Teflon liner vessel was sealed and placed in a preheated oven at 100 °C for 24 hours. After cooling to room temperature, the resulted powder was separated by centrifugation and washed several times with DMF. Then the solid was dispersed in hydrochloric acid solution (pH = 1.0) and stirred for about 10 minutes to destroy the acid-sensitive MOA template. The final product was isolated by centrifugation and washed three times with DMF and acetone, respectively, to remove the decomposed template residues and dried at 60 °C overnight in oven. For its PXRD, see Supplementary Fig. 22f; TGA, see Supplementary Fig. 29f; N_2 adsorption-desorption isotherms, see Supplementary Fig. 12f. Phase identification of H-UiO-66-Br(Hf) was done by matching the PXRD pattern with the

reference pattern of unmodified UiO-66(Hf)[10]. The element analysis and ICP analysis for template@H-UiO-66-Br(Hf): Hf 23.93, Zn 3.75, C 23.11, and H 1.57%; for H-UiO-66-Br(Hf): Hf 38.32, Zn 0.18, C 19.88, and H 0.92%.

H-UiO-66-Cl(Hf) by MOF-5-Cl[13] precursors

$Zn(NO_3)_2·6H_2O$ (0.148 g, 0.5 mmol), $HfCl_4$ (0.160 g, 0.5 mmol), and H_2BDC-Cl (0.200 g, 1.0 mmol) were dispersed in 5 mL of DMF in a 20 mL Teflon liner. Then the mixture was sonicated for 10 minutes. The Teflon liner vessel was sealed and placed in a preheated oven at 100 °C for 24 hours. After cooling to room temperature, the resulted powder was separated by centrifugation and washed several times with DMF. The resulted solid was dispersed in hydrochloric acid solution (pH = 1.0) and stirred for about 10 minutes to destroy the acid-sensitive MOA template. The final product was isolated by centrifugation and washed three times with DMF and acetone, respectively, to remove the decomposed template residues and dried at 60 °C overnight in oven. For its PXRD, see Supplementary Fig. 22g; TGA, see Supplementary Fig. 29g; N_2 adsorption-desorption isotherms, see Supplementary Fig. 12g. Phase identification of H-UiO-66-Cl(Hf) was done by matching the PXRD pattern with the reference pattern of unmodified UiO-66(Hf)[10]. The element analysis and ICP analysis for template@H-UiO-66-Cl(Hf): Hf 27.94, Zn 4.46, C 25.17, and H 2.58%; for H-UiO-66-Cl(Hf): Hf 43.25, Zn 0.20, C 22.38, and H 1.28%.

H-DUT-5 by In-BPDC[14] precursors

$In(NO_3)_2·xH_2O$ (0.700 g, 2.32 mmol), 520 mg $Al(NO_3)_3·9H_2O$(0.520 g, 1.386 mmol), and H_2BPDC (0.260 g, 1.07 mmol) were dispersed in 30 mL of DMF in a 100 mL Teflon liner. Then the mixture was sonicated for 10 minutes. The Teflon liner vessel was sealed and placed

in a preheated oven at 120 °C for 24 hours. After cooling to room temperature, the resulted powder was separated by centrifugation and washed several times with DMF. The solid was dispersed in hydrochloric acid solution (pH = 1.0) and stirred for about 10 minutes to destroy the acid-sensitive MOA template. The final product was isolated by centrifugation and washed three times with DMF and acetone, respectively, to remove the decomposed template residue and dried at 60 °C overnight in oven. For its PXRD, see Supplementary Fig. 22h; TGA, see Supplementary Fig. 29h; N_2 adsorption-desorption isotherms, see Supplementary Fig. 12h. Phase identification of H-DUT-5 was done by matching the PXRD pattern with the reference pattern of DUT-5[15]. The element analysis and ICP analysis for template@H-DUT-5: Al, 8.22, In 14.13, C 59.77, and H 2.95%; for H-DUT-5: Al 9.07, In 0.20, C 54.25, and H 4.11%.

H-ZIF-8 by MOF-5 precursors

H_2BDC (0.200 g, 1.2 mmol), $Zn(NO_3)_2{\cdot}6H_2O$ (0.200 g, 0.673 mmol), and mIm (0.438 g, 5.335 mmol) were dispersed in 60 mL of DMF in a 100 mL beaker. Then the solution was sonicated for 10 minutes and 1 ml of triethylamine was added in the solution. After stiring for 24 hours, the resulted powder was separated by centrifugation and washed several times with DMF. The result product was dispersed in sodium hydroxide solution (pH = 14.0) and stirred for about 10 minutes to destroy the acid-sensitive MOA template. The final product was isolated by centrifugation and washed three times with H_2O and DMF and acetone, respectively, to remove the decomposed template residues and dried at 60 °C overnight in oven. For its PXRD, see Supplementary Fig. 22i; TGA, see Supplementary Fig. 29i; N_2 adsorption-desorption isotherms, see Supplementary Fig. 12i. Phase identification of H-ZIF-8

was done by matching the PXRD pattern with the reference pattern of ZIF-8[16]. The element analysis and ICP analysis for template@H-ZIF-8: Zn 32.24, C40.47, and H 2.03%; for H-ZIF-8: Zn 28.04, C 41.47, and H 3.33%.

H-MIL-101(Cr) by ZIF-8[16] precursors

$Zn(NO_3)_2{\cdot}6H_2O$ (0.400 g, 1.347 mmol), mIm (0.2 g, 2.436 mmol), $Cr(NO_3)_3{\cdot}9H_2O$ (0.400 g, 1.0mmol), and H_2BDC (0.166 g, 1.0 mmol) were dispersed in 7 mL of alkali solution (0.05 mmol/L TMAOH) in a 20 mL Teflon liner. Then, the mixture was stirred for 10 minutes and the Teflon liner vessel was sealed and placed in a preheated oven at 210 °C for 24 hours. After cooling to room temperature, the resulted powder was separated by centrifugation and washed several times with DMF. The solid was dispersed in hydrochloric acid solution (pH = 1.0) and stirred for about 10 minutes to destroy the acid-sensitive MOA template. The final product was isolated by centrifugation and washed three times with DMF and acetone, respectively, to remove the decomposed template residues and dried at 60 °C overnight in oven. For its PXRD, see Supplementary Fig. 22j; TGA, see Supplementary Fig. 29j; N_2 adsorption-desorption isotherms, see Supplementary Fig. 12j Phase identification of H-MIL-101(Cr) was done by matching the PXRD pattern with the reference pattern of MIL-101(Cr)[17]. The element analysis for template@H-MIL-101(Cr): Cr 12.74, Zn 14.95, C 40.21, and H 2.88%; for H-MIL-101(Cr): Cr 21.49, Zn, 0.25, C 38.38, and H 2.05%.

Liquid Phase Adsorption Experiments of H-UiO-66(Zr)

DB 86 adsorption

Before adsorption, the adsorbents were dried overnight under vacuum at 120 ˚C and were

kept in a desiccator. 0.010 g (precisely weighed) of activated H-MOF and 20 ml DB 86 aqueous solution (500 mg/L) were added in 30 ml vial with a good sealed screw cap. After shaking for a pre-determined time (between 0.5 hour and 72 hours) at 25 °C, the solution was separated from the adsorbents with a syringe filter (polyether sulfone millipore filter film, 0.22 μm).Then the residual concentration of DB 86 in solution was determined at λ = 336 nm by UV-Vis spectrophotometer (see Fig. 4a in text).

MOP-OH adsorption

0.100 g (precisely weighed) of activated H-MOF and 5 ml MOP-OH methanol solution (1000 mg/L) were added in 8 ml vial with a good sealed screw cap. After shaking for a pre-determined time (between 0.5 hour and 72 hours) at 25 °C, the slurry was centrifuged at 10000 rpm for 10 minutes. The residual concentration of MOP-OH solution was determined at λ = 690.5-695 nm by UV-Vis spectrophotometer (see Fig. 4b in text).

BSA adsorption

0.010 g (precisely weighed) of activated H-MOF and 50 ml BSA aqueous solution (100 mg/L) in the presence of 0.9% NaCl were added in 75 ml conical flask. After shaking for a pre-determined time (between 0.5 hour and 72 hours) at 25 °C, the slurry was centrifuged at 10000 rpm for 10 minutes. The residual concentration of BSA solution was determined by Coomassie brilliant blue method at λ = 595 nm using UV-Vis spectrophotometer (see Fig. 4c in text).

Supplementary References

1 Garibay, S. J. & Cohen, S. M., Isoreticular synthesis and modification of frameworks with the UiO-66 topology. *Chem. Commun.* **46**, 7700-7702 (2010)

2 Rada, Z. H., Abid, H. R., Sun, H. & Wang, S., Bifunctionalized metal organic frameworks, UiO-66-NO_2-X (X = -NH_2, -$(OH)_2$, or -$(COOH)_2$), for enhanced adsorption and selectivity of CO_2 and N_2. *J. Chem. Eng. Data* **60**, 2152-2161 (2015)

3 DeCoste, J. B. *et al.* Stability and degradation mechanisms of metal-organic frameworks containing the $Zr_6O_4(OH)_4$ secondary building unit. *J. Mate. Chem. A* **1**, 5642-5650 (2013)

4 Liang, W., Babarao, R. & D′Alessandro, D. M. Microwave-assisted solvothermal synthesis and optical properties of tagged MIL-140A metal−organic frameworks. *Inorg. Chem.* **52**, 12878-12880 (2013).

5 Guo, H. *et al.* Combining coordination modulation with acid−base adjustment for the control over size of metal−organic frameworks. *Chem. Mater.* **24**, 444-450 (2012).

6 Li, J.-R. & Zhou, H.-C. Bridging-ligand-substitution strategy for the preparation of metal−organic polyhedra. *Nature Chem.* **2**, 893-898 (2010).

7 Cavka, J. H. *et al.* A new zirconium inorganic building brick forming metal organic frameworks with exceptional stability. *J. Am. Chem. Soc.* **130**, 13850-13851 (2008).

8 Kandiah, M. *et al.* Synthesis and stability of tagged UiO-66 Zr-MOFs. *Chem. Mater.* **22**, 6632-6640 (2010).

9 Biswas, S. & Van Der Voort, P. A general strategy for the synthesis of functionalised UiO-66 frameworks: characterisation, stability and CO_2 adsorption properties. *Eur. J. Inorg. Chem.* **2013**, 2154-2160 (2013).

10 Gianolio, D. *et al.* Structural determination of a highly stable metal-organic framework with possible application to interim radioactive waste scavenging: Hf-UiO-66. *Phys. Rev. B* **86**, 125429 (2012).

11 Eddaoudi, M. *et al.* Systematic design of pore size and functionality in isoreticular MOFs and their application in methane storage. *Science* **295**, 469-472 (2002).

12 Yi, Z. *et al.* Synthesis of nitro-modified MOF-5 and its application on catalyzing the thermal decomposition of carbamates. *Chem. J. Chinese. U.* **35**, 613-618 (2014).

13 Yang, J., Grzech, A., Mulder, F. M. & Dingemans, T. J. The hydrogen storage capacity of mono-substituted MOF-5 derivatives: An experimental and computational approach. *Micropor. Mesopor. Mat.* **171**, 65-71 (2013).

14 Gu, J., Kim, S., Kim, Y. & Huh, S. Structural isomerism of an anionic nanoporous In-MOF with interpenetrated diamond-like topology. *CrystEngComm* **14**, 1819-1824 (2012)

15 Senkovska, I. *et al.* New highly porous aluminium based metal-organic frameworks: Al(OH)(ndc) (ndc = 2,6-naphthalene dicarboxylate) and Al(OH)(bpdc) (bpdc = 4, 4′-biphenyl dicarboxylate). *Micropor. Mesopor. Mat.* **122**, 93-98 (2009).

16 Park, K. S. *et al.* Exceptional chemical and thermal stability of zeolitic imidazolate frameworks. *Proc. Natl. Acad. Sci. U.S.A.* **103**, 10186-10191 (2006).

17 Férey, G. *et al.* A chromium terephthalate-based solid with unusually large pore volumes and surface area. *Science* **309**, 2040-2042 (2005).
